# Synthesis and Photophysical Properties of Polycarbo-Substituted Quinazolines Derived from the 2-Aryl-4-chloro-6-iodoquinazolines

**DOI:** 10.3390/molecules200814656

**Published:** 2015-08-13

**Authors:** Malose Jack Mphahlele, Hugues Kamdem Paumo, Lydia Rhyman, Ponnadurai Ramasami

**Affiliations:** 1 Department of Chemistry, College of Science, Engineering and Technology, University of South Africa, P.O. Box 392, Pretoria 0003, South Africa; E-Mail: tkademph@unisa.ac.za; 2 Computational Chemistry Group, Department of Chemistry, Faculty of Science, University of Mauritius, Réduit 80837, Mauritius; E-Mails: lyd.rhyman@gmail.com (L.R.); ramchemi@intnet.mu (P.R.)

**Keywords:** 2-aryl-4-chloro-8-iodoquinazolines, cross-coupling reactions, polycarbo-substituted quinazolines, photophysical properties

## Abstract

The reactivity of the 2-aryl-4-chloro-6-iodoquinazolines towards palladium catalyzed sequential (Sonogashira/Suzuki-Miyaura) and one-pot two-step cross-coupling (bis-Sonogashira, and successive Sonogashira/Stille) reactions to afford novel unsymmetrical polycarbo-substituted quinazolines has been evaluated. In contrast to the chloro-bromo substituted quinazolines in which selectivity has been previously found to generally favor substitution at the more activated C(4)-Cl bond over the weaker C*sp*^2^-Br bond, substitution in the case of the chloro-iodo derivatives favors cross-coupling through the intrinsically more reactive C*sp*^2^-I bond. The electronic absorption and emission properties of the prepared 2,3-diaryl-6-(phenylethynyl)quinazolines were studied in solvents of different polarity (dichloromethane, toluene, DMF, methanol) and CH_2_Cl_2_-TFA mixture using UV-Vis and emission spectroscopic techniques complemented with density functional theory method to establish the effect of substituents on intramolecular charge transfer properties.

## 1. Introduction

The design and synthesis of polycarbo-substituted quinazolines continue to attract considerable attention in research because of their rich biological activities and interesting photophysical (electronic absorption and emission) properties [1,2,3,4,5,6,7,8]. Halogenated quinazoline moiety has to this end established itself as an important scaffold for transition metal-mediated C*sp*^2^-C*sp*^2^, C*sp*^2^-C*sp* or C*sp*^2^-heteroatom bond formation to afford diversely carbo-(aryl, alkenyl or alkynyl) or heteroatom-substituted quinazolines with potential application in medicinal chemistry [6] and materials [7,8,9]. The versatility of halogenated quinazolines as substrates in these transformations stems from the reactivity of the C*sp*^2^-X bonds, which readily undergo metal exchange or metal-catalyzed cross-coupling to afford novel polysubstituted derivatives [10]. For multihalogenated heterocycles bearing different halogen atoms, selectivity in transition metal-mediated cross-coupling has been established to generally relate to the relative C*sp*^2^-X bond strengths (trend: C-I < C–Br < C-Cl < C-F), which make it possible to effect selective cross-coupling with iodides or bromides in the presence of chlorides [11,12,13]. However, the C(4)-Cl bond of 6-bromo-2,4-dichloroquinazoline [3] and the analogous 2-aryl-6,8-dibromo-4-chloroquinazolines [7] has been found to be more reactive in metal-catalyzed cross-coupling than the weaker C*sp*^2^-Br bond, which in turn undergoes cross-coupling more readily than the C(2)-Cl bond [1,2,3,4,5]. Theoretical calculations at B3LYP level, on the other hand, revealed that the bond dissociation energy of the C(4)-Cl bond (84.8 kcal/mol) for 6-bromo-2,4-dichloroquinazoline is larger than that of the weaker C*sp*^2^-Br bond (83 kcal/mol at B3LYP) [1]. The increased reactivity of the C(4)-Cl bond for quinazolines is attributed to the α-nitrogen effect, which makes this position more electrophilic than the other chlorinated or brominated positions on the heterocyclic scaffold [1,2,4]. Additional activation of the C(4)-Cl position, which also relates to selectivity of cross-coupling in the case of bromo-substituted 4-chloroquinazolines is the consequence of strong PdL_2_ d_xy_ HOMO-heterocycle π* LUMO interaction in the oxidative-addition step which favors C(4)-Cl cross-coupling instead of the weaker C*sp*^2^-Br bond [1,13].

Although there have been numerous reports on the site-selective sequential cross-couplings of the di- and trihalogenated quinazolines, these studies focused mainly on derivatives bearing a combination of chloro-chloro or chloro-bromo as substituents [2,3,7]. The need to access structurally diverse polycarbo-substituted quinazolines prompted us to investigate the relative reactivity of the C(4)-Cl and C*sp*^2^-I bonds of the analogous 2-aryl-4-chloro-6-iodoquinazolines in palladium catalyzed cross-coupling reactions to afford novel polycarbo-substituted quinazolines. Successful discernment of the reactivity of the two C*sp*^2^-halogen bonds led to the first one-pot double cross-coupling (bis-Sonogashira and successive Sonogashira/Stille) approach towards the synthesis of unsymmetrical polycarbo-substituted quinazoline derivatives. Electronic absorption and emission properties of selected examples of the prepared polycarbo-substituted quinazolines were probed in solvents of different polarity (dichloromethane, toluene, DMF, methanol, CH_2_Cl_2_-TFA) using UV-Vis and emission spectroscopic techniques in conjunction with density functional theory (DFT) method to establish the effect of substituents and protonation on intramolecular charge transfer (ICT) properties.

## 2. Results and Discussion

**Scheme 1:**
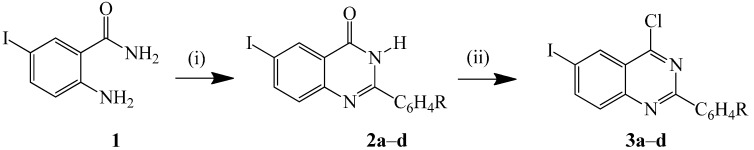
Synthesis and aromatization of the 2-aryl-6-iodoqinazolin-4(3*H*)-ones. *Reagents* *and conditions*: (i) ArCHO, I_2_ (2 equiv.), ethanol, reflux, 7 h; (ii) POCl_3_, NEt_3_, reflux, 6 h.

The 2-aryl-6-iodoquinazolin-4(3*H*)-ones employed as precursors for the synthesis of the requisite 2-aryl-4-chloro-6-iodoquinazolines were, in turn, prepared via cyclocondensation of 2-amino-5-iodobenzamide and benzaldehyde derivatives. 2-Amino-5-iodobenzamide **1** and benzaldehyde derivatives were subjected to molecular iodine in ethanol under reflux for 7 h to afford the corresponding 2-aryl-6-iodoquinazolin-4(3*H*)-ones **2a**–**d** (Scheme 1). The latter were, in turn, subjected to POCl_3_–promoted aromatization in the presence of triethylamine under reflux for 6 h to afford the corresponding 2-aryl-4-chloro-6-iodoquinazolines **3a**–**d**.

**Table:** 

Compound	R	%Yield of 2	%Yield of 3
**a**	4-H	83	86
**b**	4-F	93	78
**c**	4-Cl	89	74
**d**	4-OMe	98	82

**Scheme 2:**
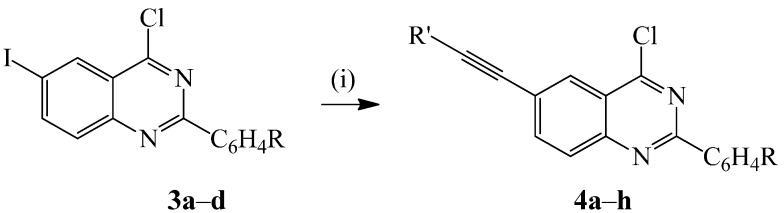
Regioselective C-6 alkynylation of the 2-aryl-4-chloro-6-iodoquinazolines **3a**–**d**. *Reagents and conditions*: (i) R′C≡CH (1.2 equiv.), PdCl_2_(PPh_3_)_2_, CuI, Cs_2_CO_3_, THF, r.t., 18 h.

We explored the reactivity of the dihalogenoquinazolines **3a**–**d** in palladium-copper iodide catalyzed Sonogashira cross-coupling with terminal alkynes as coupling partners. Hitherto, the analogous 2-aryl-6,8-dibromo-4-chloroquinazolines were found to undergo Sonogashira cross-coupling with terminal alkynes at room temperature through C(4)-Cl bond substitution with ease [7]. This literature precedent made it difficult for us at the outset to predict how different the reactivity of the highly activated C*sp*^2^-Cl bond and the intrinsically reactive C*sp*^2^-I bond in **3** would be during Pd-catalyzed C*sp*^2^-C*sp* bond formation. We nevertheless subjected compound **3a** to phenylacetylene (1.2 equiv.) in the presence of dichlorobis(triphenylphosphine)palladium(II) pre-catalyst as a source of active Pd(0) species, CuI as co-catalyst and Cs_2_CO_3_ in THF at r.t. for 18 h in analogy with the literature precedent [7] (Scheme 2). Thin layer chromatography (TLC) analysis of the reaction mixture and the crude product revealed the presence of only two spots, which were purified by column chromatography on silica gel to afford in sequence traces of the undesired dimer and the cross-coupled product characterized using a combination of NMR (^1^H and ^13^C) and IR spectroscopic techniques as the 4-chloro-2-phenyl-6-(2-phenylethynyl)quinazoline **4a**. Its ^13^C-NMR spectrum revealed the absence of the C*sp*^2^-I signal, which resonates at δ *ca*. 93.0 ppm in the spectrum of **3a** and the presence of two carbon signals at δ 88.2, and 92.3 ppm corresponding to the acetylene moiety. The molecular ion region of the mass spectrum of this compound, on the other hand, revealed the presence of the M+ and M+2 peaks in the ratio 3:1 typical for compounds containing the ^35^Cl and ^37^Cl isotopes, thus confirming its 6-alkynyl-4-chloroquinazoline nature. The reaction conditions were then extended to other derivatives **3** using phenylacetylene or 3-butyn-1-ol as coupling partners to afford compounds **4a**–**h**. These results reveal an opposite trend to that observed for the chloro-chloro or chloro-bromo substituted quinazolines in which case, reactivity and selectivity are governed by the electronic position of the C*sp*^2^-X bond (trend: C(4)-Cl > C*sp*^2^-Br > C(2)-Cl > C*sp*^2^-Cl). In order to rationalize the observed selectivity, we probed the relative C*sp*^2^-X bond dissociation energies of **3a** by DFT method [B3LYP/6-311G(d)], which revealed that the C*sp*^2^-I bond is weaker (66.45 kcal/mol) than the C(4)-Cl bond (83.14 kcal/mol). The observed selectivity and reactivity through C*sp*^2^-I bond is thus attributed to the relative C*sp*^2^-X bond strengths, which make it possible to substitute the intrinsically more reactive C*sp*^2^-I bond in the presence of the highly activated C(4)-Cl bond.

**Table:** 

Compound	R	R′	% Yield of 4
**4a**	4-H	-C_6_H_5_	93
**4b**	4-F	-C_6_H_5_	89
**4c**	4-Cl	-C_6_H_5_	78
**4d**	4-OMe	-C_6_H_5_	75
**4e**	4-H	-CH_2_CH_2_OH	90
**4f**	4-F	-CH_2_CH_2_OH	90
**4g**	4-Cl	-CH_2_CH_2_OH	85
**4h**	4-OMe	-CH_2_CH_2_OH	82

**Scheme 3:**
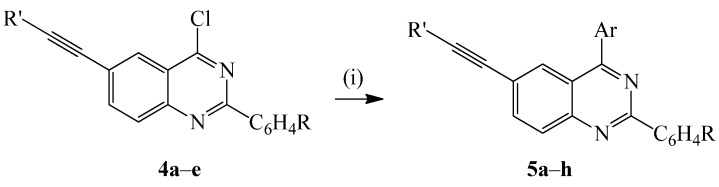
Suzuki-Miyaura cross-coupling of **4a**–**e** with arylboronic acids. *Reagents and conditions*: (i) ArB(OH)_2_, PdCl_2_(PPh_3_)_2_, PCy_3_, K_2_CO_3_, dioxane-water (3:1, *v*/*v*), reflux, 2 h.

The Suzuki-Miyaura cross-coupling of compounds **4a**–**f** with either 4-fluorophenylboronic acid or 4-methoxyphenylboronic acid in the presence of PdCl_2_(PPh_3_)_2_-PCy_3_ catalyst complex and K_2_CO_3_ as a base in DMF under reflux for 2 h afforded the corresponding novel 2,4-diaryl-6-alkynylquinazolines **5a**–**h** (Scheme 3).

**Table:** 

Compound	R	R′	Ar	%Yield of 5
**5a**	4-H	-C_6_H_5_	4-FC_6_H_4_-	76
**5b**	4-F	-C_6_H_5_	4-FC_6_H_4_-	85
**5c**	4-Cl	-C_6_H_5_	4-FC_6_H_4_-	83
**5d**	4-OMe	-C_6_H_5_	4-FC_6_H_4_-	78
**5e**	4-F	-C_6_H_5_	4-MeOC_6_H_4_-	79
**5f**	4-Cl	-C_6_H_5_	4-MeOC_6_H_4_-	70
**5g**	4-OMe	-C_6_H_5_	4-MeOC_6_H_4_-	79
**5h**	4-H	-CH_2_CH_2_OH	4-MeOC_6_H_4_-	90

**Scheme 4:**
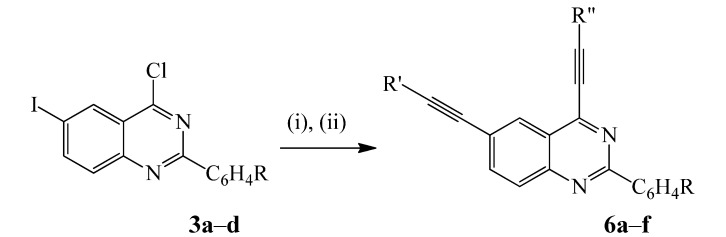
One-pot sequential Sonogashira cross-coupling of **3a**–**d** with terminal alkynes. *Reagents and conditions*: (i) R′C≡CH (1 equiv.), PdCl_2_(PPh_3_)_2_, CuI, Cs_2_CO_3_, THF, r.t., 18 h; (ii) R′′C≡CH (1.2 equiv.), THF, 60 °C, 2 h.

Despite the successes in site-selective metal-catalyzed cross-coupling reactions of the di- or trihalogenated quinazolines bearing different halogen atoms [10], so far there is no literature precedence for their involvement in one-pot sequential multi-step reactions to afford unsymmetrical polycarbo-substituted quinazolines. Since both C(4)-Cl bond [7] and C*sp*^2^-I bond (this investigation) undergo Sonogashira cross-coupling under the same conditions, we opted for the use of a single catalyst mixture on **3a**–**d** and only varied the reaction time and temperature for the subsequent step. The 1st cross-coupling step was carried out with either phenylacetylene or 3-butyn-1-ol (1 equiv.) at r.t. for 18 h as for the synthesis of compounds **4a**–**h** above to avoid double coupling. After 18 h at r.t. (TLC monitoring), the reaction mixtures were each treated with a different alkyne (1.2 equiv.) in THF and then heated at 60 °C to ensure complete conversion of the incipient monoalkynylated product. To our delight, we isolated on silica gel the corresponding unsymmetrically substituted dialkynylated quinazolines **6a**–**h** exclusively after 2 h by column chromatography (Scheme 4).

**Table:** 

Compound	R	R′	R′′	%Yield of 6
**6a**	4-H	-C_6_H_5_	-CH_2_CH_2_OH	78
**6b**	4-F	-C_6_H_5_	-CH_2_CH_2_OH	55
**6c**	4-Cl	-C_6_H_5_	-CH_2_CH_2_OH	65
**6d**	4-OMe	-C_6_H_5_	-CH_2_CH_2_OH	65
**6e**	4-H	-CH_2_CH_2_OH	-C_6_H_5_	61
**6f**	4-F	-CH_2_CH_2_OH	-C_6_H_5_	52
**6g**	4-Cl	-CH_2_CH_2_OH	-C_6_H_5_	75
**6h**	4-OMe	-CH_2_CH_2_OH	-C_6_H_5_	59

**Scheme 5:**
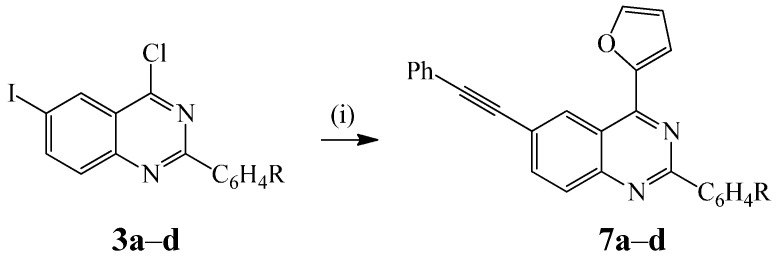
One-pot sequential Sonogashira and Stille cross-coupling of **3a**–**d**. *Reagents and conditions*: (i) R′C≡CH (1 equiv.), PdCl_2_(PPh_3_)_2_, CuI, Cs_2_CO_3_, THF, r.t., 18 h; (ii) 2-(tributylstannyl)furan, THF, 60 °C, 2 h.

The success of the above single-pot bis-Sonogashira cross-coupling prompted us to further investigate the possibility of effecting one-pot successive cross-coupling reactions using different coupling carbon sources. Here we opted for the one-pot sequential Sonogashira and Stille cross-coupling of compounds **3a**–**d** with phenylacetylene and 2-(tributylstannyl)furan. Initial cross-coupling of **3a**–**d** with phenyl acetylene (1 equiv.) in the presence of PdCl_2_(PPh_3_)_2_, CuI, Cs_2_CO_3_, THF at r.t. for 18 h followed by Stille cross-coupling of the incipient 2-aryl-4-chloro-6-(phenylethynyl)quinazoline (tlc monitoring) with 2-(tributylstannyl)furan (1.1 equiv.) in THF and heating at 60 °C afforded albeit in moderate yields products **7a**–**d**, exclusively (Scheme 5).

**Table:** 

Compound	R	%Yield of 7
**7a**	4-H	56
**7b**	4-F	62
**7c**	4-Cl	69
**7d**	4-OMe	59

**Figure 1:**
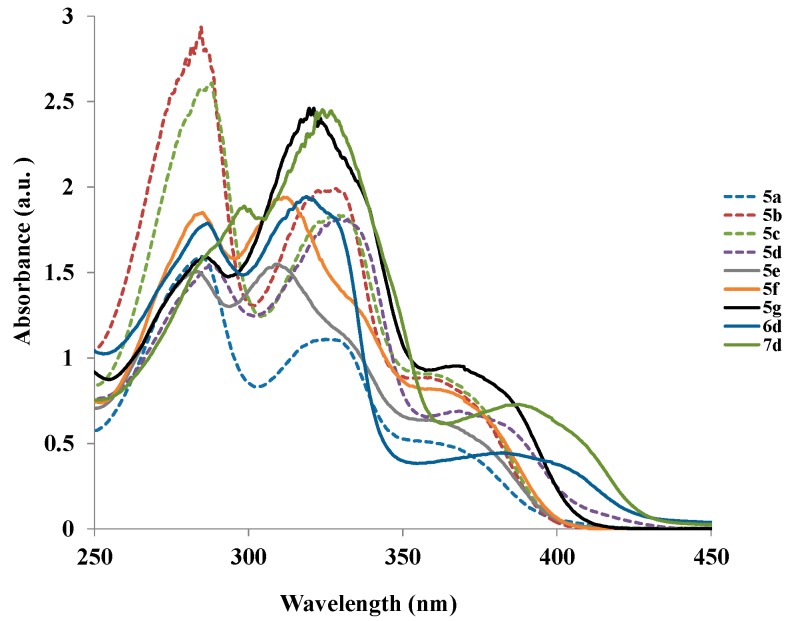
Absorption spectra of **5a**–**g**, **6d** and **7d** in CH_2_Cl_2_ (r.t.) at 2.25 × 10^−5^ mol/L.

The molecular backbone of the compounds prepared in this investigation is interesting because it comprises an electron-deficient quinazoline framework as an electron-acceptor linked to the aryl or heteroaryl ring directly and through a π-conjugated spacer to comprise donor-π-acceptor systems with potential intramolecular charge transfer properties. Moreover, the presence of π-conjugated spacers between the central chromophore and the π-electron donors has been found to extend the conjugated framework to broaden the absorption window and enlarge the Stokes shift characteristics of light emission of a donor-π-acceptor system [14]. As a prelude to compounds with potential photophysical properties, in the last part of this investigation we probed the electronic absorption and emission properties of selected compounds in solution using UV-Vis and fluorescence spectrometry in conjunction with density functional theory (DFT) method. The aim was to establish the effect of aryl and (alkyl/aryl)alkynyl substituents on intramolecular charge transfer (ICT) properties. The UV-Vis spectra of compounds **5a**–**g**, **6d** and **7d** in dichloromethane (CH_2_Cl_2_) reveal the presence of three absorption bands of different intensity (Figure 1). The first two bands in the region λ 283–300 nm and λ 305–330 nm are due to π→π* transition of the conjugated quinazoline ring and the intramolecular donor-acceptor charge transfer absorption, respectively [14]. The less intense broad band in the region λ 358–400 nm presumably corresponds to the weakly allowed π→π* transitions or relatively strong pyrimidine-based n→π* transitions [15]. Both the absorption maxima and the wavelength of these compounds are influenced by the variation of substituents on the heterocyclic framework. The 2-(4-halogenophenyl) substituted derivatives **5b** and **5c**, which bear the moderately resonance-donating 4-(4-fluorophenyl) group exhibit increased intensity of the absorption maxima and slight red shift as compared to **5a** bearing the 2- and 4-phenyl groups. The 2-(4-fluoro/chlorophenyl) groups in compounds **5b** and **5c** presumably withdraw electrons away from the quinazoline moiety causing the moderately resonance-donating 4-fluorophenyl group at the 6-position to increase the π→π* transition, hence the observed increased absorption intensities for these compounds. The absorption maxima for compounds **5d**–**g** bearing the strong resonance donating 4-methoxyphenyl group are located around λ 310–330 nm. Increased peak broadening and reduced intensity for these compounds indicate that the strong electron donating methoxy groups interfere with the conjugation of the π electrons presumably restricting the transition from bonding orbital to the antibonding orbital. Increased broadening and slight red shift are observed for **6d** and **7d** bearing but-3-yn-1-ol or 2-furanyl group at the 4-position.

**Figure 2:**
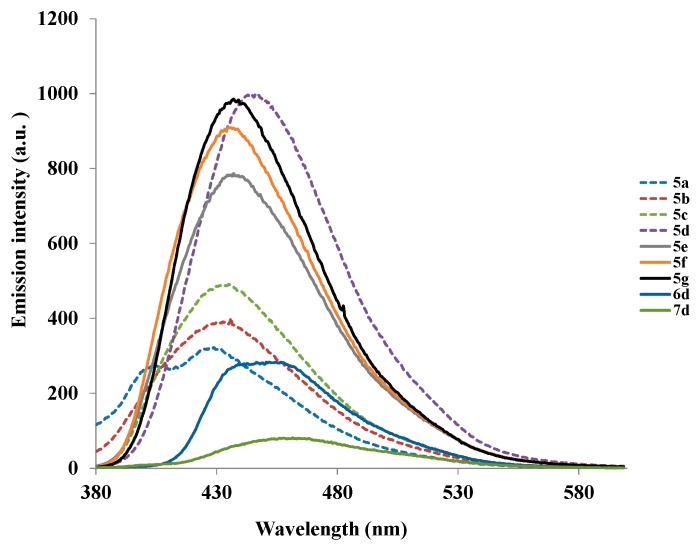
Emission spectra of **5a**–**g**, **6d** and **7d** in CH_2_Cl_2_ (r.t.) at 7.5 × 10^−6^ mol/L (λ_ex_ = 355 nm).

The fluorescence excitation spectra of compounds **5a**–**g**, **6d** and **7d** at room temperature in CH_2_Cl_2_ are characterized by several bands which are located at wavelengths close to those of the absorption spectra. Their emission spectra in CH_2_Cl_2_ at the excitation wavelength, λ_ex_ = 355 nm, reveal similar patterns and are characterized by a single emission band in the region, λ_em_ = 480–495 nm except for **5a** which also showed a shoulder at λ *ca.* 404 nm (Figure 2). These bands are attributed to the π→π* transition resulting from direct π-electron delocalization by the aryl groups and through the conjugate bridge towards the electron-deficient quinazoline ring. The intensity of the emission bands, Stokes shifts, and the fluorescence quantum yields are also influenced by the variation of substituents on the heterocycle (Table 1). The presence of 4-methoxyphenyl group at the 2- and/ or 4-position enhances the emission and the trend in intensity is as follows: **5d** > **5g** > **5f** > **5e**. A combination of the moderately resonance-donating 4-(4-fluorophenyl)- and strongly donating 2-(4-methoxyphenyl)-substituents in **5d** resulted in the highest emission intensity and slight redshift than for **5g** bearing the strongly donating 4-methoxyphenyl groups at the 2- and 4-positions. Compounds **5a**, **5b** and **5c** bearing 4-fluorophenyl ring at the 4-position exhibit relatively reduced emission intensities with peak broadening and the trend in intensity is as follows: **5c** > **5b** > **5a**. Reduced emission intensity and increased broadening are observed for compounds **6d** and **7d** bearing the but-3-yn-1-ol and 2-furanyl groups at the 4-position, respectively. The large Stokes shift values for these compounds are an indication of the high polarizability of the π-conjugated framework.

**Table 1:** The absorption and emission data for compounds **5a**–**g**, **6d** and **7d**.

Compound	λ_max_ abs (nm)	(ε) × 10^3^ mol^−1^·cm^−1^	λ_em_ (nm)	^(a)^ Quantum Yields (Φ)	Stokes Shifts
**5a**	326	49.28	429	0.027	7364
**5b**	328	88.47	436	0.019	7552
**5c**	329	81.55	435	0.025	7406
**5d**	329	80.50	444	0.051	7872
**5e**	309	68.86	437	0.047	9479
**5f**	311	86.24	436	0.043	9218
**5g**	321	109.32	437	0.037	8269
**6d**	318	86.19	454	0.013	9420
**7d**	323	108.98	461	0.003	9268

^(a)^ The relative quantum yields in CH_2_Cl_2_ were calculated according to the equation indicated under Experimental section using quantum yield value of quinine sulfate as the standard (Φ_st_ = 0.55) in 0.50 M H_2_SO_4_.

**Figure 3:**
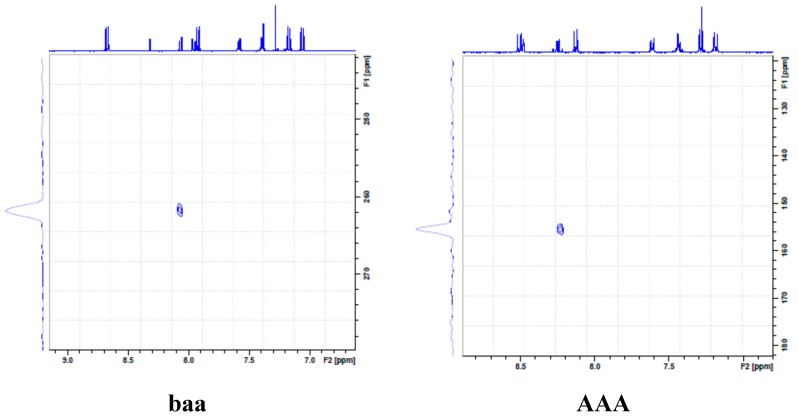
^1^H-^15^N NMR spectra of **5g** in CDCl_3_ (**baa**) and in CDCl_3_+TFA (**AAA**) using nitromethane as an external standard.

**Figure 4:**
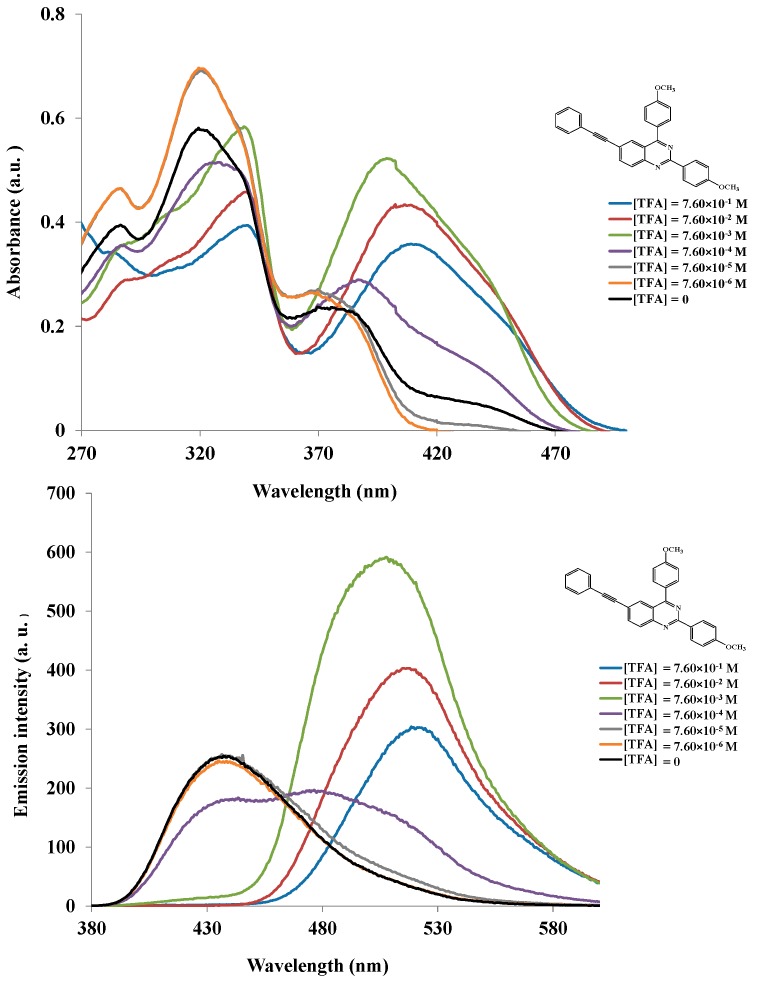
UV-Vis (**upper**) and PL spectra of **5g** in CH_2_Cl_2_ (conc. = 1.46 × 10^−5^ M) at different concentrations of TFA (10^−6^ to 10^−1^).

Achelle *et al.*, previously observed dramatic color change of the analogous arylvinyl-, aryl-, and arylethynyl-substituted quinazoline derivatives upon protonation with trifluoroacetic acid, which became reversible by neutralization with a base (NEt_3_ or *t*-BuOK) [8]. These authors projected that nitrogen at position 3 is the most basic centre for the quinazoline nucleus, however, they were not certain about the exact nitrogen atom (N-1 *vs.* N-2) which is most likely to be protonated or both. Hitherto, Liu *et al.* had also reported a novel approach involving controlled acid protonation of the blue emissive 2,4-diarylquinazoline derivatives, which resulted in white photoluminescence and electroluminescence properties [9]. These authors argued on the basis of X-ray crystal structure that the free space around the N-2 atom is greatly limited by a peripheral phenyl ring to hinder possible protonation by the acid molecules. Acidification of these compounds had maximum impact on the proton NMR chemical shift of H-8 indicating that N-1 is the one that is protonated by trifluoroacetic acid (TFA). [9] Intrigued by these two literature precedents, we decided to probe the optical behavior of compound **5g** in acidic medium. First we acquired the ^1^H-NMR (see Figures S1–S3 in Supplementary Data) and heteronuclear multiple bond correlation (HMBC) ^1^H-^15^N NMR pectra (Figure 3) of **5g** in CDCl_3_ and CDCl_3_+TFA to establish the exact site of protonation. Protonation in CDCl_3_+TFA (3 drops) is confirmed by the presence of a singlet at δ_H_*ca*. 11.5 ppm (see Figure S3 in Supplementary data) and significant downfield shift of the resonance corresponding to H-8 with less effect on the chemical shift of H-7 (Figure 3, **AAA**). Both the ^1^H-^15^N NMR spectra of **5g** in CDCl_3_ and CDCl_3_+TFA (Figure 3) obtained by irradiation of H-8 revealed the presence of a single intense N-15 signal at δ 261.95 ppm before addition of an acid (**baa**) and at δ 155.59 ppm after addition of an acid (**AAA**). The resonance for N-2, on the other hand, was not observed in CDCl_3_ or CDCl_3_+TFA because it is furthest away (five bond distance) from the irradiated H-8 than N-1. The observed significant changes in chemical shifts for H-8 and the nitrogen signal in the ^1^H-^15^N NMR spectrum upon acidification in our view confirm protonation to take place on N-1 in analogy with the previous literature observation by Liu *et al*. [9]. With the exact protonation site for these compounds established, we then probed the electronic absorption and emission properties of **5g** in dichloromethane at different concentrations of trifluoroacetic acid. Addition of TFA is accompanied by color change from green to yellow and an increase in concentration of TFA leads to bathochromic shift of the absorption maxima compared to a neutral solution ([TFA] = 0) of **5g** (Figure 4). The less intense shoulder at λ *ca.* 370 nm in the spectrum of this compound in CH_2_Cl_2_ becomes more intense and broader with increasing acidity due to increased charge transfer from the donors to the protonated quinazoline moiety. Since the π→π* state is much more polarizable than the ground state, a change in polarity of the solvent has been previously found to cause measurable displacements of the π→π* transition towards the red bands [16]. Based on this literature precedent we rationalize the observed increased intensity and bathochromic shift of the absorption maxima and emission maxima (see Figure 4) to be the result of the increased polarity of the medium (CH_2_Cl_2_+TFA) on the highly polar protonated compound. Additional interaction of trifluoroacetic acid with the methoxy groups is expected to occur at lower pH and therefore reduce their propensity for π-electron pair delocalization into the quinazolium moiety. This interaction in turn, would lead to reduced π→π* transition and therefore less pronounced ICT, hence reduced absorption and emission intensities for **5g** at high concentration of trifluoacetic acid (>10^−3^ M). Fluorescence enhancement and absorption changes indicate that this compound is sensitive to pH changes and has potential in pH sensing.

**Figure 5:**
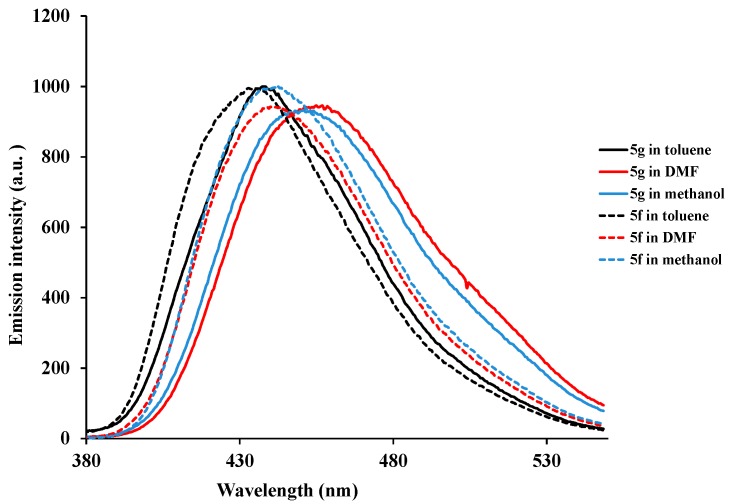
Emission of compounds **5g** and **5f** in solvent of different polarity.

We also studied the emission properties of compounds **5g** (solid lines) and **5f** (broken lines) in solvents of different polarity and hydrogen bonding ability as a means of extending investigation of electronic interactions of these polycarbo-substituted quinazoline scaffold in the excited state (Figure 5). A change in polarity of the medium has been previously found to cause measurable displacements of the π→π* transition towards the red bands [16]. The fluorescence emission spectra of compounds **5g** and **5f** are slightly red-shifted upon increasing the polarity of the solvent, which indicates an increase in dipole moment of excited state compared to ground state. The 2,4-bis(4-methoxyphenyl)–substituted derivative **5g** emits at λ_em_ = 438 nm in non-polar toluene and the emission maxima is red-shift in polar aprotic DMF (λ_em_ = 455 nm) and polar protic methanol (λ_em_ = 453 nm). A slight red-shift is observed in both DMF and methanol for the 2-(4-methoxyphenyl)-substituted derivative **5f** with reduced intensity in DMF. Slight fluorescence quenching in polar protic methanol is observed for **5g** compared to **5f**. This is presumably the consequence of hydrogen bonding with oxygen of the 4-(4-methoxyphenyl) group leading to reduced π→π* transition into the electron poor quinazoline moiety. No appreciable difference in fluorescence intensity was observed for compound **5f** in toluene and methanol.

**Figure 6:**
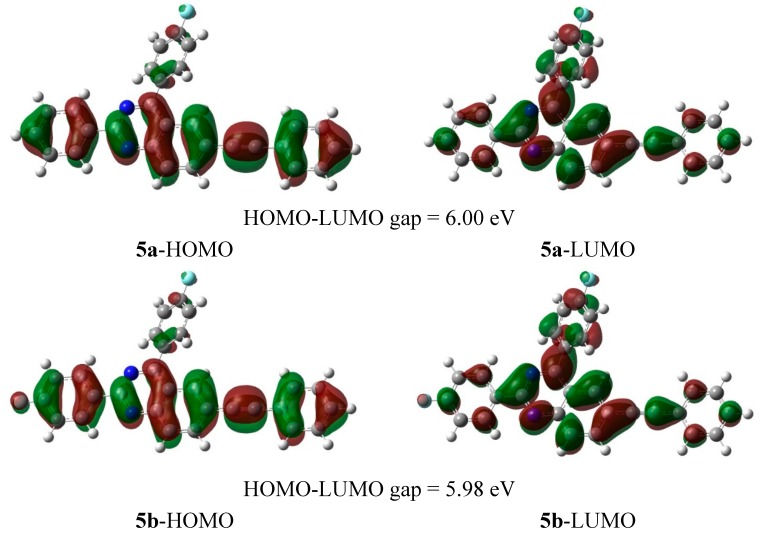

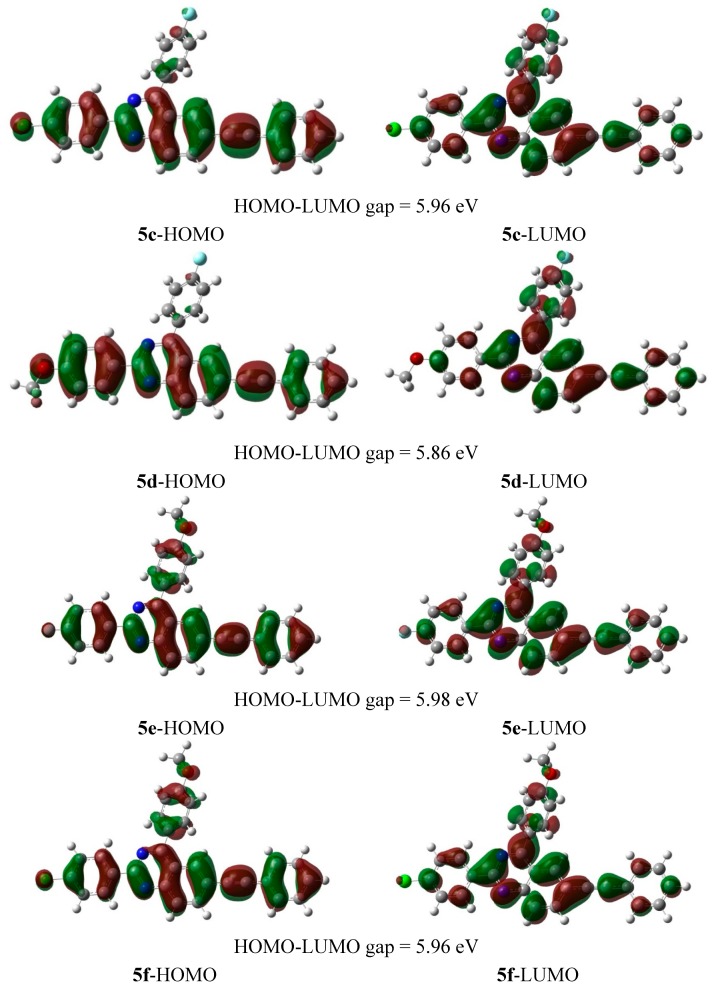

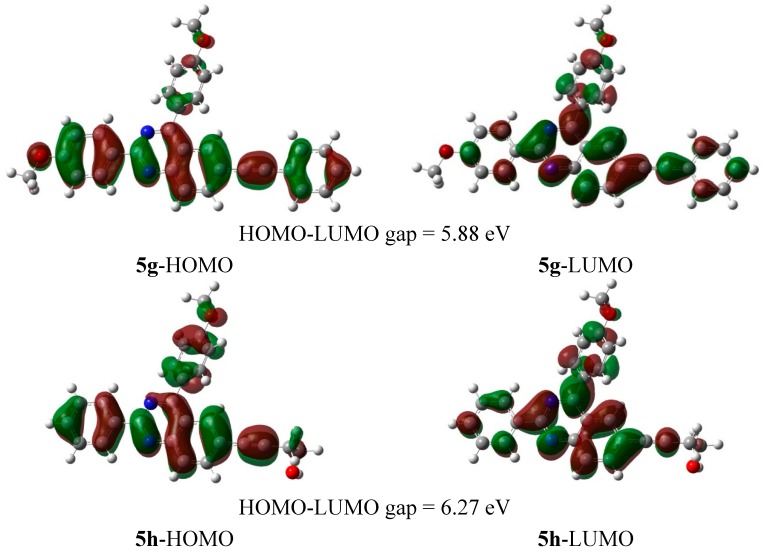
HOMO and LUMO surfaces of compounds **5a**–**h** in CH_2_Cl_2_ [CAM-B3LYP/6-31G(d,p), isovalue = 0.02 and density = 0.0004].

The solvent polarity dependent electronic transitions may result from dipolar interaction with DMF and methanol thus suggesting the intramolecular charge transfer (ICT) of the emission state in which the HOMOs and LUMOs are presumably localized on the aryl rings and the quinazoline-based moiety, respectively. To prove this hypothesis, we employed computational methods to determine the HOMO and LUMO orbital energies of compounds **5a**–**h**. The HOMO and LUMO surfaces of compounds **5a**–**h** in CH_2_Cl_2_ are illustrated in Figure 6 and the HOMO-LUMO gap for all the compounds is about 6 eV. In general, for the compounds, there are no significant changes observed on the electron density distributions. The HOMO is localized over the entire molecule while the LUMO tends to be localized over the quinazoline moiety representing the π and π* orbitals, respectively.

**Table:** 

Compound	λ_em_ (nm) CH_2_Cl_2_	λ_em_ (nm) Toluene	λ_em_ (nm) DMF	λ_em_ (nm) CH_3_OH
**5f**	436	436	440	442
**5g**	437	438	455	453

## 3. Experimental Section

### 3.1. General

Melting points were recorded on a Thermocouple digital melting point apparatus and are uncorrected. IR spectra were recorded as powders using a Bruker VERTEX 70 FT-IR Spectrometer (Bruker Optics, Billerica, MA, USA) with a diamond ATR (attenuated total reflectance) accessory by using the thin-film method. For column chromatography, Merck kieselgel 60 (0.063–0.200 mm) (Merck KGaA, Frankfurt, Germany) was used as stationary phase. NMR spectra were obtained as CDCl_3_ or DMSO-*d*_6_ solutions using Agilent 500 MHz NMR spectrometer (Agilent Technologies, Oxford, UK) and the chemical shifts are quoted relative to the TMS peak. Low- and high-resolution mass spectra were recorded at an ionization potential of 70 eV using Synapt G2 Quadrupole Time-of-flight mass spectrometer (Waters Corp., Milford, MA, USA) at the University of Stellenbosch Mass Spectrometry Unit. The UV-Vis spectra were recorded on a Cecil (Cecil Instruments, Cambridge, UK) CE 9500 (9000 Series) UV-Vis spectrometer while emission spectra were taken using a Perkin Elmer LS45 fluorescence spectrometer (Perkin Elmer, Llantrisant, UK). The quantum efficiencies of fluorescence (Φ_fl_) were obtained with the following equation:

Φ_x_ = Φ_st_ × (*F_x_*/*F_st_*) × (A_st_/A_x_) × (*n*_x_^2^/*n*_st_^2^) (1)

*F* denotes the area under the fluorescence band (*F* = ^a^*I*_fl_(λ), where *I*_fl_(λ) is the fluorescence intensity at each emission wavelength), A denotes the absorbance at the excitation wavelength, and *n* is the refractive index of the solvent [17].

### 3.2. Synthesis of 2-Amino-5-iodobenzamide *(**1**)*

A stirred suspension of anthranilamide (1.00 g, 7.34 mmol) in acetonitrile (20 mL) was treated with *N*-iodosuccinimide (1.65 g, 7.34 mmol) at room temperature. The mixture was stirred at this temperature for 30 min. and then quenched with an ice-cold saturated aqueous solution of sodium thiosulphate. The resultant precipitate was filtered and washed thoroughly with cold water and then recrystallized from ethanol to afford **1** as a white solid (1.69 g, 88%); mp. 198–200 °C (Lit. [18] 197–198 °C); ν_max_ (ATR) 502, 531, 664, 817, 1062, 1228, 1384, 1600, 3162, 3278, 3343 cm^−1^; δ_H_ (500 MHz, DMSO-*d*_6_) 6.53 (d, *J* = 8.5 Hz, 1H), 6.69 (s, 2H), 7.12 (br s, 1H), 7.36 (dd, *J* = 2.0 and 8.5 Hz, 1H), 7.80 (d, *J* = 2.0 Hz, 1H), 7.81 (br s, 1H); δ_C_ (150 MHz, DMSO-*d*_6_) 74.8, 116.5, 119.4, 136.9, 140.2, 150.1, 170.3.

### 3.3. Typical Procedure for the Synthesis of the 2-Aryl-6-iodoquinazolin-4(3H)-ones ***2a**–**2d***

#### 3.3.1. 6-Iodo-2-phenylquinazolin-4(3*H*)-one (**2a**)

A stirred mixture of **1** (1.00 g, 3.81 mmol), benzaldehyde (0.48 g, 4.57 mmol) and iodine (1.93 g, 7.62 mmol) in ethanol (100 mL) was heated under reflux for 8 h and then allowed to cool to room temperature. An ice-cold saturated aqueous solution of sodium thiosulphate was added to the mixture and the resultant precipitate was filtered and washed thoroughly with cold water. The product was recrystallized to afford **2a** as a white solid (1.11 g, 83%), mp. >345 °C (acetonitrile); ν_max_ (ATR) 542, 624, 698, 777, 834, 1284, 1460, 1562, 1595, 1667, 2853, 2955 cm^−1^; δ_H_ (500 MHz, DMSO-*d*_6_) 7.52 (d, *J* = 8.5 Hz, 1H), 7.54 (t, *J* = 7.5 Hz, 2H), 7.59 (d, *J* = Hz, 1H), 8.11 (dd, *J* = 2.0 and 8.5 Hz, 1H), 8.17 (d, *J* = 7.0 Hz, 2H), 8.41 (d, *J* = 2.0 Hz, 1H), 12.69 (brs, 1H); δ_C_ (125 MHz, DMSO-*d*_6_) 91.3, 123.4, 128.3, 128.9, 130.0, 131.8, 133.8, 134.7, 142.9, 148.8, 154.4, 162.5; *m*/*z* 349 (100, MH^+^); HRMS (ES): MH^+^, found 348.9833. C_14_H_10_IN_2_O^+^ requires 348.9760.

#### 3.3.2. 2-(4-Fluorophenyl)-6-iodoquinazolin-4(3*H*)-one (**2b**)

A mixture of **1** (1.00 g, 3.81 mmol), 4-fluorobenzaldehyde (0.57 g, 4.57 mmol) and iodine (1.93 g, 7.62 mmol) in ethanol (100 mL) afforded **2b** as a white solid (1.30 g, 93%), mp. 337–339 °C (acetonitrile); ν_max_ (ATR) 538, 648, 737, 830, 840, 942, 1154, 1460, 1514, 1572, 1667, 2867, 2926 cm^−1^; δ_H_ (500 MHz, DMSO-*d*_6_) 7.39 (t, *J* = 9.0 Hz, 2H), 7.51 (d, *J* = 9.0 Hz, 1H), 8.11 (dd, *J* = 2.0 and 8.5 Hz, 1H), 8.24 (t, *J* = 9.0 Hz. 2H), 8.40 (d, *J* = 2.0 Hz, 1H), 12.72 (s, 1H); δ_C_ (125 MHz, DMSO-*d*_6_) 91.9, 116.1 (d, ^2^*J*_CF_ = 21.8 Hz), 123.1, 129.5, 130.0 (d, ^2^*J*_CF_ = 3.7 Hz), 130.9 (d, ^3^*J*_CF_ = 8.5 Hz) 134.6, 143.3, 148.3, 152.5, 161.4, 164.6 (d, ^1^*J*_CF_ = 250.5 Hz); *m*/*z* 367 (100, MH^+^); HRMS (ES): MH^+^, found 366.9744. C_14_H_9_FIN_2_O^+^ requires 366.9747.

#### 3.3.3. 2-(4-Chlorophenyl)-6-iodoquinazolin-4(3*H*)-one (**2c**)

A mixture of **1** (1.00 g, 3.81 mmol), 4-chlorobenzaldehyde (0.64 g, 4.57 mmol) and iodine (1.93 g, 7.62 mmol) in ethanol (100 mL) afforded **2c** as a white solid (1.31 g, 89%), mp. >345 °C (acetonitrile); ν_max_ (ATR) 541, 622, 644, 730, 830, 841, 940, 1091, 1459, 1473, 1556, 1595, 1667, 2922, 3038 cm^−1^; δ_H_ (500 MHz, DMSO-*d*_6_) 7.51 (d, *J* = 8.5 Hz, 1H), 7.61 (d, *J* = 8.5 Hz, 2H), 8.10 (dd, *J* = 2.0 and 8.5 Hz, 1H), 8.19 (d, *J* = 8.5 Hz, 2H), 8.40 (d, *J* = 2.0 Hz, 1H), 12.74 (br s, 1H); δ_C_ (125 MHz, DMSO-*d*_6_) 92.0, 123.3, 129.1, 130.0, 130.2, 132.1, 134.7, 136.8, 143.3, 148.4, 152.8, 161.0; *m*/*z* 383 (100, MH^+^); HRMS (ES): MH^+^, found 382.9448. C_14_H_9_^35^ClIN_2_O ^+^ requires 382.9370.

#### 3.3.4. 6*-*Iodo-2-(4-methoxyphenyl)quinazolin-4(3*H*)-one (**2d**)

A mixture of **1** (1.00 g, 3.81 mmol), 4-methoxybenzaldehyde (0.62 g, 4.57 mmol) and iodine (1.93 g, 7.62 mmol) in ethanol (20 mL) afforded **2d** as a white solid (1.42 g, 98%), mp. 321–323 °C (acetonitrile); ν_max_ (ATR) 541, 649, 742, 831, 841, 939, 1027, 1250, 1459, 1517, 1594, 1662, 2852, 2922 cm^−1^; δ_H_ (500 MHz, DMSO-*d*_6_) 3.84 (s, 3H), 7.07 (d, *J* = 9.0 Hz, 2H), 7.47 (d, *J* = 9.0 Hz, 1H), 8.06 (dd, *J* = 2.0 and 8.5 Hz, 1H), 8.17 (d, *J* = 8.5 Hz, 2H), 8.37 (d, *J* = 2.0 Hz, 1H), 12.50 (br s, 1H); δ_C_ (125 MHz, DMSO-*d*_6_) 55.9, 91.1, 114.5, 123.0, 125.3, 129.8, 130.0, 134.6, 143.1, 148.7, 153.3, 161.8, 162.4; *m*/*z* 379 (100, MH^+^); HRMS (ES): MH^+^, found 378.9943. C_15_H_12_IN_2_O_2_^+^ requires 378.9865.

### 3.4. Typical Procedure for the Synthesis of the 2-Aryl-4-chloro-6-iodoquinazolines ***3a**–**d***

#### 3.4.1. 4-Chloro-6-iodo-2-phenylquinazoline (**3a**)

A stirred mixture of **2a** (1.00 g, 2.87 mmol) and phosphoryl chloride (10 mL) was treated dropwise with triethylamine (4 mL) at room temperature. The mixture was then heated under reflux for 6 h and allowed to cool to room temperature. An ice-cold water was added to the mixture and the aqueous layer was extracted with chloroform. The combined organic layers were washed with water and then dried over anhydrous MgSO_4_, filtered and evaporated under reduced pressure to afford **3a** as a brown solid (0.91 g, 86%), mp. 148–150 °C (ethanol); ν_max_ (ATR) 532, 663, 685, 703, 830, 866, 985, 1295, 1410, 1469, 1535, 1555 cm^−1^; δ_H_ (500 MHz, CDCl_3_) 7.52–7.55 (m, 3H), 7.81 (d, *J* = 8.5 Hz, 1H), 8.17 (dd, *J* = 1.5 and 8.5 Hz, 1H), 8.57–8.59 (m, 2H), 8.63 (d, *J* = 1.5 Hz, 1H); δ_C_ (125 MHz, CDCl_3_) 93.3, 123.8, 128.7, 128.8, 130.3, 131.4, 134.6, 136.2, 143.6, 150.8, 160.4, 160.9; *m*/*z* 367 (100, MH^+^); HRMS (ES): MH^+^, found 366.9498. C_14_H_9_^35^ClIN_2_^+^ requires 366.9499.

#### 3.4.2. 4-Chloro-2-(4-fluorophenyl)-6-iodoquinazoline (**3b**)

A mixture of **2b** (1.00 g, 2.60 mmol), phosphoryl chloride (10 mL) and NEt_3_ (4 mL) afforded **3b** as a white solid (1.00 g, 78%), mp. 192–193 °C (ethanol); ν_max_ (ATR) 548, 648, 687, 736, 826, 845, 986, 1156, 1214, 1298, 1343, 1410, 1553, 1595 cm^−1^; δ_H_ (500 MHz, CDCl_3_) 7.21 (t, *J* = 9.0 Hz, 2H), 7.83 (d, *J* = 9.0 Hz, 1H), 8.17 (dd, *J* = 2.0 and 8.5 Hz, 1H), 8.59 (t, *J* = 9.0 Hz, 2H), 8.63 (d, *J* = 2.0 Hz, 1H); δ_C_ (125 MHz, CDCl_3_) 93.3, 115.7 (d, ^2^*J*_CF_ = 21.8 Hz), 123.7, 130.2, 131.0 (d, ^3^*J*_CF_ = 8.5 Hz), 132.5 (d, ^4^*J*_CF_ = 3.7 Hz), 134.6, 143.7, 150.8, 159.4, 160.9, 165.1 (d, ^1^*J*_CF_ = 250.2 Hz); *m*/*z* 385 (100, MH^+^); HRMS (ES): MH^+^, found 384.9398. C_14_H_8_^35^ClFIN_2_^+^ requires 384.9405.

#### 3.4.3. 4-Chloro-2-(4-chlorophenyl)-6-iodoquinazoline (**3c**)

A mixture of **2c** (1.00 g, 2.61 mmol), phosphoryl chloride (10 mL) and NEt_3_ (4 mL) afforded **3c** as a white solid (0.78 g, 74.5%), mp. 207–209 °C (ethanol); ν_max_ (ATR) 533, 732, 829, 878, 1088, 1295, 1343, 1411, 1534, 1552, 1592 cm^−1^; δ_H_ (500 MHz, CDCl_3_) 7.50 (d, *J* = 8.5 Hz, 2H), 7.80 (d, *J* = 9.0 Hz, 1H), 8.18 (dd, *J* = 2.0 and 8.5 Hz, 1H), 8.53 (d, *J* = 9.0 Hz, 2H), 8.63 (d, *J* = 2.0 Hz, 1H); δ_C_ (125 MHz, CDCl_3_) 93.6, 123.8, 128.9, 130.1, 130.3, 134.6, 134.7, 137.7, 143.8, 150.8, 159.4, 161.0; *m*/*z* 401 (100, MH^+^); HRMS (ES): MH^+^, found 400.9106. C_14_H_8_^35^Cl_2_IN_2_^+^ requires 400.9109. 

#### 3.4.4. 4-Chloro-6-iodo-2-(4-methoxyphenyl)quinazoline (**3d**)

A mixture of **2d** (1.00 g, 2.64 mmol), phosphoryl chloride (10 mL) and NEt_3_ (4 mL) afforded **3d** as a yellow solid (0.87 g, 82%), mp. 165–167 °C (ethanol); ν_max_ (ATR) 523, 562, 647, 736, 783, 828, 844, 870, 984, 1026, 1166, 1295, 1411, 1466, 1553, 1595 cm^−1^; δ_H_ (500 MHz, CDCl_3_) 3.91 (s, 3H), 7.03 (d, *J* = 9.0 Hz, 2H), 7.76 (d, *J* = 8.5 Hz, 1H), 8.13 (dd, *J* = 2.0 and 8.5 Hz, 1H), 8.53 (d, *J* = 9.0 Hz, 2H), 8.59 (d, *J* = 2.0 Hz, 1H); δ_C_ (125 MHz, CDCl_3_) 55.4, 92.5, 114.0, 123.4, 128.9, 130.1, 130.5, 134.5, 143.4, 151.0, 160.2, 160.7, 162.4; *m*/*z* 397 (100, MH^+^); HRMS (ES): MH^+^, found 396.9691. C_15_H_11_^35^ClIN_2_O^+^ requires 396.9605.

### 3.5. Typical Procedure for Site-Selective Sonogashira Cross-Coupling of ***3a**–**d*** with Terminal Alkynes

#### 3.5.1. 4-Chloro-2-phenyl-6-(phenylethynyl)quinazoline (**4a**)

A stirred mixture of **3a** (0.30 g, 0.82 mmol), PdCl_2_(PPh_3_)_2_ (0.057 g, 0.082 mmol), CuI (0.008 g, 0.041 mmol) and Cs_2_CO_3_ (0.40 g, 1.23 mmol) in THF (15 mL) was purged with argon gas for 30 min. Phenylacetylene (0.10 g, 0.98 mmol) was added to the mixture using a syringe. The reaction mixture was stirred at room temperature for 24 h and then quenched with an ice-cold water. The product was extracted into chloroform and the combined organic layers were washed with water, dried over Na_2_SO_4_, filtered and evaporated under reduced pressure. The residue was recrystallized to afford **4a** as a yellow solid (0.26 g, 93%), R*_f_* (2:1 hexane/toluene) 0.36, mp. 164–165 °C; ν_max_ (ATR) 526, 649, 686, 706, 754, 848, 1327, 1361, 1413, 1556, 2208 cm^−1^; δ_H_ (500 MHz, CDCl_3_) 7.40–7.41 (m, 3H), 7.53–7.54 (m, 3H), 7.60–7.65 (m, 2H), 8.01 (dd, *J* = 1.5 and 9.0 Hz, 1H), 8.06 (d, *J* = 9.0 Hz, 1H), 8.41 (d, *J* = 1.5 Hz, 1H), 8.59–7.70 (m, 2H); δ_C_ (125 MHz, CDCl_3_) 88.2, 92.3, 122.4, 122.5, 123.5, 128.5, 128.6, 128.7, 128.8, 128.9, 129.0, 131.4, 131.8, 136.5, 137.4, 151.2, 160.5, 162.0; *m*/*z* 341 (100, MH^+^); HRMS (ES): MH^+^, found 341.0838. C_22_H_14_^35^ClN_2_^+^ requires 341.0846.

#### 3.5.2. 4-Chloro-2-(4-fluorophenyl)-6-(phenylethynyl)quinazoline (**4b**)

A mixture of **3b** (0.30 g, 0.78 mmol), PdCl_2_(PPh_3_)_2_(0.054 g, 0.078 mmol), CuI (0.007 g, 0.039 mmol), Cs_2_CO_3_ (0.38 g, 1.17 mmol) and phenylacetylene (0.096 g, 0.94 mmol) in THF (15 mL) afforded **4b** as a yellow solid (0.25 g, 89%), R*_f_* (2:1 hexane/toluene) 0.47, mp. 189–191 °C; ν_max_ (ATR) 524, 600, 682, 751, 841, 995, 1160, 1222, 1366, 1414, 1564, 2214 cm^−1^; δ_H_ (500 MHz, CDCl_3_) 7.21 (t, *J* = 8.5 Hz, 2H), 7.39–7.41 (m, 3H), 7.60–7.62 (m, 2H), 8.01 (dd, *J* = 1.5 and 8.5 Hz, 1H), 8.03 (d, *J* = 8.5 Hz, 1H), 8.40 (d, *J* = 1.5 Hz, 1H), 8.61 (t, *J* = 8.5 Hz, 2H); δ_C_ (125 MHz, CDCl_3_) 88.2, 92.4, 115.7 (d, ^2^*J*_CF_ = 21.7 Hz), 122.3, 122.4, 123.5, 128.5, 128.8, 128.9, 129.0, 131.0 (d, ^3^*J*_CF_ = 8.5 Hz), 131.8, 132.6 (d, ^4^*J*_CF_ = 3.8 Hz), 137.6, 151.2, 159.5, 162.1, 165.1 (d, ^1^*J*_CF_ = 250.2 Hz); *m*/*z* 359 (100, MH^+^); HRMS (ES): MH^+^, found 359.0751. C_22_H_13_^35^ClFN_2_^+^ requires 359.0751.

#### 3.5.3. 4-Chloro-2-(4-chlorophenyl)-6-(phenylethynyl)quinazoline (**4c**)

A mixture of **3c** (0.30 g, 0.75 mmol), PdCl_2_(PPh_3_)_2_(0.052 g, 0.074 mmol), CuI (0.007 g, 0.037 mmol), Cs_2_CO_3_ (0.36 g, 1.12 mmol) and phenylacetylene (0.091 g, 0.90 mmol) in THF (15 mL) afforded **4c** as a yellow solid (0.22 g, 78%), R*_f_* (2:1 hexane/toluene) 0.56, mp. 214–215 °C; ν_max_ (ATR) 523, 680, 747, 836, 1088, 1364, 1404, 1417, 1538, 1552, 2211; cm^−1^; δ_H_ (500 MHz, CDCl_3_) 7.41–7.42 (m, 3H), 7.51 (d, *J* = 9.0 Hz, 2H), 7.60–7.62 (m, 2H), 8.03 (dd, *J* = 1.5 and 8.5 Hz, 1H), 8.07 (d, *J* = 8.5 Hz, 1H), 8.42 (d, *J* = 1.5 Hz, 1H), 8.56 (d, *J* = 9.0 Hz, 2H); δ_C_ (125 MHz, CDCl_3_) 88.1, 92.5, 122.4, 122.4, 123.7, 128.5, 128.7, 128.9, 129.0, 129.1, 130.1, 131.8, 134.9, 137.6, 137.7, 151.1, 159.4, 162.1; *m*/*z* 375 (100, MH^+^); HRMS (ES): MH^+^, found 375.0468. C_22_H_13_^35^Cl_2_N_2_^+^ requires 375.0456.

#### 3.5.4. 4-Chloro-2-(4-methoxyphenyl)-6-(phenylethynyl)quinazoline (**4d**)

A mixture of **3d** (0.30 g, 0.75 mmol), PdCl_2_(PPh_3_)_2_ (0.053 g, 0.075 mmol), CuI (0.007 g, 0.037 mmol), Cs_2_CO_3_ (0.36 g, 1.12 mmol) and phenylacetylene (0.091 g, 0.90 mmol) in THF (15 mL) afforded **4d** as a yellow solid (0.21 g, 75%), R*_f_* (1:1 hexane/toluene) 0.50, mp. 170–172 °C; ν_max_ (ATR) 526, 564, 606, 747, 789, 831, 849, 995, 1028, 1173, 1246, 1364, 1413, 1553, 2209 cm^−1^; δ_H_ (500 MHz, CDCl_3_) 3.90 (s, 3H), 7.03 (d, *J* = 8.5 Hz, 2H), 7.40–7.41 (m, 3H), 7.60 (d, *J* = 3.5 Hz, 2H), 7.96 (dd, *J* = 1.5 and 8.5 Hz, 1H), 7.99 (d, *J* = 8.5 Hz, 1H), 8.36 (d, *J* = 1.5 Hz, 1H), 8.54 (d, *J* = 8.5 Hz, 2H); δ_C_ (125 MHz, CDCl_3_) 55.4, 88.3, 92.0, 114.0, 122.0, 122.6, 122.8, 128.5, 128.7, 128.8, 128.9, 129.1, 130.6, 131.8, 137.3, 151.4, 160.3, 161.8, 162.4; *m*/*z* 371 (100, MH^+^); HRMS (ES): MH^+^, found 371.0943. C_23_H_16_^35^ClN_2_O^+^ requires 341.0951.

#### 3.5.5. 4-(4-Chloro-2-phenylquinazolin-6-yl)but-3-yn-1-ol (**4e**)

A mixture of **3a** (0.30 g, 0.82 mmol), PdCl_2_(PPh_3_)_2_ (0.057g, 0.082 mmol), CuI (0.008 g, 0.041 mmol), Cs_2_CO_3_ (0.40 g, 1.23 mmol) and 3-butyn-1-ol (0.068 g, 0.98 mmol) in THF (20 mL) afforded **4e** as a yellow solid (0.23 g, 90%), R*_f_* (2:1 hexane/ethyl acetate) 0.37, mp. 139–141 °C; ν_max_ (ATR) 554, 687, 705, 767, 836, 991, 1062, 1211, 1306, 1361, 1417, 1557, 2235, 3218 cm^−1^; δ_H_ (500 MHz, CDCl_3_) 1.91 (s, 1H), 2.78 (t, *J* = 6.5 Hz, 2H), 3.90 (dt, *J* = 6.5 and 8.5 Hz, 2H), 7.52–7.54 (m, 3H), 7.88 (dd, *J* = 1.5 and 8.5 Hz, 1H), 8.00 (d, *J* = 8.5 Hz, 1H), 8.28 (d, *J* = 1.5 Hz, 1H), 8.57–8.59 (m, 2H); δ_C_ (125 MHz, CDCl_3_) 23.9, 61.0, 81.3, 89.9, 122.3, 123.6, 128.6, 128.7, 128.8, 128.9, 131.3, 136.5, 137.6, 151.1, 160.4, 161.9; *m*/*z* 309 (100, MH^+^); HRMS (ES): MH^+^, found 309.0789. C_18_H_14_^35^ClN_2_O^+^ requires 309.0795.

#### 3.5.6. 4-(4-Chloro-2-(4-fluorophenyl)quinazolin-6-yl)but-3-yn-1-ol (**4f**)

A mixture of **3b** (0.30 g, 0.78 mmol), PdCl_2_(PPh_3_)_2_(0.054 g, 0.078 mmol), CuI (0.007 g, 0.039 mmol), Cs_2_CO_3_ (0.38 g, 1.17 mmol) and 3-butyn-1-ol (0.066 g, 0.94 mmol) in THF (15 mL) afforded **4f** as a yellow solid (0.23 g, 90%), R*_f_* (2:1 hexane/ethyl acetate) 0.39, mp. 180–181 °C; ν_max_ (ATR) 557, 647, 740, 748, 840, 986, 1064, 1150, 1221, 1307, 1361, 1410, 1557, 2235, 3196 cm^−1^; δ_H_ (500 MHz, CDCl_3_) 1.84 (br s, 1H), 2.79 (t, *J* = 6.5 Hz, 2H), 3.90 (t, *J* = 6.5 Hz, 2H), 7.20 (t, *J* = 9.0 Hz, 2H), 7.90 (dd, *J* = 1.5 and 8.5 Hz, 1H), 7.98 (*J* = 8.5 Hz, 1H), 8.29 (d, *J* = 1.5 Hz, 1H), 8.60 (t, *J* = 9.0 Hz, 2H); δ_C_ (125 MHz, CDCl_3_) 23.9, 61.0, 81.2, 89.9, 115.7 (d, ^2^*J*_CF_ = 20.8 Hz), 122.1, 123.5, 128.8, 128.9, 130.9 (d, ^3^*J*_CF_ = 8.5 Hz), 132.6 (d, ^4^*J*_CF_ = 3.7 Hz), 137.7, 151.0, 159.3, 161.9, 165.0 (d, ^1^*J*_CF_ = 250.3 Hz); *m*/*z* 327 (100, MH^+^); HRMS (ES): MH^+^, found 327.0694. C_18_H_13_^35^ClFN_2_O^+^ requires 327.0700.

#### 3.5.7. 4-(4-Chloro-2-(4-chlorophenyl)quinazolin-6-yl)but-3-yn-1-ol (**4g**)

A mixture of **3c** (0.30 g, 0.75 mmol), PdCl_2_(PPh_3_)_2_(0.052 g, 0.075 mmol), CuI (0.007 g, 0.037 mmol), Cs_2_CO_3_ (0.36 g, 1.12 mmol) and 3-butyn-1-ol (0.062 g, 0.89 mmol) in THF (15 mL) afforded **4g** as a yellow solid (0.22 g, 85%), R*_f_* (2:1 hexane/ethyl acetate) 0.40, mp. 199–201 °C; ν_max_ (ATR) 556, 647, 701, 735, 840, 991, 1011, 1064, 1091, 1360, 1403, 1418, 1539, 2233, 3213 cm^−1^; δ_H_ (500 MHz, CDCl_3_) 1.84 (t, *J* = 6.0 Hz, 1H), 2.79 (t, *J* = 6.5 Hz, 2H), 3.90 (q, *J* = 6.0 Hz, 2H), 7.50 (d, *J* = 8.0 Hz, 2H), 7.90 (dd, *J* = 1.5 and 8.5 Hz, 1H), 7.99 (*J* = 8.5 Hz, 1H), 8.30 (d, *J* = 1.5 Hz, 1H), 8.53 (d, *J* = 8.5 Hz, 2H); δ_C_ (125 MHz, CDCl_3_) 23.9, 61.0, 81.3, 90.0, 115.6, 115.8, 122.2, 123.6, 128.8, 130.9, 131.0, 132.6, 137.7, 151.0, 159.4, 162.0; *m*/*z* 343 (100, MH^+^); HRMS (ES): MH^+^, found 343.0402. C_18_H_13_^35^Cl_2_N_2_O^+^ requires 343.0405.

#### 3.5.8. 4-(4-Chloro-2-(4-methoxyphenyl)quinazolin-6-yl)but-3-yn-1-ol (**4h**)

A mixture of **3d** (0.30 g, 0.75 mmol), PdCl_2_(PPh_3_)_2_(0.053 g, 0.075 mmol), CuI (0.007 g, 0.037 mmol), Cs_2_CO_3_ (0.36 g, 1.12 mmol) and 3-butyn-1-ol (0.063 g, 0.90 mmol) in THF (15 mL) afforded **4h** as a yellow solid (0.21 g, 82%), R*_f_* (2:1 hexane/ethyl acetate) 0.37, mp. 159–160 °C; ν_max_ (ATR) 596, 744, 789, 836, 850, 988, 1033, 1061, 1163, 1173, 1247, 1311, 1361, 1416, 1551, 2230, 3285 cm^−1^; δ_H_ (500 MHz, CDCl_3_) 1.85 (t, *J* = 6.0 Hz, 1H), 2.78 (t, *J* = 6.5, 2H), 3.89 (t, *J* = 6.5 Hz, 2H), 3.91 (s, 3H), 7.03 (d, *J* = 9.5 Hz, 2H), 7.86 (dd, *J* = 1.5 and 8.5 Hz, 1H), 7.95 (d, *J* = 8.5 Hz, 1H), 8.27 (d, *J* = 1.5 Hz, 1H), 8.54 (d, *J* = 9.0 Hz, 2H); δ_C_ (125 MHz, CDCl_3_) 23.9, 55.4, 61.1, 81.4, 89.5, 114.1, 121.9, 122.9, 128.7, 128.8, 129.1, 130.5, 137.6, 151.2, 160.2, 161.8, 162.4; *m*/*z* 339 (100, MH^+^); HRMS (ES): MH^+^, found 339.0894. C_19_H_16_^35^ClN_2_O_2_^+^ requires 339.0900.

### 3.6. Typical Procedure for the Suzuki-Miyaura-Miyaura Cross-Coupling of ***4a**–**h***

#### 3.6.1. 4-(4-Fluorophenyl)-2-phenyl-6-(phenylethynyl)quinazoline (**5a**)

A stirred mixture of **4a** (0.10 g, 0.29 mmol), PdCl_2_(PPh_3_)_2_ (0.009 g, 0.014 mmol), PCy_3_ (0.008 g, 0.029 mmol) and K_2_CO_3_ (0.060 g, 0.43 mmol) in 3:1 dioxane/water (*v*/*v*; 10 mL) was purged with argon gas for 30 min. 4-Fluorophenylboronic acid (0.048 g, 0.35 mmol) was added to the mixture using a syringe. The reaction mixture was heated at 100 °C for 2 h and then quenched with an ice-cold water. The product was extracted into chloroform and the combined organic layers were washed with water, dried over Na_2_SO_4_, filtered and evaporated under reduced pressure. The residue was purified by column chromatography on silica gel to afford **5a** as a yellow solid (0.089 g, 76%), R*_f_* (2:1 hexane/toluene) 0.40, mp. 177–180 °C; ν_max_ (ATR) cm^−1^ 520, 570, 595, 685, 708, 752, 846, 1157, 1230, 1400, 1538, 1607, 2204; δ_H_ (500 MHz, DMSO-*d*_6_) 7.33 (t, *J* = 8.5 Hz, 2H), 7.38–7.39 (m, 3H), 7.52–7.57 (m, 5H), 7.93 (dd, *J* = 6.5 and 8.5 Hz, 2H), 7.98 (d, *J* = 8.5 Hz, 1H), 8.12 (d, *J* = 8.5 Hz, 1H), 8.24 (d, *J* = 1.5 Hz, 1H), 8.68 (d, *J* = 6.5 Hz, 2H); δ_C_ (125 MHz, DMSO-*d*_6_) 88.7, 91.4, 115.8 (d, ^2^*J*_CF_= 21.7 Hz), 121.4, 122.2, 122.5, 128.4, 128.6, 128.7, 128.8, 129.4, 129.7, 130.8, 131.7, 132.2 (d, ^3^*J*_CF_= 8.6 Hz), 133.3 (d, ^4^*J*_CF_= 2.7 Hz), 136.3, 137.8, 151.5, 160.5, 164.0 (d, ^1^*J*_CF_= 249.3 Hz), 166.8; *m*/*z* 401 (100, MH^+^); HRMS (ES): MH^+^, found 401.1448. C_28_H_18_FN_2_^+^ requires 401.1454.

#### 3.6.2. 2,4-Bis(4-fluorophenyl)-6-(phenylethynyl)quinazoline (**5b**)

A mixture of **4b** (0.10 g, 0.28 mmol), PdCl_2_(PPh_3_)_2_ (0.009 g, 0.014 mmol), PCy_3_ (0.008 g, 0.028 mmol), 4-fluorophenylboronic acid (0.046 g, 0.33 mmol) and K_2_CO_3_ (0.060 g, 0.43 mmol) in aqueous dioxane (10 mL) afforded **5b** as a yellow solid (0.10 g, 85%), R*_f_* (2:1 hexane/toluene) 0.50, mp. 220–222 °C; ν_max_ (ATR) 523, 560, 588, 685, 750, 802, 840, 1027, 1156, 1220, 1244, 1300, 1400, 1509, 1536, 1600, 2211 cm^−1^; δ_H_ (500 MHz, CDCl_3_) 7.21 (t, *J* = 8.5 Hz, 2H), 7.34 (t, *J* = 8.5 Hz, 2H), 7.37–7.39 (m, 3H), 7.56–7.58 (m, 2H), 7.92 (dd, *J* = 5.5 and 8.5 Hz, 2H), 7.99 (dd, *J* = 2.0 and 8.5 Hz, 1H), 8.10 (d, *J* = 8.5 Hz, 1H), 8.24 (d, *J* = 2.0 Hz, 1H), 8.71 (t, *J* = 8.5 Hz, 2H); δ_C_ (125 MHz, CDCl_3_) 88.6, 91.5, 115.5 (d, ^2^*J*_CF_ = 21.8 Hz), 115.9 (d, ^2^*J*_CF_ = 21.8 Hz), 121.3, 122.3, 122.5, 128.4, 128.8, 129.3, 129.7, 130.8 (d, ^3^*J*_CF_ = 8.6 Hz), 131.7, 132.2 (d, ^3^*J*_CF_ = 8.6 Hz), 133.3 (d, ^4^*J*_CF_ = 3.7 Hz), 134.0 (d, ^4^*J*_CF_ = 3.1 Hz), 136.4, 151.4, 159.6, 164.1 (d, ^1^*J*_CF_ = 249.3 Hz), 164.8 (d, ^1^*J*_CF_ = 249.3 Hz), 166.8; *m*/*z* 419 (100, MH^+^); HRMS (ES): MH^+^, found 419.1362. C_28_H_17_F_2_N_2_^+^ requires 419.1360.

#### 3.6.3. 4-(4-Fluorophenyl)-2-(4-chlorophenyl)-6-(phenylethynyl)quinazoline (**5c**)

A mixture of **4c** (0.10 g, 0.26 mmol), PdCl_2_(PPh_3_)_2_ (0.009 g, 0.013 mmol), PCy_3_ (0.007 g, 0.026 mmol), 4-fluorophenylboronic acid (0.044 g, 0.32 mmol) and K_2_CO_3_ (0.053 g, 0.39 mmol) in aqueous dioxane (10 mL) afforded **5c** as a yellow solid (0.094 g, 83%), R*_f_* (2:1 hexane/toluene) 0.50, mp. 206–207 °C; ν_max_ (ATR) 499, 525, 571, 588, 689, 750, 802, 836, 1013, 1089, 1160, 1228, 1314, 1416, 1509, 1536, 1602, 2207 cm^−1^; δ_H_ (500 MHz, CDCl_3_) 7.34 (t, *J* = 9.0 Hz, 2H), 7.38–7.39 (m, 3H), 7.50 (d, *J* = 8.5 Hz, 2H), 7.56–7.57 (m, 2H), 7.91 (t, *J* = 8.5 Hz, 2H), 7.98 (dd, *J* = 1.5 and 8.5 Hz, 1H), 8.09 (d, *J* = 8.5 Hz, 1H), 8.23 (d, *J* = 1.5 Hz, 1H), 8.63 (d, *J* = 8.5 Hz, 2H); δ_C_ (125 MHz, CDCl_3_) 88.7, 91.7, 115.9 (d, ^2^*J*_CF_ = 21.7 Hz), 121.4, 122.5, 122.6, 128.5, 128.7, 128.8, 129.4, 129.7, 130.0, 131.7, 132.2 (d, ^3^*J*_CF_ = 8.5 Hz), 133.3 (d, ^4^*J*_CF_ = 3.7 Hz), 136.3, 136.4, 137.1, 151.4, 159.5, 164.1 (d, ^1^*J*_CF_ = 249.4 Hz), 166.8; *m*/*z* 435 (100, MH^+^); HRMS (ES): MH^+^, found 435.1057. C_28_H_17_N_2_F^35^Cl^+^ requires 435.1064.

#### 3.6.4. 4-(4-Fluorophenyl)-2-(4-methoxyphenyl)-6-(phenylethynyl)quinazoline (**5d**)

A mixture of **4d** (0.10 g, 0.27 mmol), PdCl_2_(PPh_3_)_2_ (0.009 g, 0.013 mmol), PCy_3_ (0.007 g, 0.027 mmol), 4-fluorophenylboronic acid (0.044 g, 0.32 mmol) and K_2_CO_3_ (0.056 g, 0.40 mmol) in aqueous dioxane (10 mL) afforded **5d** as a yellow solid (0.091 g, 78%), R*_f_* (2:1 hexane/toluene) 0.25, mp. 198–200 °C; ν_max_ (ATR) 525, 571, 588, 685, 750, 802, 842, 1027, 1161, 1245, 1300, 1401, 1510, 1536, 1601, 2211 cm^−1^; δ_H_ (500 MHz, CDCl_3_) 3.91 (s, 3H), 7.05 (d, *J* = 8.5 Hz, 2H), 7.33 (t, *J* = 9.0 Hz, 2H), 7.37–7.39 (m, 3H), 7.56 (dd, *J* = 2.5 and 6.0 Hz, 2H), 7.92 (dd, *J* = 5.5 and 8.5 Hz, 2H), 7.96 (dd, *J* = 1.5 and 9.0 Hz, 1H), 8.08 (d, *J* = 8.5 Hz, 1H), 8.22 (d, *J* = 1.5 Hz, 1H), 8.65 (d, *J* = 9.0 Hz, 2H); δ_C_ (125 MHz, CDCl_3_) 55.4, 88.8, 91.2, 113.9, 115.8 (d, ^2^*J*_CF_ = 21.8 Hz), 121.1, 121.7, 122.6, 128.4, 128.6, 128.7, 129.1, 129.8, 130.3, 131.7, 132.1 (d, ^3^*J*_CF_ = 8.5 Hz), 133.5 (d, ^4^*J*_CF_ = 3.7 Hz), 136.2, 151.6, 160.3, 162.0, 164.0 (d, ^1^*J*_CF_ = 249.4 Hz), 166.6; *m*/*z* 431 (100, MH^+^); HRMS (ES): MH^+^, found 431.1556. C_29_H_20_FN_2_O^+^ requires 431.1560.

#### 3.6.5. 2-(4-Fluorophenyl)-4-(4-methoxyphenyl)-6-(phenylethynyl)quinazoline (**5e**)

A mixture of **4b** (0.10 g, 0.28 mmol), PdCl_2_(PPh_3_)_2_ (0.009 g, 0.014 mmol), PCy_3_ (0.008 g, 0.028 mmol), 4-methoxyphenylboronic acid (0.050 g, 0.33 mmol) and K_2_CO_3_ (0.058 g, 0.42 mmol) in aqueous dioxane (10 mL) afforded **5e** as a yellow solid (0.096 g, 79%), R*_f_* (2:1 hexane/toluene) 0.30, mp. 197–200 °C; ν_max_ (ATR) 591, 687, 754, 841, 1145, 1174, 1223, 1255, 1397, 1508, 1534, 1610, 2196 cm^−1^; δ_H_ (500 MHz, CDCl_3_) 3.96 (s, 3H), 7.16 (d, *J* = 9.0 Hz, 2H), 7.21 (t, *J* = 8.5 Hz, 2H), 7.37–7.39 (m, 3H), 7.56–7.58 (m, 2H), 7.91 (d, *J* = 9.0 Hz, 2H), 7.98 (dd, *J* = 2.0 and 9.0 Hz, 1H), 8.08 (d, *J* = 8.5 Hz, 1H), 8.33 (d, *J* = 2.0 Hz, 1H), 8.71 (dt, *J* = 3.5 and 8.5 Hz, 2H); δ_C_ (125 MHz, CDCl_3_) 55.5, 88.9, 91.2, 114.2, 115.5 (d, ^2^*J*_CF_ = 21.8 Hz), 121.4, 121.9, 122.7, 128.5, 128.7, 129.2, 129.8, 130.3, 130.8 (d, ^3^*J*_CF_ = 8.6 Hz), 131.7, 131.9, 134.2 (d, ^4^*J*_CF_ = 2.8 Hz), 136.1, 151.5, 159.6, 161.5, 164. (d, ^1^*J*_CF_ = 248.3 Hz), 167.5; *m*/*z* 431 (100, MH^+^); HRMS (ES): MH^+^, found 431.1554. C_29_H_20_FN_2_O^+^ requires 431.1560.

#### 3.6.6. 2-(4-Chlorophenyl)-4-(4-methoxyphenyl)-6-(2-phenylethynyl)quinazoline (**5f**)

A mixture of **4c** (0.10 g, 0.26 mmol), PdCl_2_(PPh_3_)_2_ (0.009 g, 0.013 mmol), PCy_3_ (0.007 g, 0.026 mmol), 4-methoxyphenylboronic acid (0.048 g, 0.32 mmol) and K_2_CO_3_ (0.053 g, 0.39 mmol) in aqueous dioxane (10 mL) afforded **5f** as a yellow solid (0.082 g, 70%), R*_f_* (2:1 hexane/toluene) 0.37, mp. 186–187 °C; ν_max_ (ATR) 524, 539, 581, 685, 751, 803, 841, 1012, 1090, 1256, 1395, 1531, 1609, 2204 cm^−1^; δ_H_ (500 MHz, CDCl_3_) 3.96 (s, 3H), 7.17 (d, *J* = 9.0 Hz, 2H), 7.38–7.39 (m, 3H), 7.50 (d, *J* = 8.5 Hz, 2H), 7.56–7.58 (m, 2H), 7.91 (d, *J* = 8.5 Hz, 2H), 7.97 (d, *J* = 8.5 Hz, 1H), 8.08 (d, *J* = 7.0 Hz, 1H), 8.33 (s, 1H), 8.65 (d, *J* = 8.0 Hz, 2H); δ_C_ (125 MHz, CDCl_3_) 55.5, 88.8, 91.3, 114.2, 121.5, 122.1, 122.6, 128.6, 128.4, 128.7, 129.0, 129.2, 130.0, 130.2, 131.7, 131.8, 136.2, 136.5, 136.8, 151.4, 159.5, 161.4, 167.4; *m*/*z* 447 (100, MH^+^); HRMS (ES): MH^+^, found 447.1249. C_29_H_20_^35^ClN_2_O^+^ requires 447.1264. 

#### 3.6.7. 2,4-Bis(4-methoxyphenyl)-6-(phenylethynyl)quinazoline (**5g**)

A mixture of **4d** (0.10 g, 0.27 mmol), PdCl_2_(PPh_3_)_2_ (0.009 g, 0.013 mmol), PCy_3_ (0.007 g, 0.027 mmol), 4-methoxyphenylboronic acid (0.049 g, 0.32 mmol) and K_2_CO_3_ (0.056 g, 0.40 mmol) in aqueous dioxane (10 mL) afforded **5g** as a yellow solid (0.095 g, 79%), R*_f_* (2:1 hexane/toluene) 0.14, mp. 170–172 °C; ν_max_ (ATR) 525, 574, 685, 749, 801, 840, 1024, 1159, 1246, 1299, 1399, 1511, 1532, 1604, 2202 cm^−1^; δ_H_ (500 MHz, CDCl_3_) 3.91 (d, *J* = 2.0 Hz, 3H), 3.95 (d, *J* = 2.5 Hz, 3H), 7.05 (dd, *J* = 2.5 and 8.5 Hz, 2H), 7.16 (dd, *J* = 2.5 and 9.0 Hz, 2H), 7.37–7.38 (m, 3H), 7.55–7.57 (m, 2H), 7.91 (dd, *J* = 2.5 and 8.5 Hz, 2H), 7.94 (dd, *J* = 2.0 and 8.5 Hz, 1H), 8.05 (dd, *J* = 2.5 and 9.0 Hz, 1H), 8.31 (s, 1H), 8.66 (dd, *J* = 2.5 and 8.5 Hz, 2H); δ_C_ (125 MHz, CDCl_3_) 55.4, 55.5, 89.0, 90.9, 13.9, 114.2, 121.2, 121.3, 122.8, 128.4, 128.6, 129.0, 129.9, 130.3, 130.4, 130.7, 131.7, 131.8, 136.0, 151.7, 160.4, 161.4, 161.9, 167.3; *m*/*z* 443 (100, MH^+^); HRMS (ES): MH^+^, found 443.1750. C_30_H_23_N_2_O_2_^+^ requires 443.1760.

#### 3.6.8. 4-(4-(4-Methoxyphenyl)-2-phenylquinazolin-6-yl)but-3-yn-1-ol (**5h**)

A mixture of **4e** (0.10 g, 0.32 mmol), PdCl_2_(PPh_3_)_2_ (0.011 g, 0.016 mmol), PCy_3_ (0.009 g, 0.032 mmol), 4-fluorophenylboronic acid (0.058 g, 0.38 mmol) and K_2_CO_3_ (0.066 g, 0.48 mmol) in aqueous dioxane (10 mL) afforded **5h** as a yellow solid (0.110 g, 90%), R*_f_* (2:1 hexane/ethyl acetate) 0.26, mp. 181–183 °C; ν_max_ (ATR) 528, 568, 687, 751, 800, 848, 1026, 1167, 1242, 1408, 1511, 1532, 2210, 3032, 3060 cm^−1^; δ_H_ (500 MHz, CDCl_3_) 1.81 (s, 1H), 2.75 (t, *J* = 6.5 Hz, 2H), 3.86 (t, *J* 5.5 Hz, 2H), 3.95 (s, 3H), 7.15 (d, *J* = 8.0 Hz, 2H), 7.51–7.55 (m, 3H), 7.85 (dd, *J* = 1.5 and 8.5 Hz, 1H), 7.89 (d, *J* = 9.0 Hz, 2H), 8.06 (d, *J* = 9.0 Hz, 1H), 8.22 (s, 1H), 8.69 (dd, *J* = 1.5 and 8.5 Hz, 2H); δ_C_ (125 MHz, CDCl_3_) 23.9, 55.4, 61.0, 81.9, 88.5, 114.1, 121.3, 121.9, 128.5, 128.6, 129.1, 129.8, 130.2, 130.6, 131.8, 136.2, 138.0, 151.4, 160.4, 161.3, 167.3; *m*/*z* 381 (100, MH^+^); HRMS (ES): MH^+^, found 381.1597. C_25_H_21_N_2_O_2_^+^ requires 381.1603.

### 3.7. Typical Procedure for the One-Pot Sequential Sonogashira cross-Coupling of ***2a**–**d*** with Terminal Alkynes 

#### 3.7.1. 4-(2-Phenyl-6-(phenylethynyl)quinazolin-4-yl)but-3-yn-1-ol (**6a**)

A stirred mixture of **3a** (0.30 g, 0.82 mmol), PdCl_2_(PPh_3_)_2_ (0.057 g, 0.082 mmol), CuI (0.008 g; 0.041 mmol) and Cs_2_CO_3_ (0.40 g, 1.23 mmol) in THF (20 mL) was purged with argon gas for 30 min. Phenylacetylene (0.084 g, 0.82 mmol) was added to the mixture using a syringe. The reaction mixture was stirred at room temperature for 18 h until the starting material was consumed (tlc monitoring). A solution of 3-butyn-1-ol (0.069 g, 0.98 mmol) in THF (5 mL) was added by means of a syringe and the mixture was heated at 80 °C for 2 h and then quenched with an ice-cold water. The product was extracted into chloroform and the combined organic layers were washed with water, dried over Na_2_SO_4_, filtered and evaporated under reduced pressure. The residue was purified by column chromatography on silica gel to afford **6a** as a yellow solid (0.24 g, 78%), R*_f_* (1:1 hexane/ethyl acetate) 0.65, mp. 163–164 °C; ν_max_ (ATR) 522, 527, 690, 709, 748, 837, 1059, 1402, 1531, 1737, 2229, 3059, 3256 cm^−1^; δ_H_ (500 MHz, CDCl_3_) 2.74 (br s, 1H), 2.95 (t, *J* = 6.5 Hz, 2H), 4.02 (t, *J* = 6.5 Hz, 2H), 7.39–7.42 (m, 3H), 7.51–7.55 (m, 3H), 7.61–7.63 (m, 2H), 7.95 (dd, *J* = 2.0 and 8.5 Hz, 1H), 8.00 (d, *J* = 9.0 Hz, 1H), 8.35 (d, *J* = 2.0 Hz, 1H), 8.56–8.58 (m, 2H); δ_C_ (125 MHz, CDCl_3_) 24.3, 60.6, 78.8, 88.7, 91.9, 97.8, 122.6, 122.7, 123.7, 128.5, 128.6, 128.7, 128.8, 129.0, 129.5, 130.7, 131.8, 136.9, 137.4, 150.3, 152.3, 161.1; *m*/*z* 375 (100, MH^+^); HRMS (ES): MH^+^, found 375.1487. C_26_H_19_N_2_O^+^ requires 375.1497.

#### 3.7.2. 4-(2-(4-Fluorophenyl)-6-(phenylethynyl)quinazolin-4-yl)but-3-yn-1-ol (**6b**)

A mixture of **3b** (0.30 g, 0.78 mmol), PdCl_2_(PPh_3_)_2_ (0.054 g, 0.078 mmol), CuI (0.007 g, 0.039 mmol), Cs_2_CO_3_ (0.38 g, 1.17 mmol) and phenylacetylene (0.079 g, 0.78 mmol) in THF (20 mL), followed by the addition of 3-butyn-1-ol (0.065 g, 0.93 mmol) in THF (5 mL) was treated as described for **6a** to afford **6b** as a yellow solid (0.17 g, 55%), R*_f_* (1:1 hexane/ethyl acetate) 0.60, mp. 174–175 °C; ν_max_ (ATR) 528, 570, 691, 744, 758, 839, 1060, 1154, 1224, 1295, 1404, 1532, 2230, 3050, 3271 cm^−1^; δ_H_ (500 MHz, CDCl_3_) 2.25 (br s, 1H), 2.98 (t, *J* = 6.0 Hz, 2H), 4.04 (d, *J* = 6.0 Hz, 2H), 7.20 (t, *J* = 8.5 Hz, 2H), 7.40–7.41 (m, 3H), 7.61–7.63 (m, 2H), 7.96 (dd, *J* = 2.0 and 8.5 Hz, 1H), 8.00 (d, *J* = 8.5 Hz, 1H), 8.39 (d, *J* = 2.0 Hz, 1H), 8.61 (t, *J* = 8.5 Hz, 2H); δ_C_ (125 MHz, CDCl_3_) 24.3, 60.6, 78.6, 88.6, 91.9, 98.1, 115.6 (d, ^2^*J*_CF_ = 21.8 Hz), 122.6, 122.7, 123.5, 128.5, 128.9, 129.4, 130.8 (d, ^3^*J*_CF_ = 8.5 Hz), 131.8, 133.7 (d, ^4^*J*_CF_ = 3.0 Hz), 137.0, 146.1, 150.2, 152.3, 160.1, 164.8 (d, ^1^*J*_CF_ = 248.3 Hz); *m*/*z* 393 (100, MH^+^); HRMS (ES): MH^+^, found 393.1412. C_26_H_18_FN_2_O^+^ requires 393.1403.

#### 3.7.3. 4-(2-(4-Chlorophenyl)-6-(phenylethynyl)quinazolin-4-yl)but-3-yn-1-ol (**6c**)

A mixture of **3c** (0.30 g, 0.75 mmol), PdCl_2_(PPh_3_)_2_ (0.052 g, 0.074 mmol), CuI (0.007 g, 0.037 mmol), Cs_2_CO_3_ (0.36 g, 1.12 mmol) and phenylacetylene (0.076 g, 0.75 mmol) in THF (20 mL), followed by the addition of 3-butyn-1-ol (0.062 g, 0.89 mmol) in THF (5 mL) was treated as described for **6a** to afford **6c** as a yellow solid (0.20 g, 65%), R*_f_* (1:1 hexane/ethyl acetate) 0.70, mp. 202–204 °C; ν_max_ (ATR) 525, 567, 684, 737, 749, 837, 1039, 1087, 1296, 1402, 1532, 2233, 3048, 3252 cm^−1^; δ_H_ (500 MHz, CDCl_3_) 2.40 (br s, 1H), 2.97 (t, *J* = 6.5 Hz, 2H), 4.03 (t, *J* = 6.5 Hz, 2H), 7.40–7.41 (m, 3H), 7.48 (d, *J* = 8.5 Hz, 2H) 7.60–7.62 (m, 2H), 7.94 (d, *J* = 8.5 Hz, 2H), 8.31 (s, 1H), 8.50 (d, *J* = 8.5 Hz, 2H); δ_C_ (125 MHz, CDCl_3_) 24.3, 60.6, 78.6, 88.6, 92.1, 98.1 122.6, 122.9, 123.6, 128.5, 128.8, 128.9, 129.0, 129.4, 130.0, 131.8, 135.9, 137.0, 137.1, 150.3, 152.3, 160.0; *m*/*z* 409 (100, MH^+^); HRMS (ES): MH^+^, found 409.1104. C_26_H_18_N_2_O^35^Cl^+^ requires 409.1108.

#### 3.7.4. 4-(2-(4-Methoxyphenyl)-6-(phenylethynyl)quinazolin-4-yl)but-3-yn-1-ol (**6d**)

A mixture of **3d** (0.30 g, 0.75 mmol), PdCl_2_(PPh_3_)_2_ (0.053 g, 0.075 mmol), CuI (0.007 g, 0.037 mmol), Cs_2_CO_3_ (0.36 g, 1.12 mmol) and phenylacetylene (0.076 g, 0.75 mmol) in THF (20 mL), followed by the addition of 3-butyn-1-ol (0.063 g, 0.90 mmol) in THF (5 mL) was treated as described for **6a** to afford **6d** as a yellow solid (0.20 g, 66%), R*_f_* (1:1 hexane/ethyl acetate) 0.50, mp. 141–142 °C; ν_max_ (ATR) 527, 569, 690, 748, 837, 1027, 1059, 1217, 1402, 1531, 1737, 2229, 3040, 3256 cm^−1^; δ_H_ (500 MHz, CDCl_3_) 2.74 (br s, 1H), 2.95 (t, *J* = 6.5 Hz, 2H), 3.89 (s, 3H), 4.02 (t, *J* = 6.5 Hz, 2H), 7.02 (d, *J* = 9.0 Hz, 2H), 7.39–7.41 (m, 3H), 7.60–7.62 (m, 2H), 7.76 (dd, *J* = 2.0 and 8.5 Hz, 1H), 7.94 (d, *J* = 8.5 Hz, 1H), 8.31 (d, *J* = 2.0 Hz, 1H), 8.52 (d, *J* = 8.5 Hz, 2H); δ_C_ (125 MHz, CDCl_3_) 24.4, 55.4, 60.6, 78.7, 88.8, 91.6, 97.7, 113.9, 122.1, 122.7, 123.3, 128.5, 128.7, 128.8, 129.5, 130.0, 130.4, 131.8, 136.8, 150.3, 152.2, 160.8, 162.1; *m*/*z* 405 (100, MH^+^); HRMS (ES): MH^+^, found 405.1600. C_27_H_21_N_2_O_2_^+^ requires 405.1603.

#### 3.7.5. 4-(2-Phenyl-4-(phenylethynyl)quinazolin-6-yl)but-3-yn-1-ol (**6e**)

A mixture of **3a** (0.30 g, 0.82 mmol), PdCl_2_(PPh_3_)_2_ (0.057 g, 0.082 mmol), CuI (0.008 g, 0.041 mmol), Cs_2_CO_3_ (0.40 g, 1.23 mmol) and 3-butyn-1-ol (0.057 g, 0.82 mmol) in THF (20 mL), followed by the addition of phenylacetylene (0.10 g, 0.98 mmol) in THF (5 mL) was treated as described for **6a** to afford **6e** as a yellow solid (0.19 g, 61%), R*_f_* (1:1 hexane/ethyl acetate) 0.67, mp. 126–127 °C; ν_max_ (ATR) 546, 687, 708, 755, 838, 1037, 1309, 1409, 1527, 2208, 2922, 3058, 3358 cm^−1^; δ_H_ (500 MHz, CDCl_3_) 1.89 (s, 1H), 2.80 (t, *J* = 5.5 Hz, 2H), 3.90 (t, *J* = 5.5 Hz, 2H), 7.48–7.55 (m, 6H), 7.81 (d, *J* = 6.5 Hz, 2H), 7.88 (d, *J* = 8.5 Hz, 1H), 8.02 (d, *J* = 8.5 Hz, 1H), 8.41 (s, 1H), 8.64 (d, *J* = 6.5 Hz, 2H); δ_C_ (125 MHz, CDCl_3_) 23.9, 61.0, 81.8, 85.4, 89.4, 982, 121.2, 122.8, 123.6, 128.6, 128.7, 128.8, 129.0, 129.4, 130.2, 130.8, 132.6, 137.1, 137.5, 150.3, 152.3 161.2; *m*/*z* 375 (100, MH^+^); HRMS (ES): MH^+^, found 375.1495. C_26_H_19_N_2_O^+^ requires 375.1497.

#### 3.7.6. 4-(2-(4-Fluorophenyl)-4-(phenylethynyl)quinazolin-6-yl)but-3-yn-1-ol (**6f**)

A mixture of **3b** (0.30 g, 0.78 mmol), PdCl_2_(PPh_3_)_2_ (0.054 g, 0.078 mmol), CuI (0.007 g, 0.039 mmol), Cs_2_CO_3_ (0.38 g, 1.17 mmol) and 3-butyn-1-ol (0.054 g, 0.78 mmol) in THF (20 mL), followed by the addition of phenylacetylene (0.095 g, 0.93 mmol) in THF (5 mL) was treated as described for **6a** to afford **6f** as a yellow solid (0.16 g, 52%), R*_f_* (1:1 hexane/ethyl acetate) 0.50, mp. 150–152 °C; ν_max_ (ATR) 566, 690, 742, 760, 835, 1037, 1148, 1221, 1308, 1404, 1509, 1527, 2209, 2922, 3316 cm^−1^; δ_H_ (500 MHz, CDCl_3_) 2.02 (br s, 1H), 2.79 (t, *J* = 6.0 Hz, 2H), 3.90 (t, *J* = 6.0 Hz, 2H), 7.20 (t, *J* = 9.0 Hz, 2H), 7.46–7.51 (m, 3H), 7.79 (d, *J* = 7.5 Hz, 2H), 7.85 (dd, *J* = 2.0 and 8.5 Hz, 1H), 7.97 (d, *J* = 9.0 Hz, 1H), 8.36 (d, *J* = 2.0 Hz, 1H), 8.64 (t, *J* = 7.5 Hz, 2H); δ_C_ (125 MHz, CDCl_3_) 25.0, 61.1, 81.7, 85.3, 89.5, 98.4, 115.6 (d, ^2^*J*_CF_ = 21.8 Hz), 121.2, 122.9, 123.5, 128.7, 128.9, 129.4, 130.3, 130.9 (d, ^3^*J*_CF_ = 8.6 Hz), 131.7, 133.5 (d, ^4^*J*_CF_ = 3.5 Hz), 136.9, 137.2, 150.2, 152.3, 160.2, 164.8 (d, ^1^*J*_CF_ = 249.3 Hz); *m*/*z* 393 (100, MH^+^); HRMS (ES): MH^+^, found 393.1396. C_26_H_18_FN_2_O^+^ requires 393.1403.

#### 3.7.7. 4-(2-(4-Chlorophenyl)-4-(phenylethynyl)quinazolin-6-yl)but-3-yn-1-ol (**6g**)

A mixture of **3c** (0.30 g, 0.75 mmol), PdCl_2_(PPh_3_)_2_ (0.052 g, 0.074 mmol), CuI (0.007 g, 0.037 mmol), Cs_2_CO_3_ (0.36 g, 1.12 mmol) and 3-butyn-1-ol (0.052 g, 0.75 mmol) in THF (20 mL), followed by the addition of phenylacetylene (0.091 g, 0.89 mmol) in THF (5 mL) was treated as described for **6a** to afford **6g** as a yellow solid (0.23 g, 75%), R*_f_* (1:1 hexane/ethyl acetate) 0.40, mp. 181–182 °C; ν_max_ (ATR) 569, 683, 736, 748, 838, 1012, 1089, 1167, 1308, 1401, 1530, 2213, 2921, 3256 cm^−1^; δ_H_ (500 MHz, CDCl_3_) 1.86 (br s, 1H), 2.80 (t, *J* = 6.0 Hz, 2H), 3.91 (t, *J* = 6.5 Hz, 2H), 7.47–7.51 (m, 5H), 7.80 (d, *J* = 8.5 Hz, 2H), 7.88 (dd, *J* = 2.0 and 8.5 Hz, 1H), 8.00 (d, *J* = 8.5 Hz, 1H), 8.40 (d, *J* = 2.0 Hz, 1H), 8.60 (d, *J* = 8.5 Hz, 2H); δ_C_ (125 MHz, CDCl_3_) 24.0, 61.1, 81.7, 85.3, 89.7, 98.5, 121.2, 123.1, 123.6, 128.7, 128.8, 128.9, 129.4, 130.0, 130.3, 132.6, 136.0, 137.1, 137.3, 150.2, 152.3, 160.1; *m*/*z* 409 (100, MH^+^); HRMS (ES): MH^+^, found 409.1101. C_26_H_18_N_2_O^35^Cl^+^ requires 409.1108.

#### 3.7.8. 4-(2-(4-Methoxyphenyl)-4-(phenylethynyl)quinazolin-6-yl)but-3-yn-1-ol (**6h**)

A mixture of **3d** (0.30 g, 0.75 mmol), PdCl_2_(PPh_3_)_2_ (0.053 g, 0.075 mmol), CuI (0.007 g, 0.037 mmol), Cs_2_CO_3_ (0.36 g, 1.12 mmol) and 3-butyn-1-ol (0.052 g, 0.75 mmol) in THF (20 mL), followed by the addition of phenylacetylene (0.091 g, 0.90 mmol) in THF (5 mL) was treated as described for **6a** to afford **6h** as a yellow solid (0.18 g, 59%), R*_f_* (1:1 hexane/ethyl acetate) 0.51, mp. 175–177 °C; ν_max_ (ATR) 560, 687, 746, 756, 836, 1025, 1166, 1246, 1306, 1409, 1514, 1528, 2211, 2926, 3418 cm^−1^; δ_H_ (500 MHz, CDCl_3_) 1.91 (br s, 1H), 2.79 (t, *J* = 6.0 Hz, 2H), 3.90 (t, *J* = 6.0 Hz, 2H), 3.91 (s, 3H), 7.04 (d, *J* = 9.0 Hz, 2H), 7.46–7.50 (m, 3H), 7.80 (dd, *J* = 2.0 and 8.0 Hz, 2H), 7.84 (dd, *J* = 1.5 and 9.0 Hz, 1H), 7.96 (d, *J* = 9.0 Hz, 1H), 8.37 (d, *J* = 1.5 Hz, 1H), 8.60 (d, *J* = 9.0 Hz, 2H); δ_C_ (125 MHz, CDCl_3_) 24.0, 55.4, 61.1, 81.8, 85.5, 89.2, 97.9, 113.9, 121.3, 122.3, 123.3, 128.4, 128.6, 128.8, 129.5, 130.1, 130.2, 130.4, 132.6, 137.1, 150.4, 161.0, 162.1; *m*/*z* 405 (100, MH^+^); HRMS (ES): MH^+^, found 405.1602. C_27_H_21_N_2_O_2_^+^ requires 405.1603.

### 3.8. Typical Procedure for the One-Pot Sequential Sonogashira and Stille Cross-Coupling of ***2a**–**d***

#### 3.8.1. 4-(Furan-2-yl)-2-phenyl-6-(phenylethynyl)quinazoline (**7a**)

A stirred mixture of **3a** (0.10 g, 0.27 mmol), PdCl_2_(PPh_3_)_2_ (0.019 g, 0.027 mmol), CuI (0.002 g, 0.013 mmol) and Cs_2_CO_3_ (0.13 g, 0.40 mmol) in THF (10 mL) was purged with argon gas for 30 min. Phenylacetylene (0.027 g, 0.27 mmol) was added to the mixture using a syringe. The reaction mixture was stirred at room temperature for 18 h until the starting material was consumed (tlc monitoring). A solution of 2-(tributylstannyl)furan (0.11 g, 0.32 mmol) in THF (5 mL) was added by means of a syringe and the mixture was heated at 80 °C for 2 h and then quenched with an ice-cold water. The product was extracted into chloroform and the combined organic layers were washed with water, dried over MgSO_4_, filtered and evaporated under reduced pressure. The residue was purified by column chromatography on silica gel to afford **7a** as a yellow solid (0.056 g, 56%), R*_f_* (2:1 hexane/toluene) 0.25, mp. 184–186 °C; ν_max_ (ATR) 525, 592, 686, 703, 752, 918, 996, 1314, 1474, 1532, 2202 cm^−1^; δ_H_ (500 MHz, CDCl_3_) 6.72 (dd, *J* = 1.5 and 3.0 Hz, 1H), 7.39–7.40 (m, 3H), 7.51–7.56 (m, 3H) , 7.61–7.63 (m, 2H), 7.75 (d, *J* = 3.5 Hz, 1H), 7.86 (s, 1H), 7.95 (dd, *J* = 1.5 and 8.5 Hz, 1H), 8.04 (d, *J* = 8.5 Hz, 1H), 8.66 (d, *J* = 7.0 Hz, 2H), 9.10 (d, *J* = 1.5 Hz, 1H); δ_C_ (125 MHz, CDCl_3_) 89.2, 91.1, 112.4, 116.3, 119.3, 122.2, 122.8, 128.4, 128.5, 128.6, 128.7, 129.3, 130.0, 130.6, 131.7, 136.1, 137.8, 146.1, 152.2, 153.9, 154.7, 160.4; *m*/*z* 373 (100, MH^+^); HRMS (ES): MH^+^, found 373.1332. requires 373.1341.

#### 3.8.2. 2-(4-Fluorophenyl)-4-(furan-2-yl)-6-(phenylethynyl)quinazoline (**7b**)

A mixture of **3b** (0.10 g, 0.26 mmol), PdCl_2_(PPh_3_)_2_ (0.018 g, 0.026 mmol), CuI (0.002 g, 0.013 mmol), Cs_2_CO_3_ (0.13 g, 0.39 mmol) and phenylacetylene (0.026 g, 0.26 mmol) in THF (10 mL), followed by the addition of 2-(tributylstannyl)furan (0.11 g, 0.31 mmol) in THF (5 mL) was treated as for **7a** to afford **7b** as a yellow solid (0.062 g, 62%), R*_f_* (2:1 hexane/toluene) 0.30, mp. 195–196 °C; ν_max_ (ATR) 524, 574, 684, 742, 840, 1015, 1220, 1398, 1511, 1534, 2203 cm^−1^; δ_H_ (500 MHz, CDCl_3_) 6.72 (dd, *J* = 1.5 and 3.5 Hz, 1H), 7.39–7.41 (m, 3H), 7.50 (d, *J* = 8.0 Hz, 2H), 7.61–7.62 (m, 2H), 7.73 (d, *J* = 3.5 Hz, 1H), 7.87 (s, 1H), 7.95 (dd, *J* = 2.0 and 8.5 Hz, 1H), 8.01 (d, *J* = 9.0 Hz, 1H), 8.66 (d, *J* = 8.5 Hz, 2H) 9.09 (d, *J* = 1.5 Hz, 1H); δ_C_ (125 MHz, CDCl_3_) 89.1, 91.2, 112.4, 115.4 (d, ^2^*J*_CF_ = 21.7 Hz), 116.3, 119.2, 122.3, 122.8, 128.4, 128.7, 129.1, 130.0, 130.6 (d, ^3^*J*_CF_ = 8.5 Hz), 131.7, 134.0 (d, ^4^*J*_CF_ = 2.7 Hz), 136.2, 146.2, 152.1, 153.8, 154.7, 159.5, 164.7 (d, ^1^*J*_CF_ = 249.3 Hz); *m*/*z* 391 (100, MH^+^); HRMS (ES): MH^+^, found 391.1238. C_26_H_16_N_2_OF^+^ requires 391.1247.

#### 3.8.3. 2-(4-Chlorophenyl)-4-(furan-2-yl)-6-(phenylethynyl)quinazoline (**7c**)

A mixture of **3c** (0.10 g, 0.25 mmol), PdCl_2_(PPh_3_)_2_ (0.018 g, 0.025 mmol), CuI (0.002 g, 0.012 mmol), Cs_2_CO_3_ (0.12 g, 0.37 mmol) and phenylacetylene (0.026 g, 0.25 mmol) in THF (10 mL), followed by the addition of 2-(tributylstannyl)furan (0.11 g, 0.30 mmol) in THF (5 mL) was treated as for **7a** to afford **7c** as a yellow solid (0.070 g, 69%), R*_f_* (2:1 hexane/toluene) 0.36, mp. 164–166 °C; ν_max_ (ATR) 524, 539, 682, 736, 745, 849, 1013, 1089, 1400, 1532, 1580, 2207 cm^−1^; δ_H_ (500 MHz, CDCl_3_) 6.72 (dd, *J* = 1.5 and 3.5 Hz, 1H), 7.39–7.40 (m, 3H), 7.49 (d, *J* = 8.5 Hz, 2H), 7.61–7.62 (m, 2H), 7.72 (d, *J* = 3.5 Hz, 1H), 7.87 (s, 1H), 7.94 (dd, *J* = 1.5 and 8.5 Hz, 1H), 8.01 (d, *J* = 8.5 Hz, 1H), 8.60 (t, *J* = 9.0 Hz, 2H) 9.09 (d, *J* = 1.5 Hz, 1H); δ_C_ (125 MHz, CDCl_3_) 89.1, 91.3, 112.5, 116.4, 119.3, 122.5, 122.7, 128.4, 128.6, 128.7, 129.2, 129.8, 130.0, 131.7, 132.6, 136.3, 136.8, 146.2, 152.1, 153.8, 154.7, 159.4; *m*/*z* 407 (100, MH^+^); HRMS (ES): MH^+^, found 407.0939. C_26_H_16_N_2_O^35^Cl^+^ requires 407.0951.

#### 3.8.4. 4-(Furan-2-yl)-2-(4-methoxyphenyl)-6-(phenylethynyl)quinazoline (**7d**)

A mixture of **3d** (0.10 g, 0.25 mmol), PdCl_2_(PPh_3_)_2_ (0.018 g, 0.025 mmol), CuI (0.002 g, 0.012 mmol), Cs_2_CO_3_ (0.12 g, 0.37 mmol) and phenylacetylene (0.026 g, 0.25 mmol) in THF (10 mL), followed by the addition of 2-(tributylstannyl)furan (0.11 g, 0.30 mmol) in THF (5 mL) was treated as for **7a** to afford **7d** as a yellow solid (0.059 g, 59%), R*_f_* (toluene) 0.50, mp. 148–150 °C; ν_max_ (ATR) 529, 574, 574, 690, 744, 836, 1010, 1163, 1246, 1407, 1514, 2217 cm^−1^; δ_H_ (500 MHz, CDCl_3_) 3.91 (s, 3H), 6.71 (dd, *J* = 1.5 and 3.5 Hz, 1H), 7.05 (d, *J* = 8.5 Hz, 2H), 7.38–741 (m, 3H), 7.60–7.62 (m, 2H), 7.72 (d, *J* = 3.5 Hz, 1H), 7.86 (s, 1H), 7.93 (dd, *J* = 1.5 and 8.5 Hz, 1H), 7.99 (d, *J* = 8.5 Hz, 1H), 8.62 (d, *J* = 8.5 Hz, 2H) 9.07 (d, *J* = 1.5 Hz, 1H); δ_C_ (125 MHz, CDCl_3_) 55.3, 89.3, 90.9, 112.4, 113.8, 116.1, 119.1, 121.7, 122.9, 128.4, 128.6, 129.0, 130.0, 130.2, 130.5, 131.7, 136.0, 146.0, 152.2, 153.9, 154.6, 160.3, 161.9; *m*/*z* 403 (100, MH^+^); HRMS (ES): MH^+^, found 403.1439. C_27_H_19_N_2_O_2_^+^ requires 403.1447.

### 3.9. Computational Method

Compounds **5a**–**h** were fully optimized in the gas phase using density functional theory (DFT) method. [19] The functional used was CAM-B3LYP [20] and the basis sets for all atoms were 6-31G(d,p). The compounds were also fully optimized using the same functional and basis set in CH_2_Cl_2_ as the solvent based on the Polarizable Continuum Model (PCM) as developed by Tomasi *et al*. [21,22] Frequency computations were performed to identify the nature of the stationary points. All computations were carried out using Gaussian09 [23] running on Gridchem [24,25].

## 4. Conclusions

In summary, we have demonstrated that the 2-aryl-4-chloro-6-iodoquinazolines undergo palladium catalyzed sequential and chemoselective Sonogashira and Suzuki-Miyaura reactions to afford novel unsymmetrical polycarbo-substituted quinazolines with potential photophysical properties. Successful discernment of the relative reactivity of the two C*sp*^2^-halogen bonds (trend: C*sp*^2^-I > C(4)-Cl) of the 2-aryl-4-chloro-6-iodoquinazolines facilitated single-pot bis-Sonogashira and successive Sonogashira/Stille cross-coupling reactions co-catalyzed by PdCl_2_(PPh_3_)_2_ and CuI to afford novel unsymmetrical polycarbo-substituted quinazolines. The one-pot double bifunctionalization approach employed in this investigation is in our opinion cost effective from an economic and environmental point of view because it makes use of a single catalyst mixture and avoids several workup and separation stages involving large quantities of solvents. From a synthesis point of view, it would be interesting to extend this approach towards 4-chloroquinazoline scaffold decorated with two different halogen atoms, e.g., Br and I on the fused benzo ring to establish a general trend in the reactivity of C*sp*^2^-X bond for the polyhalogenated quinazoline series. Preliminary results on the photophysical properties of compounds **5a**–**g**, **6d** and **7d** reveal that the intensity of the absorption and emission bands, Stokes shifts and the fluorescence quantum yields are influenced by the variation of substituents on the *para* position of the 2- and 4-aryl groups. These photophysical property results serve as a pre-requisite to further detailed studies of photophysics and photochemistry as a prelude to compounds with nonlinear optical properties. Likewise, the prevalence of alkynyl and aryl/heteroaryl substituents in biologically relevant polycarbo-substituted quinazolines such as potent epidermal growth factor receptor (EGFR), tyrosine kinase inhibitors [26], and liver X-receptor modulators [27]. Thus polycarbo-substituted quinazolines prepared in this investigation represent suitable candidates for studies of biological activity.

## Supplementary Materials

^1^H-NMR spectra for **5g** in CDCl_3_ and CDCl_3_-TFA mixture (**S1**–**S3)** as well as copies of ^1^H- and ^13^C-NMR spectra of all the new compounds (Figure S4). All the Cartesian coordinates are also listed in the supplementary materials (**S5**). Supplementary materials can be accessed at: http://www.mdpi.com/1420-3049/20/08/14656/s1.

## References

[1] Garcia Y., Schoenebeck F., Legault C.Y., Merlic C.A., Houk K.N. (2009). Theoretical bond dissociation energies of halo-heterocycles: Trends and relationships to regioselectivity in palladium-catalyzed cross-coupling reactions. J. Am. Chem. Soc..

[2] Mangalagiu I., Benneche T., Undheim K. (1996). Trialkalalanes in palladium-catalyzed chemo- and regioselective alkylations. Tetrahedron Lett..

[3] Mangalagiu I., Benneche T., Undheim K. (1996). Ethenylation and alkynylation in palladium-catalyzed carbosubstitution in heteroazines. Acta Chem. Scand..

[4] Charpiot B., Brun J., Donze I., Naef R., Stefani M., Mueller T. (1998). Quinazolines: Combined type 3 and 4 phosphodiesterase inhibitors. Bioorg. Med. Chem. Lett..

[5] Wipf P., George K.M. (2010). Regioselective palladium-catalyzed cross-coupling reactions of 2,4,7-trichloroquinazoline. Synlett.

[6] Sardon T., Cottin T., Xu J., Giannis A., Vernos I. (2009). Development and biological evaluation of a novel aurora a kinase inhibitor. Chem. Biol. Chem..

[7] Mphahlele M.J., Paumo H.K., El-Nahas A.M., El-Hendawy M.M. (2014). Synthesis and photophysical property studies of the 2,6,8-triaryl-4-(phenylethynyl)quinazolines. Molecules.

[8] Achelle S., Rodríguez-López J., Robin-le Guen F. (2014). Synthesis and photophysical studies of a series of quinazoline chromophores. J. Org. Chem..

[9] Liu D., Zhang Z., Zhang H., Wang Y. (2013). A novel approach towards white photoluminescence and electroluminescence by controlled protonation of a blue fluorophore. Chem. Commun..

[10] Legault C.Y., Garcia Y., Merlic C.A., Houk K.N. (2007). Origin of regioselectivity in palladium-catalyzed cross-coupling reactions of polyhalogenated heterocycles. J. Am. Chem. Soc..

[11] Mphahlele M.J., Maluleka M.M. (2014). Advances in metal-catalyzed cross-coupling reactions of halogenated quinazolinones and their quinazoline derivatives. Molecules.

[12] Grushin V.V., Alper H. (1994). Transformation of chloroarenes, catalysed by transition metal complexes. Chem. Rev..

[13] Hassan J., Sévignon M., Gozzi C., Schulz E., Lemaire M. (2002). Aryl-aryl bond formation one century after the discovery of the Ullmann reaction. Chem. Rev..

[14] Kulkarni A.P., Tonzola C.J., Babel A., Jenekhe S.A. (2004). Electron transport materials for organic light-emitting diodes. Chem. Mater..

[15] Aaron J.J., Tine A., Gaye M.D., Parkanyi C., Boniface C., Bieze T.W.N. (1991). Effects of solvent on the electronic absorption and fluorescence spectra of quinazolines, and determination of their ground and excited singlet-state dipole moments. Spectrochim. Acta.

[16] Diaz A.N. (1990). Absorption and emission spectroscopy and photochemistry of 1,10-anthraquinone derivatives. Photochem. Photobiol. A.

[17] Sunahara H., Urano Y., Kojima H., Nagano T. (2007). Design and synthesis of a library of BODIPY-Based environmental polarity sensors utilizing photoinduced electron-transfer-controlled fluorescence on/off switching. J. Am. Chem. Soc..

[18] Rudolph J., Esler W.P., O’Connor S., Coish P.D.G., Wickens P.L., Brands M., Bierer D.E., Bloomquist B.T., Bondar G., Chen L. (2007). Quinazolinone derivatives as orally available Ghrelin Receptor Antagonists for the treatment of diabetes and obesity. J. Med. Chem..

[19] Treutler O., Ahlrichs R. (1995). Efficient molecular numerical integration schemes. J. Chem. Phys..

[20] Yanai T., Tew D., Handy N. (2004). A new hybrid exchange-correlation functional using the Coulomb-attenuating method (CAM-B3LYP). Chem. Phys. Lett..

[21] Tomasi J., Mennucci B., Cammi R. (2005). Quantum mechanical continuum solvation models. Chem. Rev..

[22] Cossi M., Barone V., Cammi R., Tomasi J. (1996). *Ab initio* study of solvated molecules: A new implementation of the polarizable continuum model. Chem. Phys. Lett..

[23] Frisch M.J., Trucks G.W., Schlegel H.B., Scuseria G.E., Robb M.A., Cheeseman J.R., Scalmani G., Barone V., Mennucci B., Petersson G.A. (2010). Gaussian 09.

[24] Shen N., Fan Y., Pamidighantam S. (2014). E-science infrastructures for molecular modeling and parametrization. J. Comput. Sci..

[25] Dooley R., Milfeld K., Guiang C., Pamidighantam S., Allen G. (2006). From proposal to production: Lessons learned developing the computational chemistry grid cyberinfrastructure. J. Grid Comput..

[26] Kitano Y., Suzuki T., Kawahara E., Yamazaki T. (2007). Synthesis and inhibitory activity of 4-alkynyl and 4-alkenylquinazolines: Identification of new scaffolds for potent EGFR tyrosine kinase inhibitors. Bioorg. Med. Chem. Lett..

[27] Bernotas R.C., Ullrich J.W., Travins J.M., Wrobel J.E., Unwalla R.J. (2009). Preparation of quinazoline compounds as modulators of Liver X receptors (LXRs).

