# Supplementary material for: Synthesis and Photophysical Properties of Polycarbo-Substituted Quinazolines Derived from the 2-Aryl-4-chloro-6-iodoquinazolines
Source: Molecules. 2015 Aug 13;20(8):14656–83. doi: 10.3390/molecules200814656 (PMC6332403; doi:10.3390/molecules200814656)
Supplement: Supplementary file 1 [file molecules-20-14656-s001.pdf]

Supporting Information

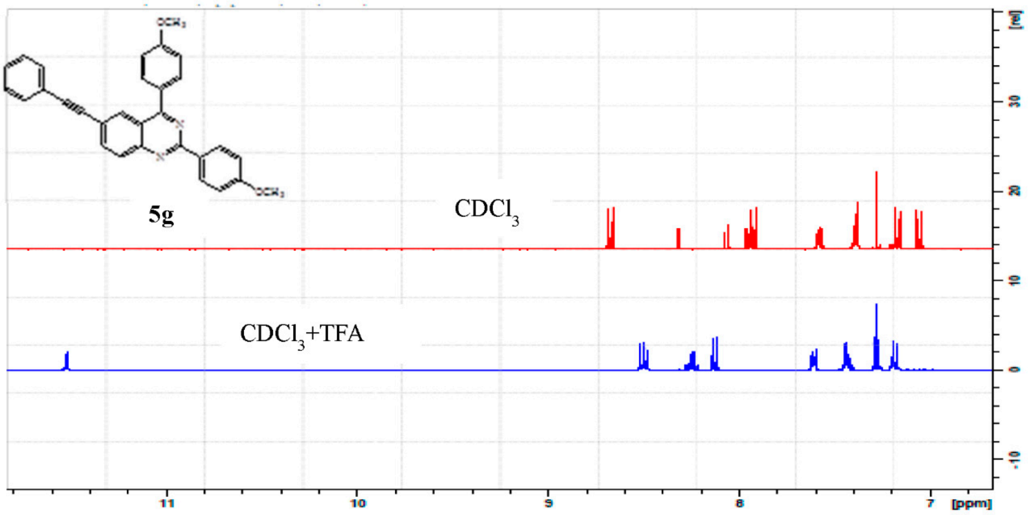

Figure S1. Portion of ^{1}H-NMR spectra of **5g** in $CDCl_3$ (**red**) and in $CDCl_3$+TFA (**blue**).

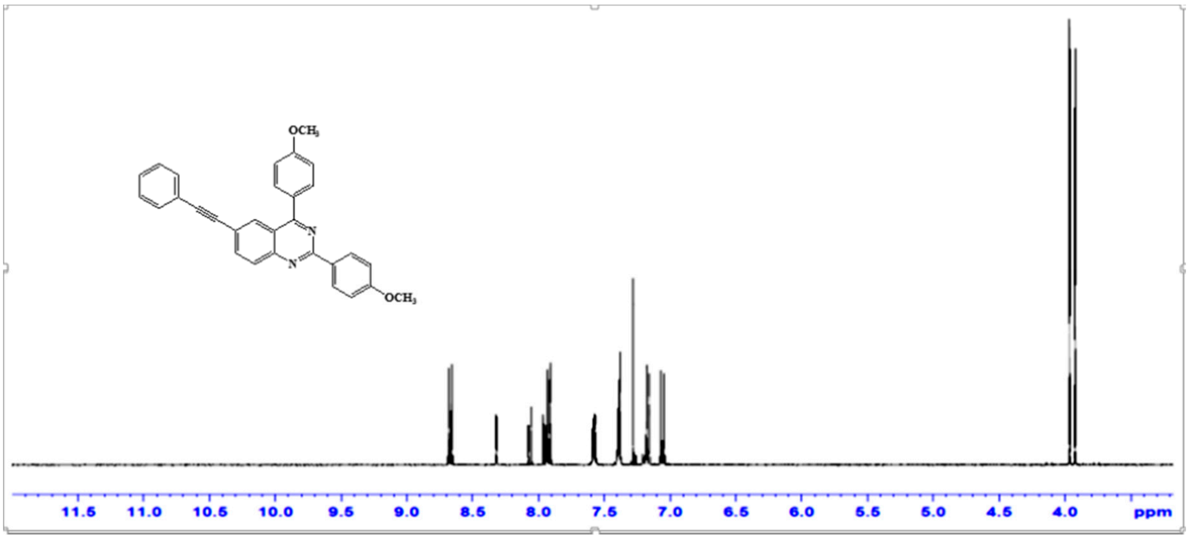

Figure S2. ^{1}H-NMR spectrum of **5g** in $CDCl_3$.

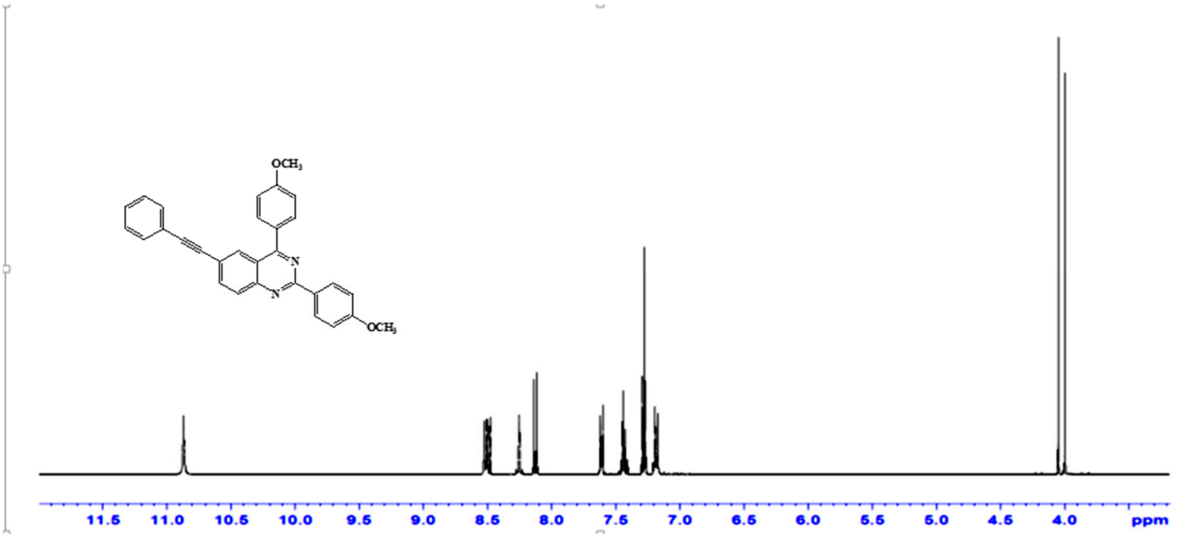

Figure S3. ^{1}H-NMR spectrum of **5g** in $CDCl_3$-TFA mixture.

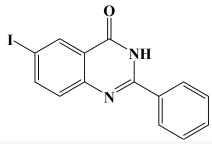

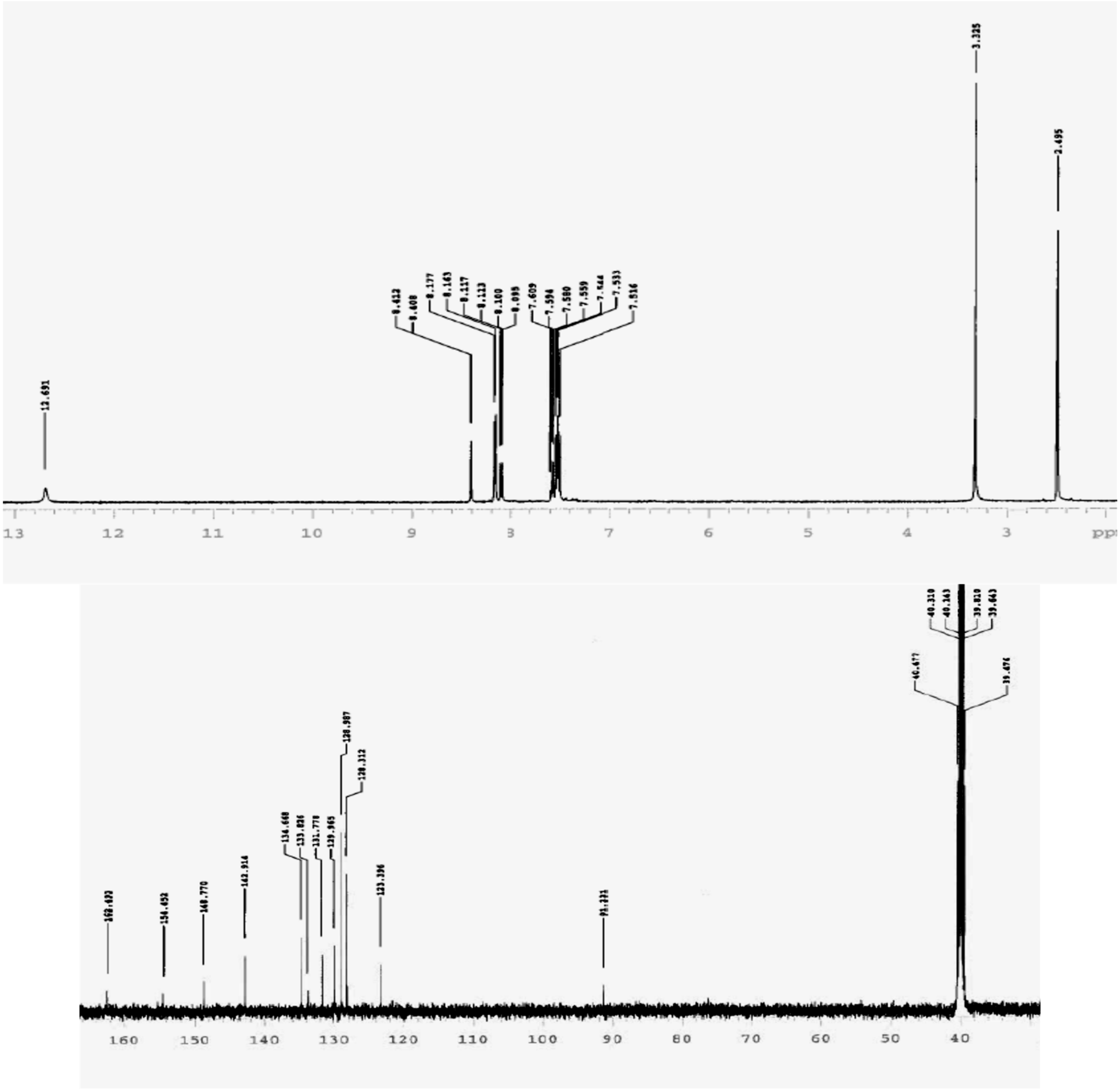

^{1}H-NMR and ^{13}C-NMR Spectra of **2a** (DMSO-d_6).

Figure S4. *Cont.*

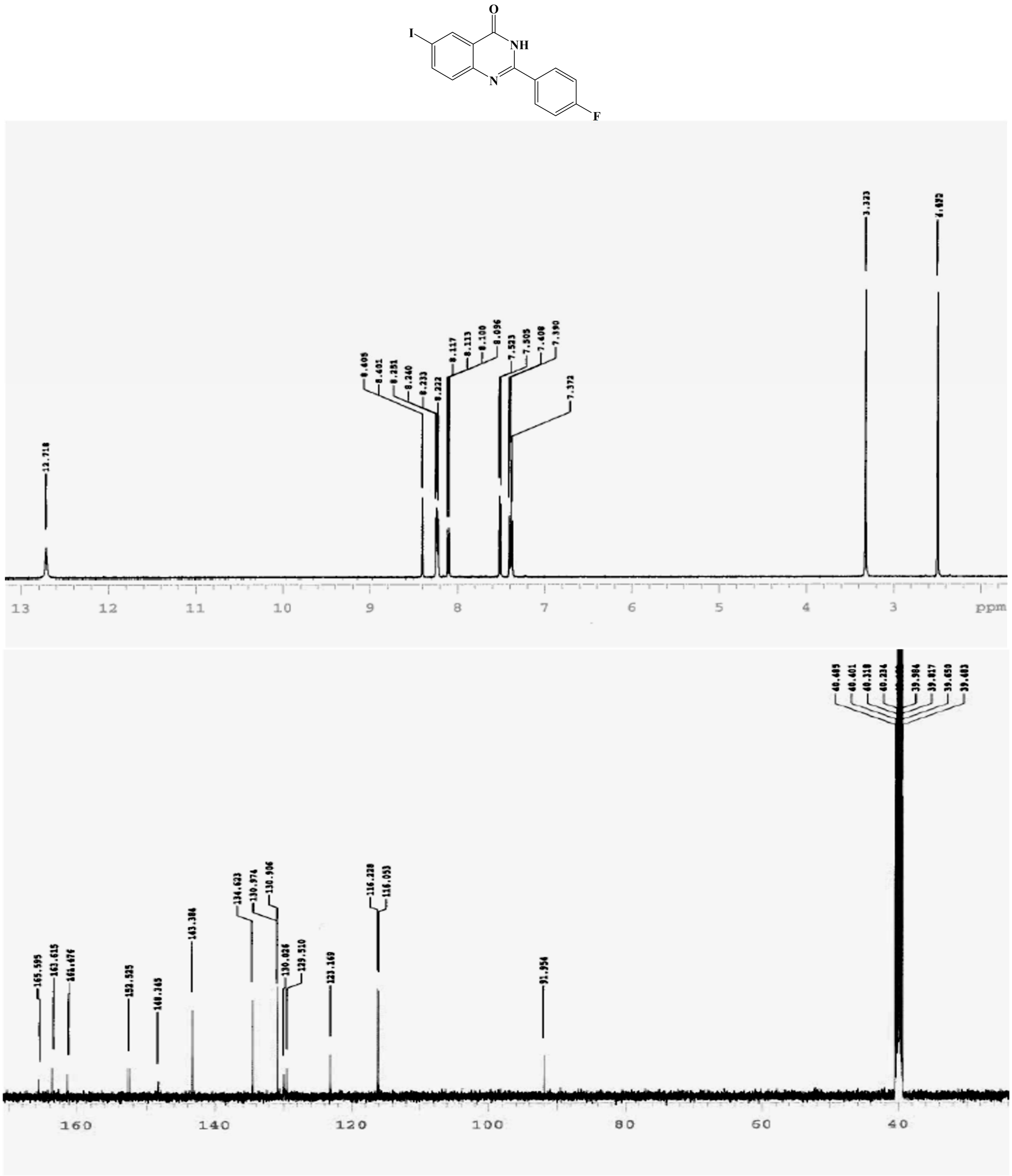

^{1}H-NMR and ^{13}C-NMR Spectra of **2b** (DMSO-d_6).

Figure S4. *Cont.*

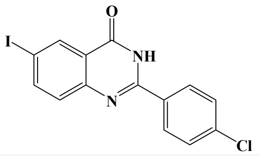

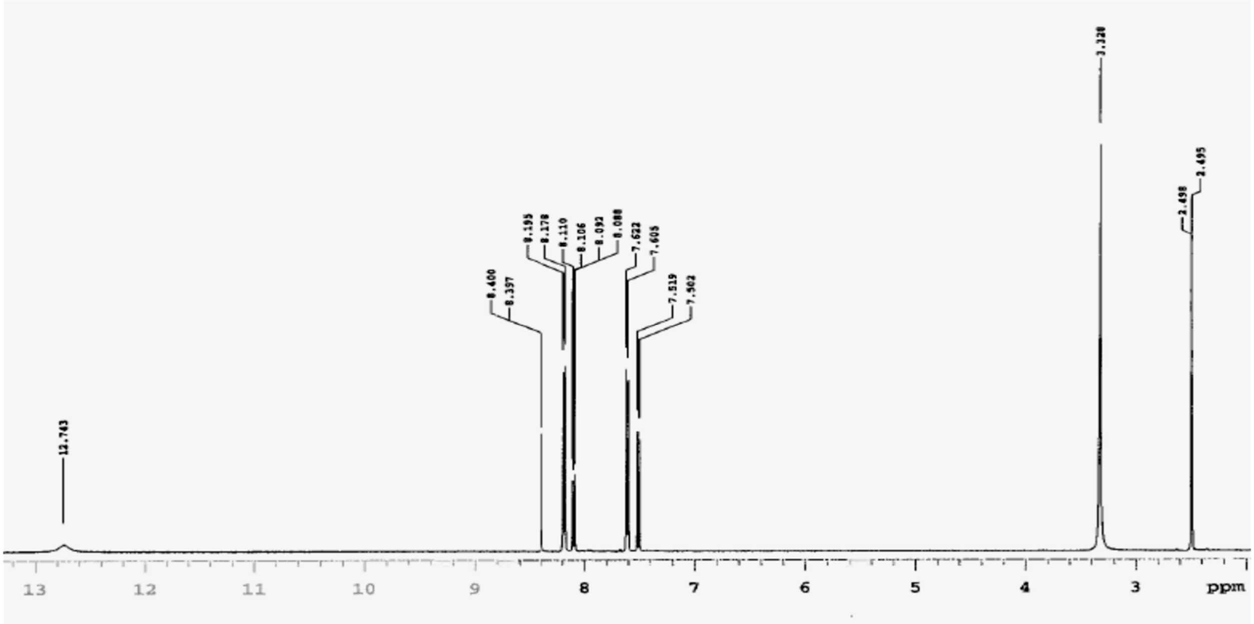

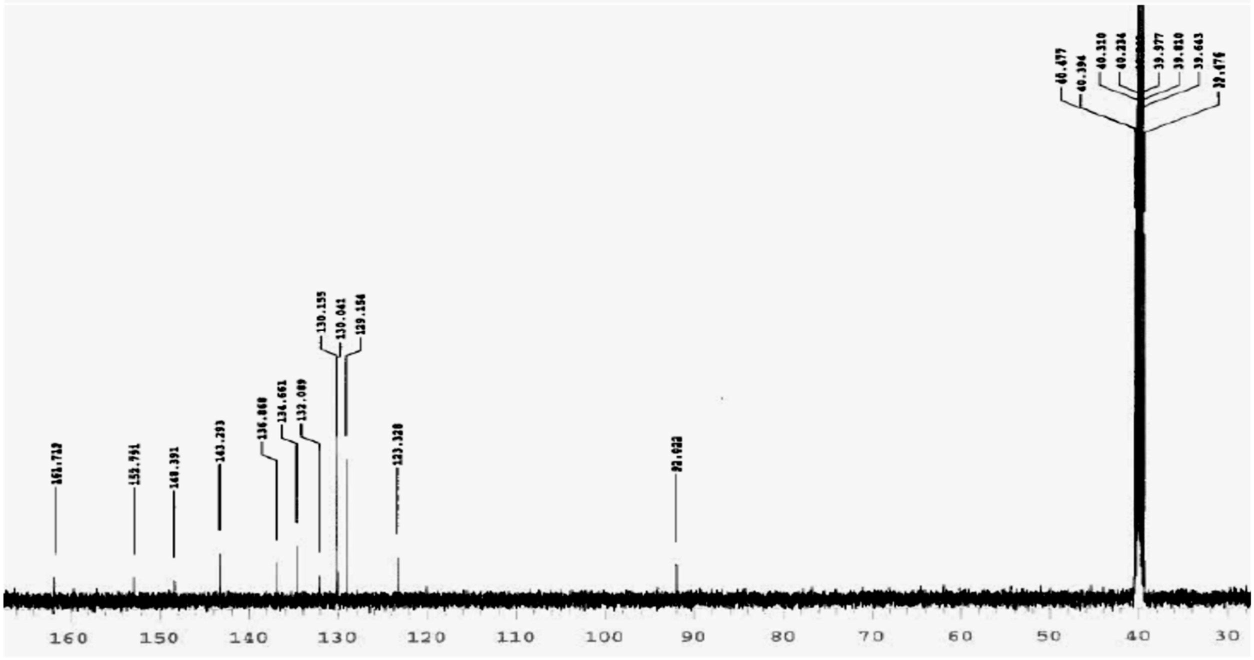

^{1}H-NMR and ^{13}C-NMR Spectra of **2c** (DMSO-d_6).

Figure S4. *Cont.*

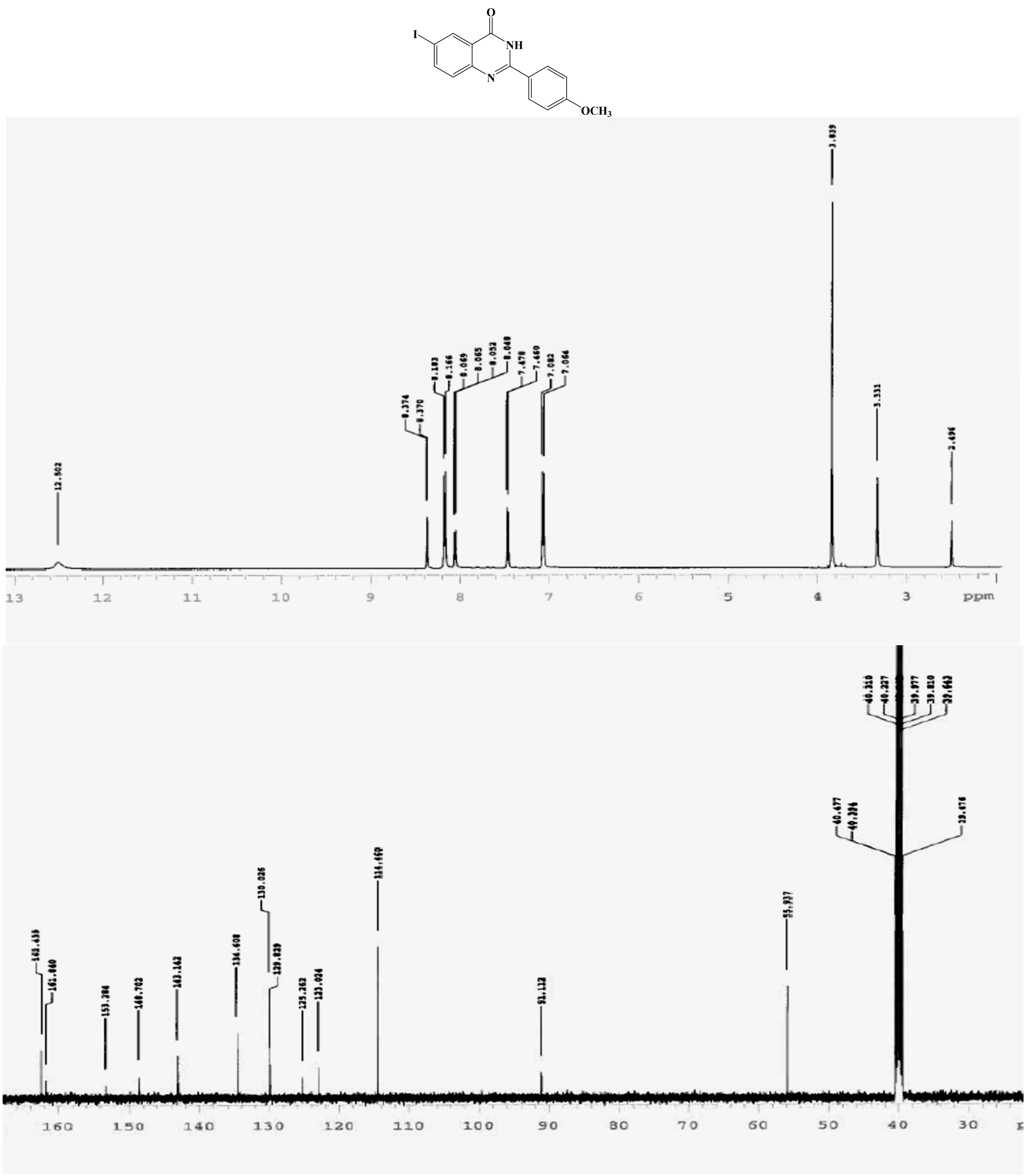

^{1}H-NMR and ^{13}C-NMR Spectra of **2d** (DMSO-*d*$_6$).

Figure S4. *Cont.*

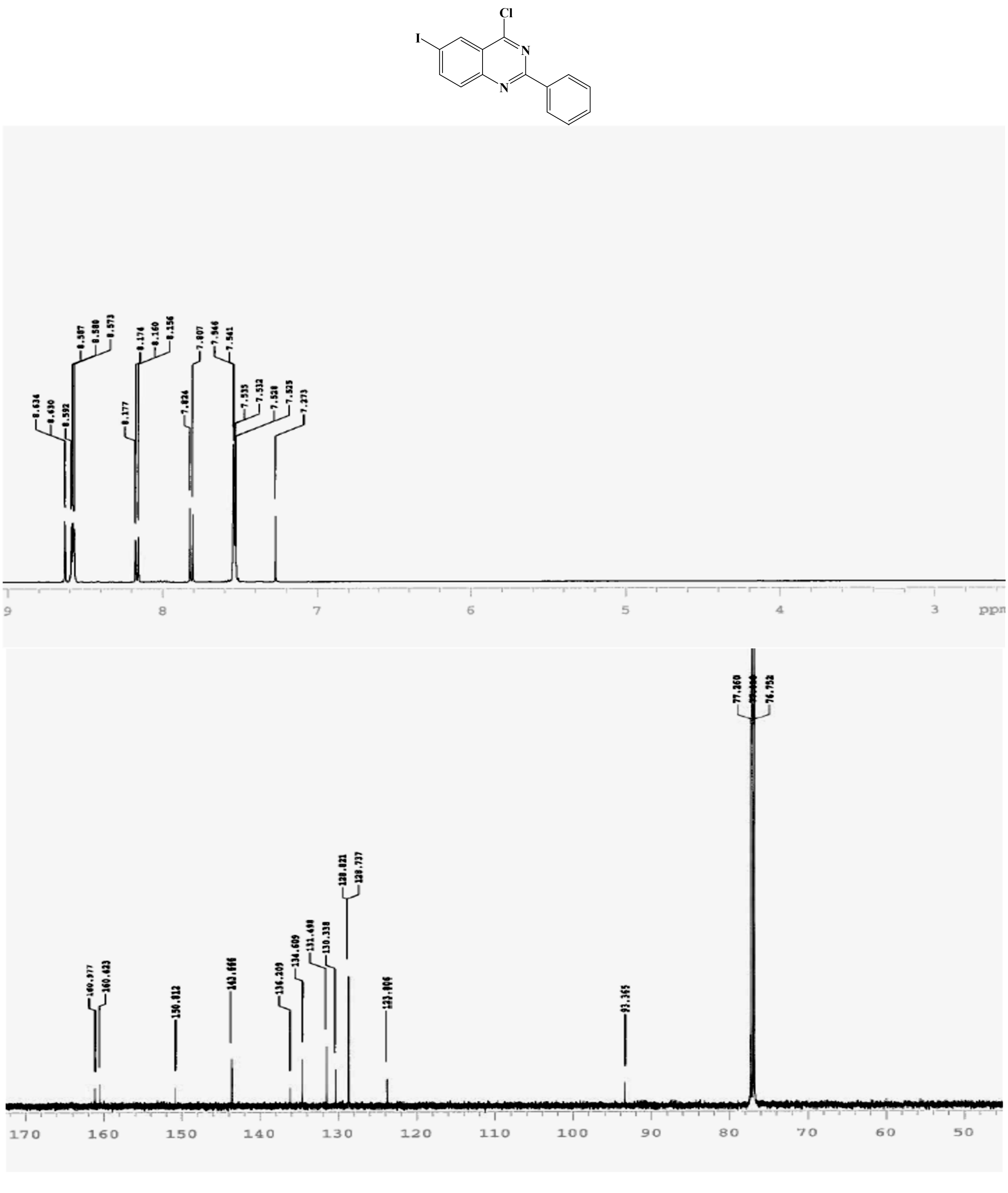

^{1}H-NMR and ^{13}C-NMR Spectra of **3a** ($CDCl_3$).

Figure S4. *Cont.*

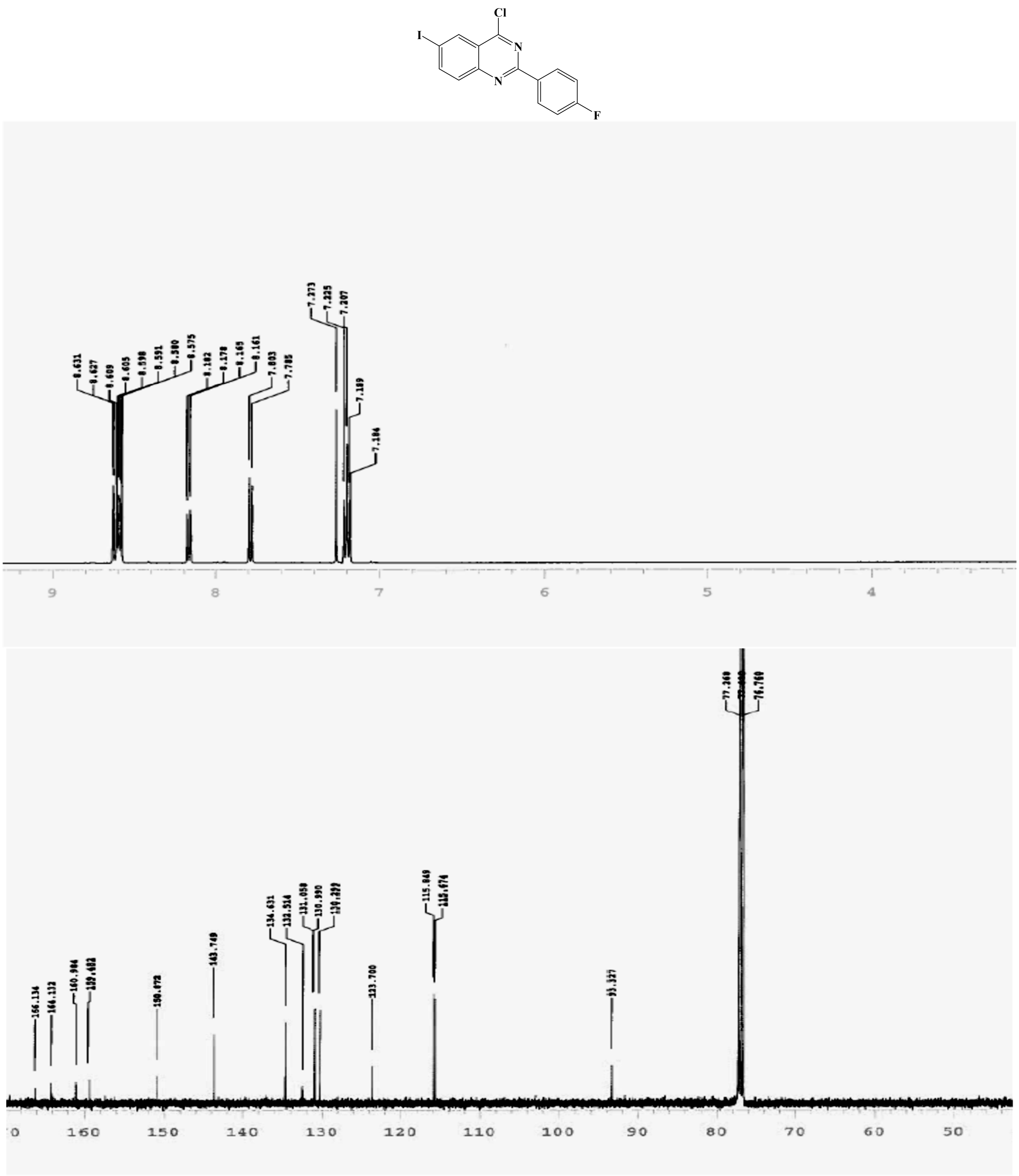

^{1}H-NMR and ^{13}C-NMR Spectra of **3b** ($CDCl_3$).

Figure S4. *Cont.*

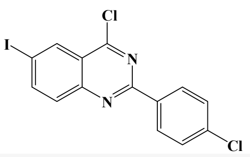

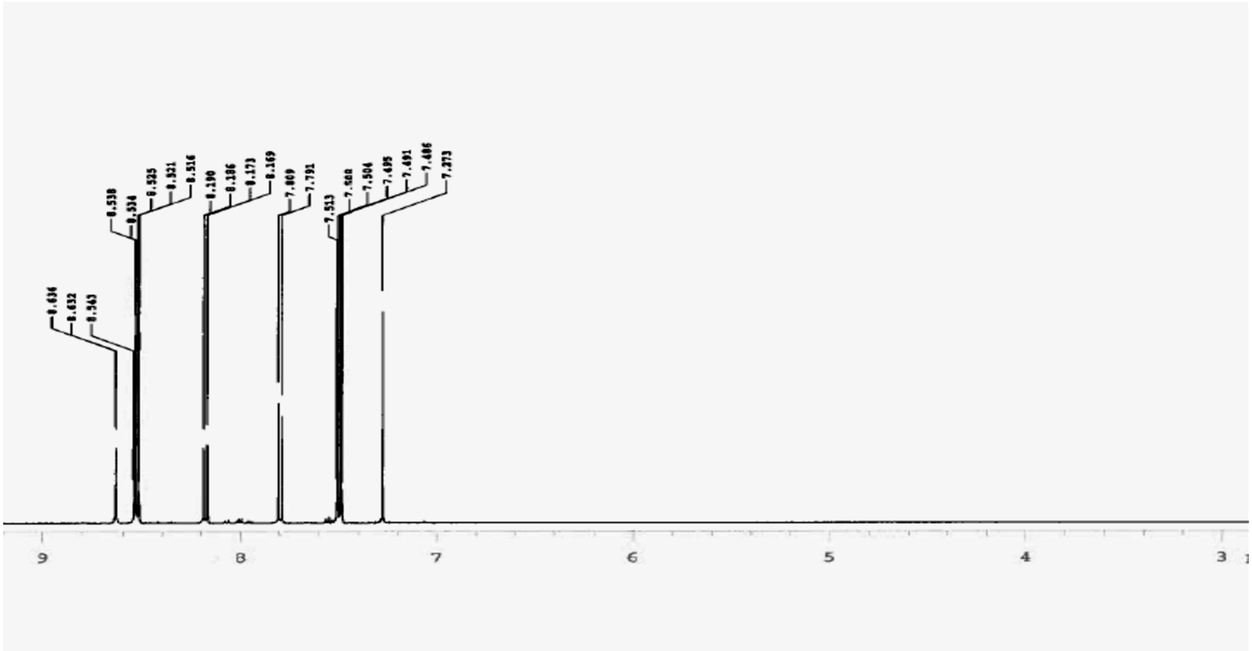

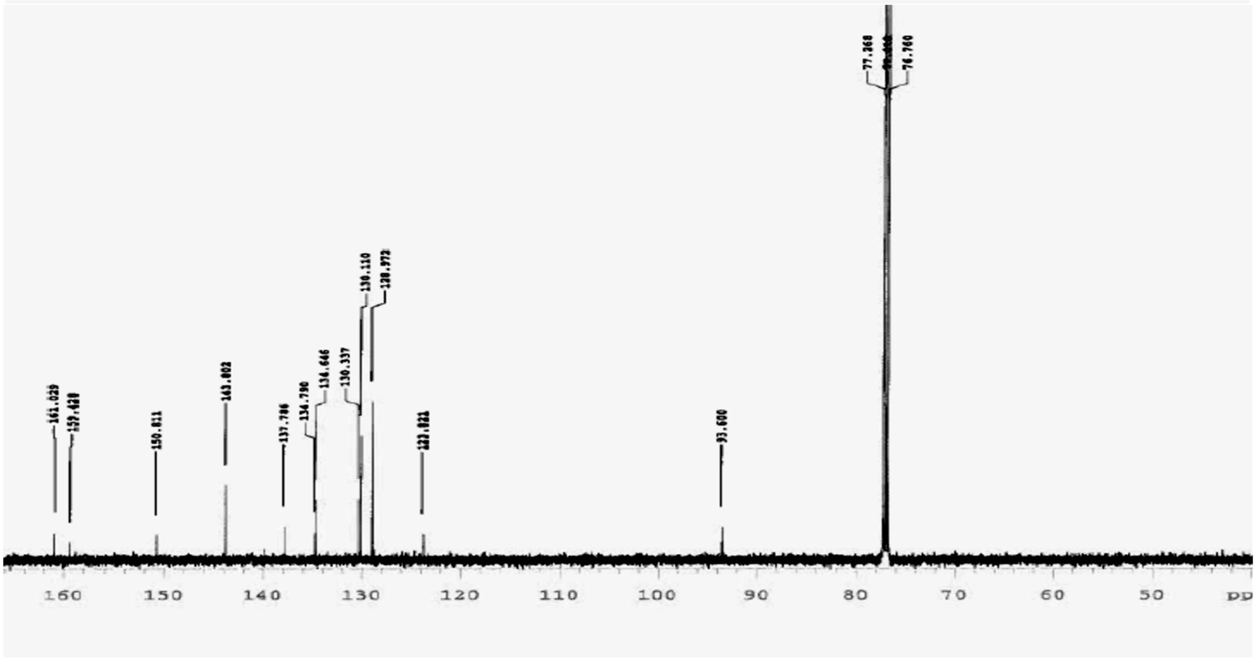

^{1}H-NMR and ^{13}C-NMR Spectra of **3c** ($CDCl_3$).

Figure S4. *Cont.*

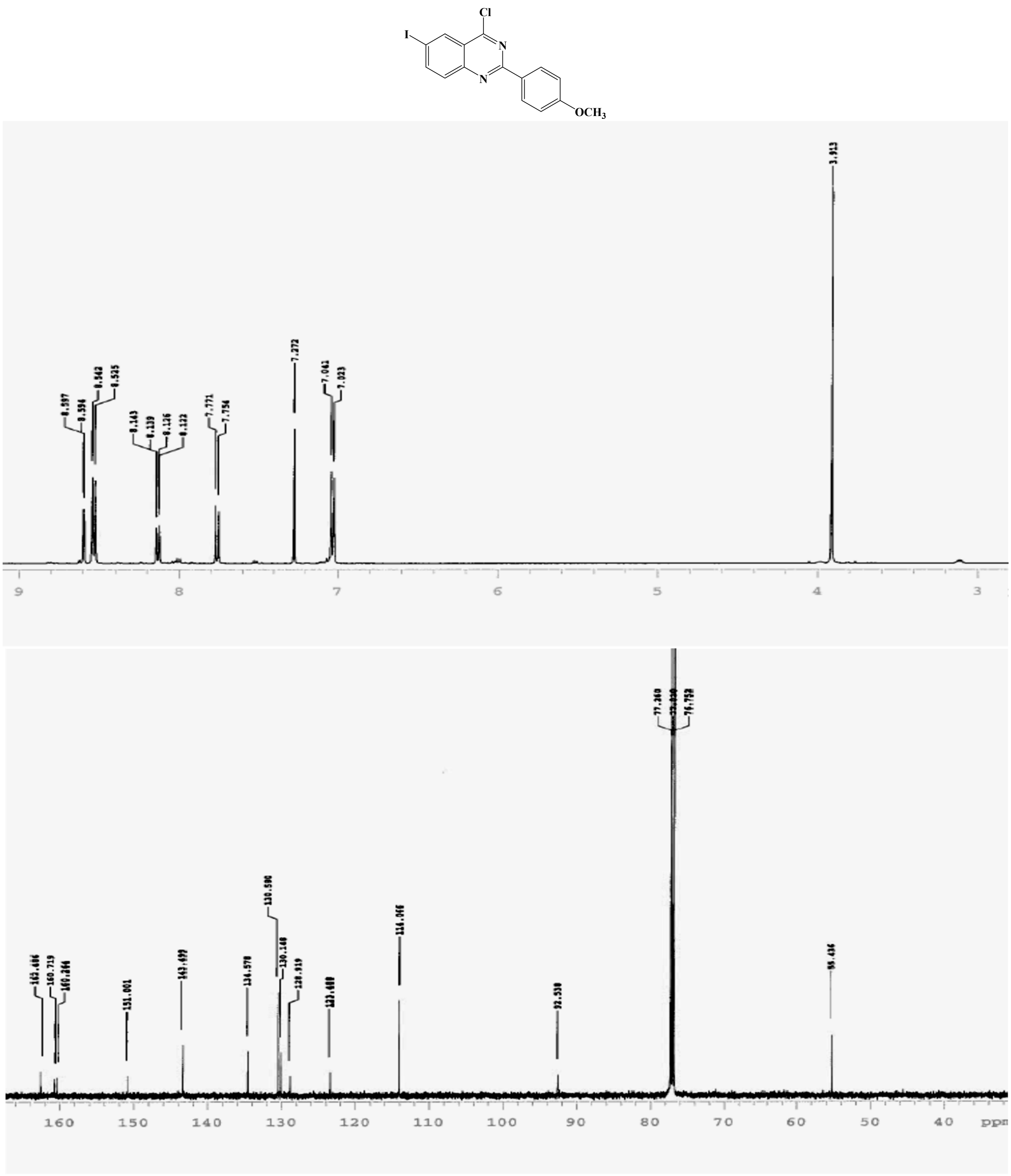

^{1}H-NMR and ^{13}C-NMR Spectra of **3d** ($CDCl_3$).

Figure S4. *Cont.*

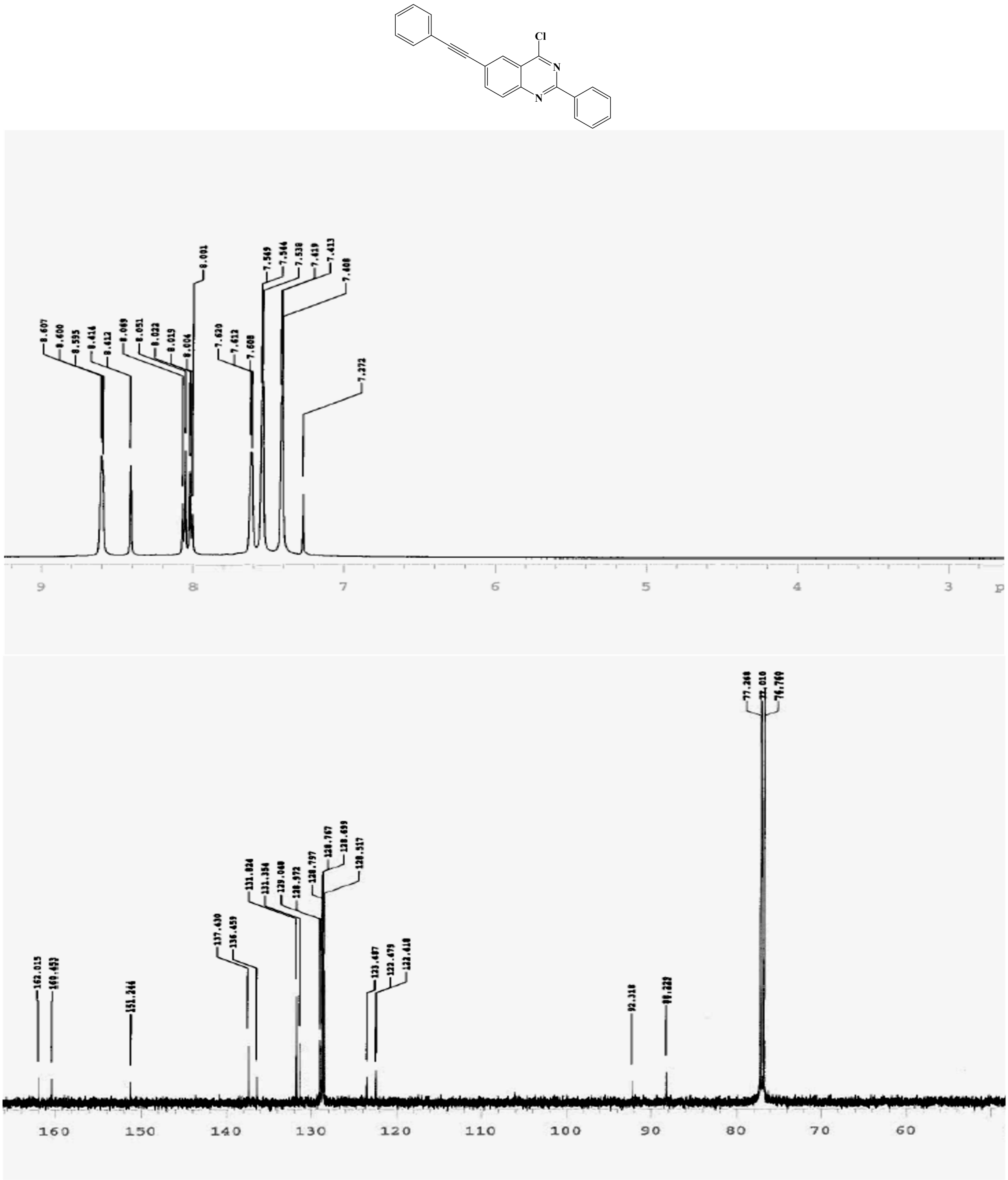

^{1}H-NMR and ^{13}C-NMR Spectra of **4a** ($CDCl_3$).

Figure S4. *Cont.*

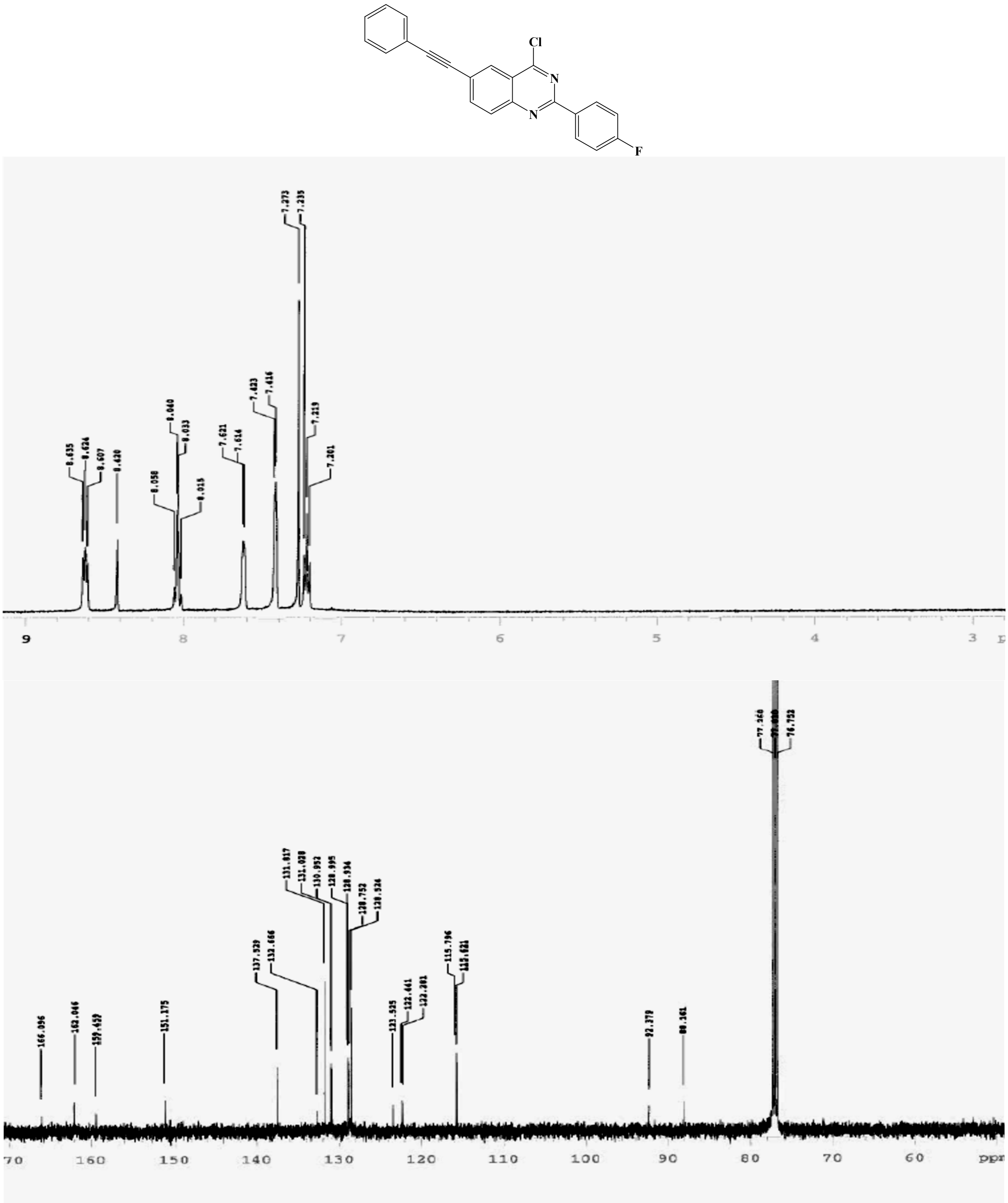

^{1}H-NMR and ^{13}C-NMR Spectra of **4b** ($CDCl_3$).

Figure S4. *Cont.*

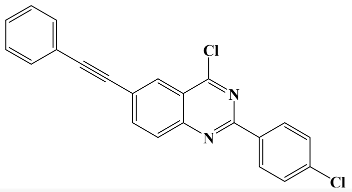

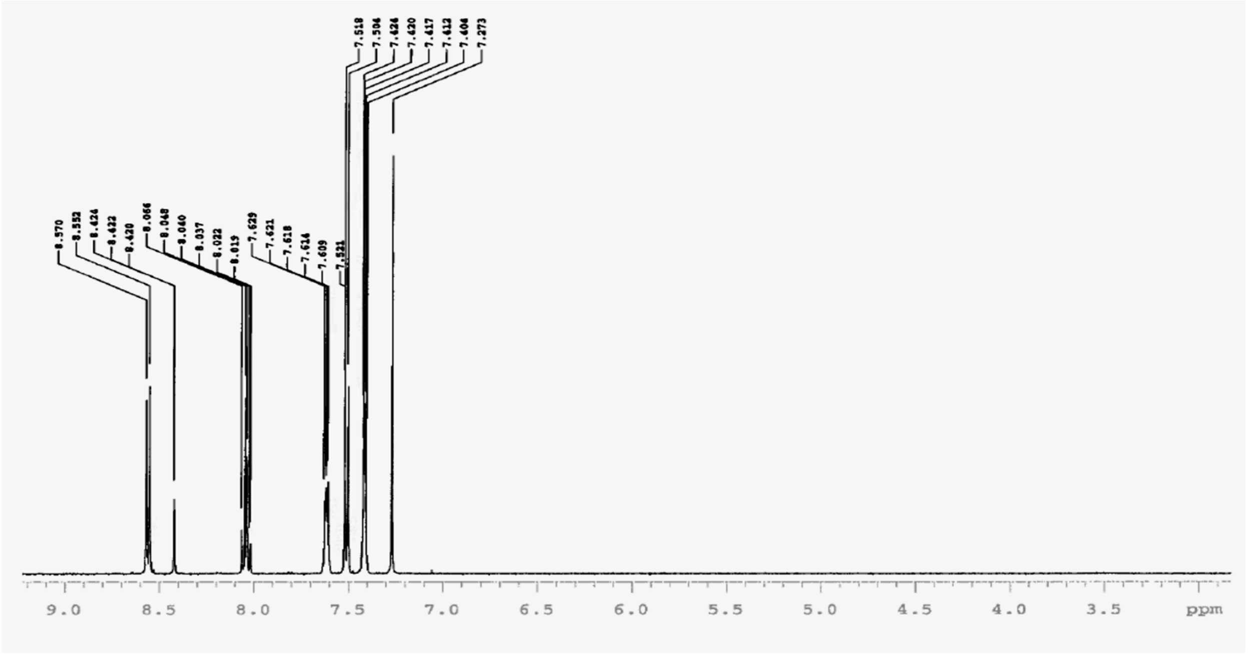

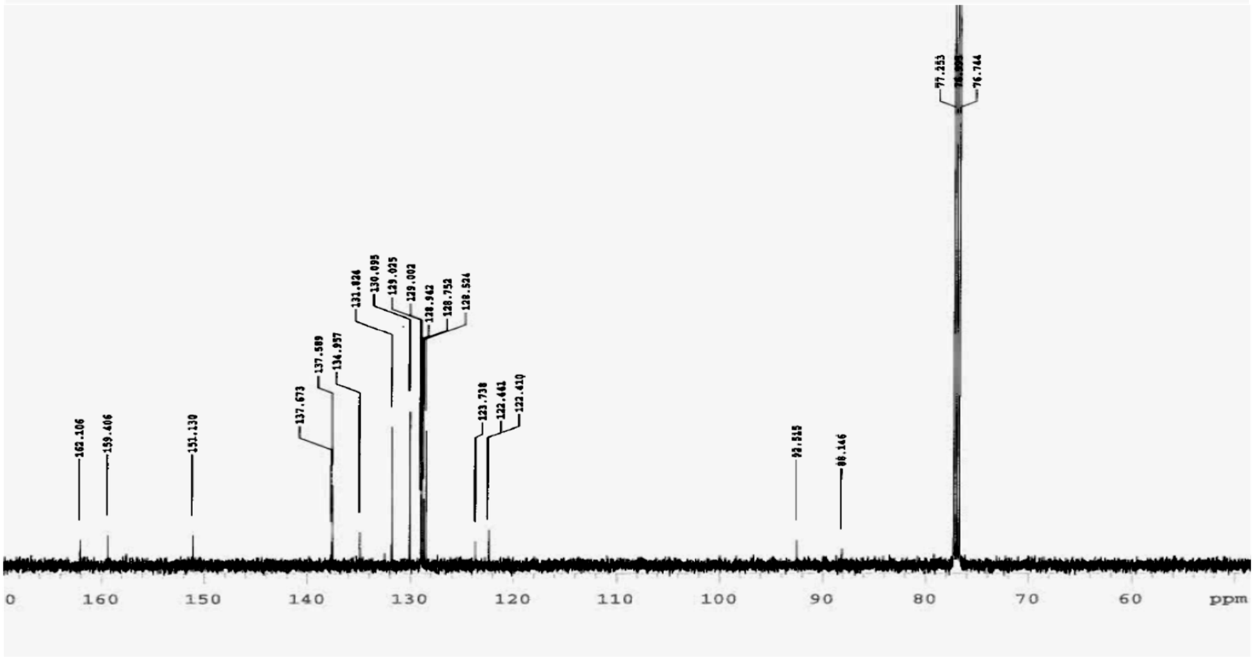

^{1}H-NMR and ^{13}C-NMR Spectra of **4c** ($CDCl_3$).

Figure S4. *Cont.*

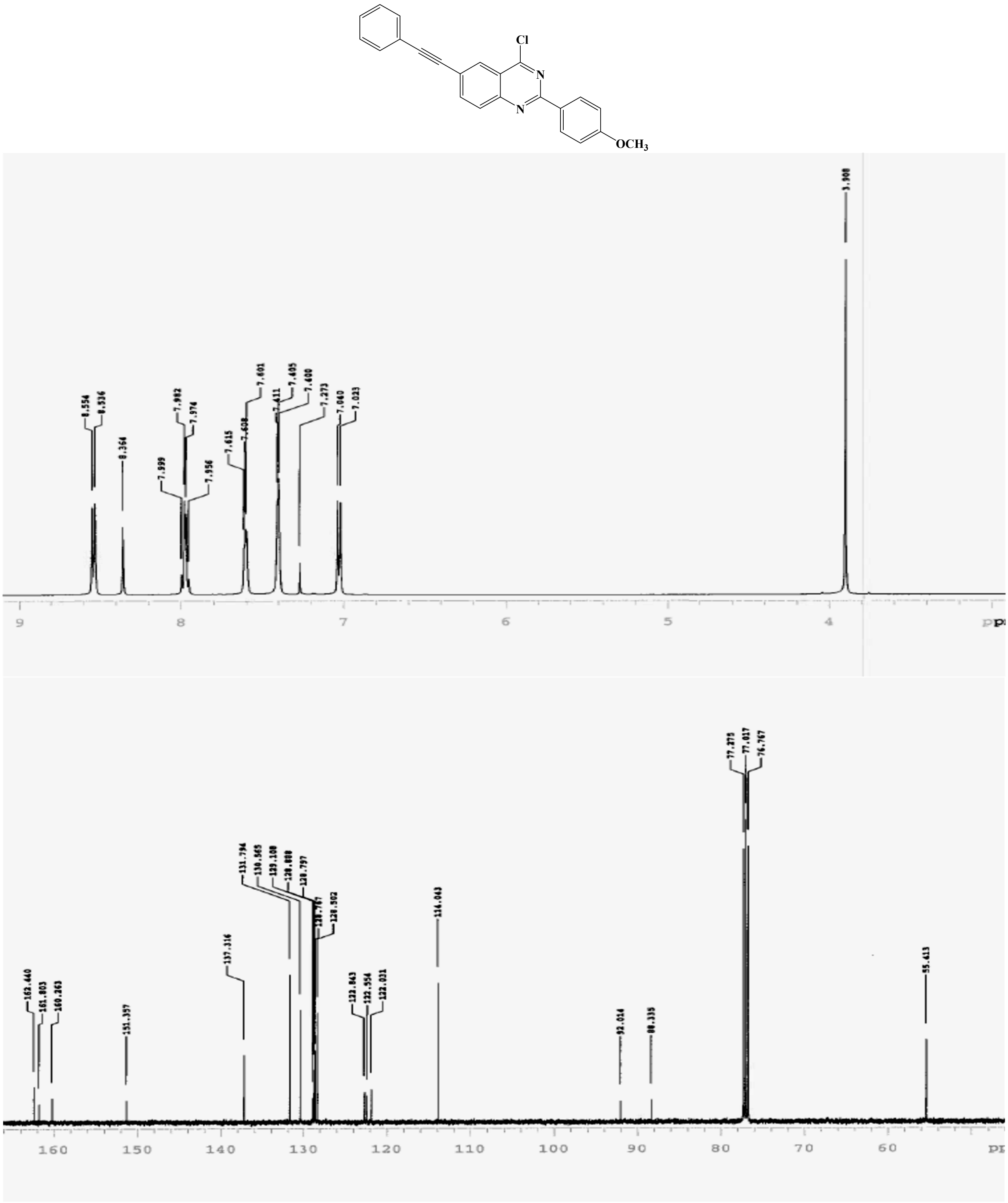

^{1}H-NMR and ^{13}C-NMR Spectra of **4d** ($CDCl_3$).

Figure S4. *Cont.*

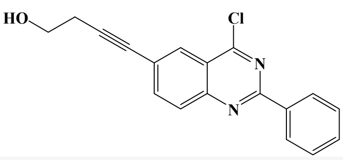

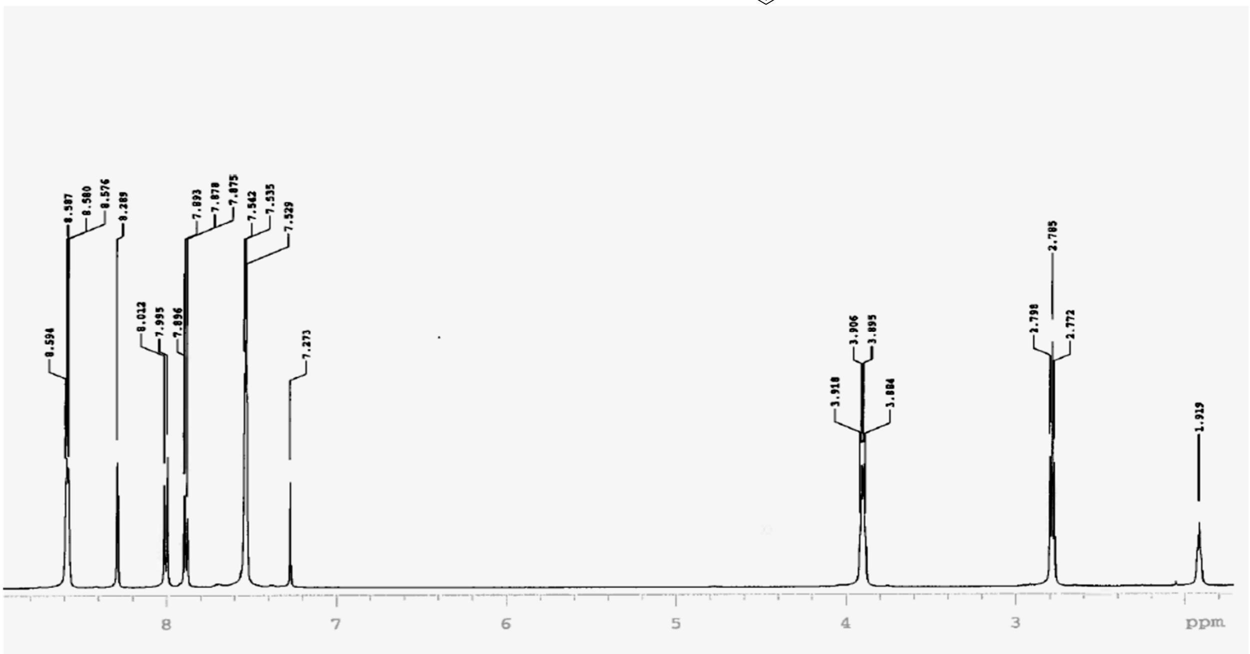

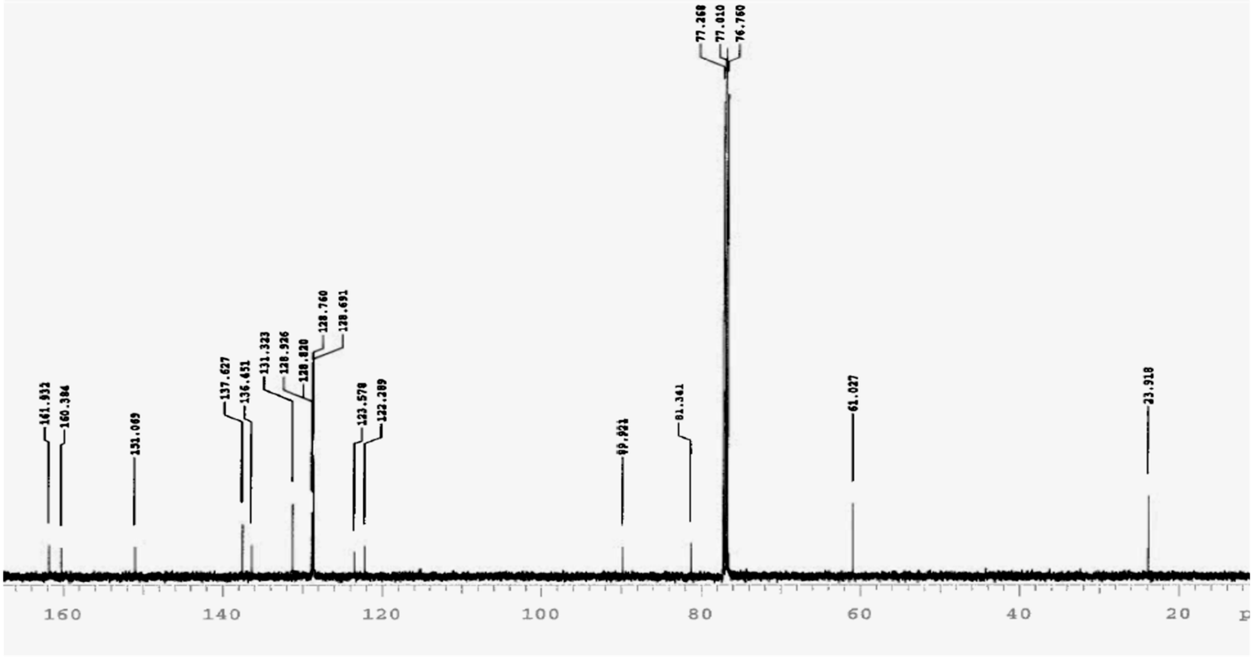

^{1}H-NMR and ^{13}C-NMR Spectra of **4e** ($CDCl_3$).

Figure S4. *Cont.*

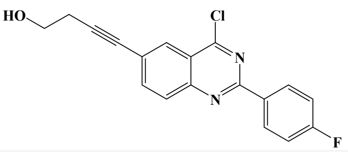

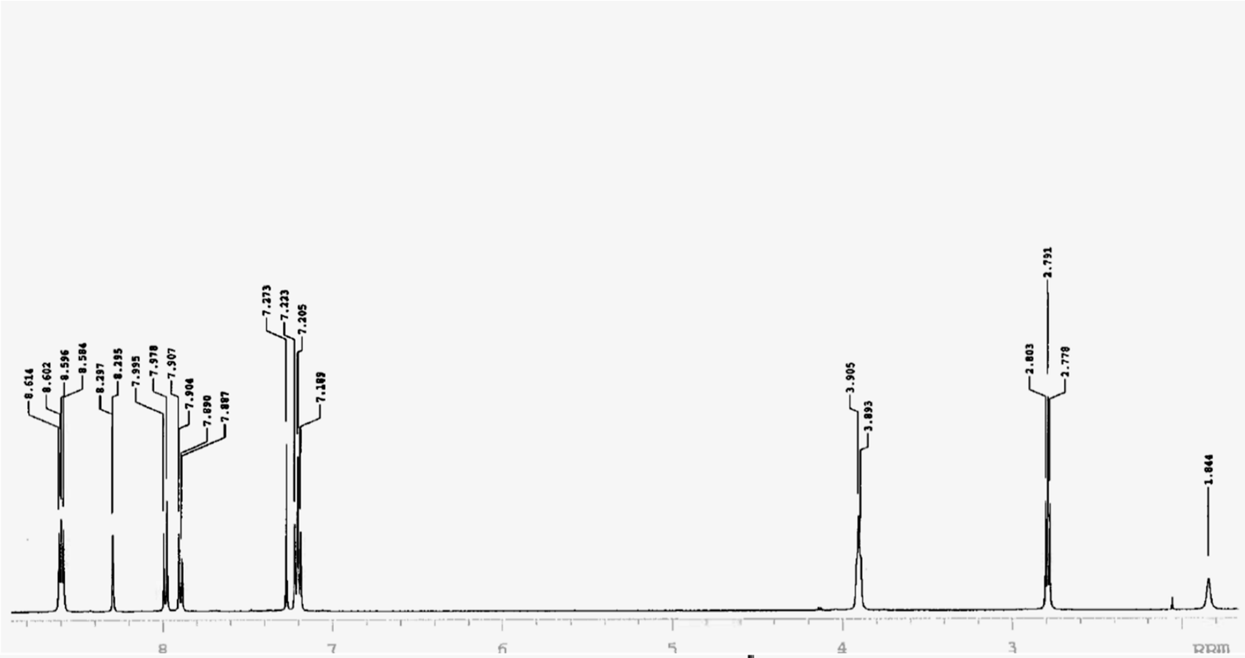

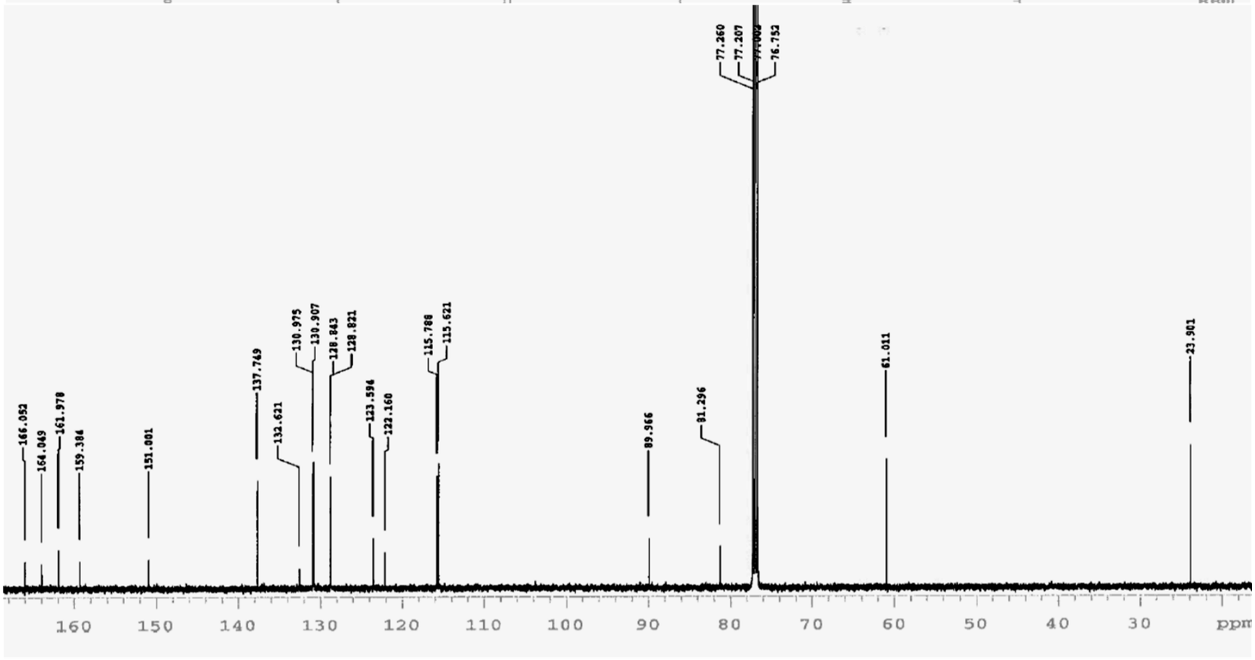

^{1}H-NMR and ^{13}C-NMR Spectra of **4f** ($CDCl_3$).

Figure S4. *Cont.*

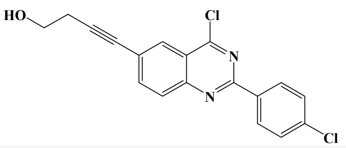

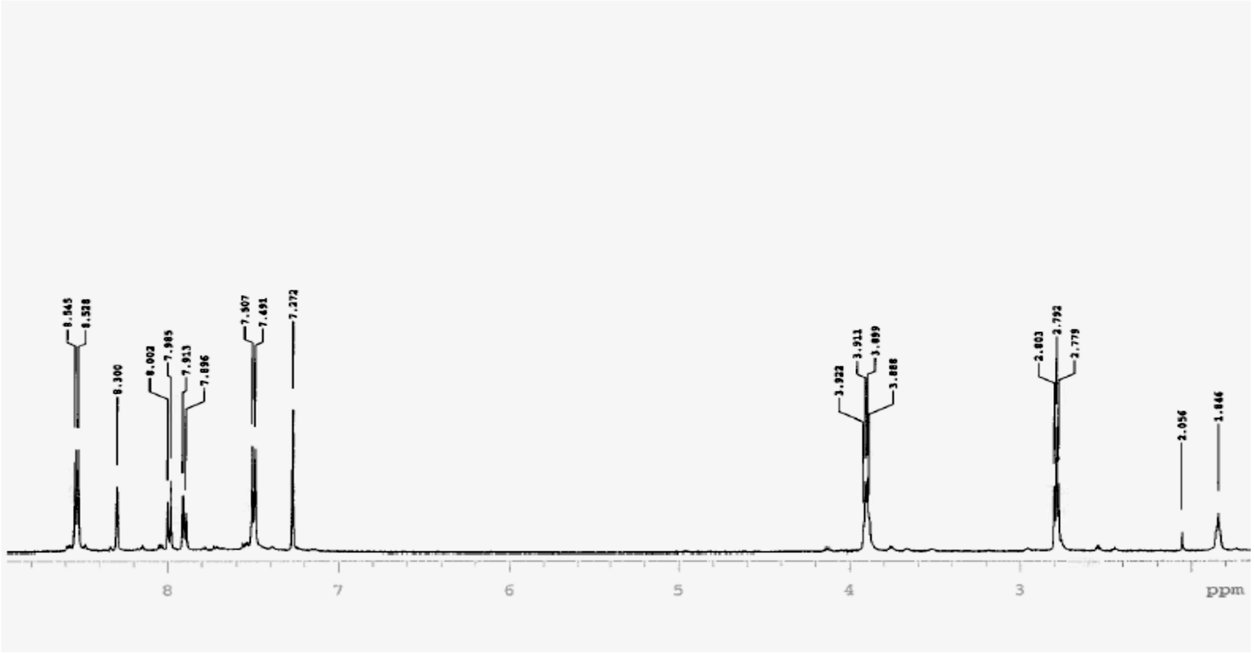

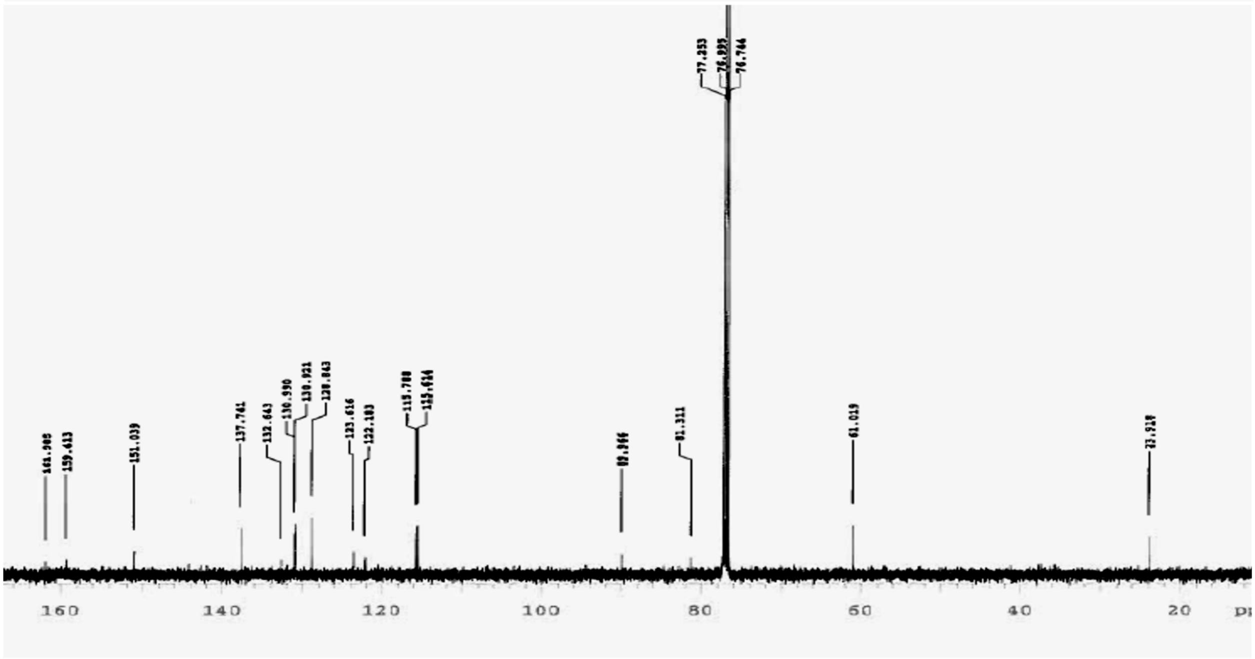

^{1}H-NMR and ^{13}C-NMR Spectra of **4g** ($CDCl_3$).

Figure S4. *Cont.*

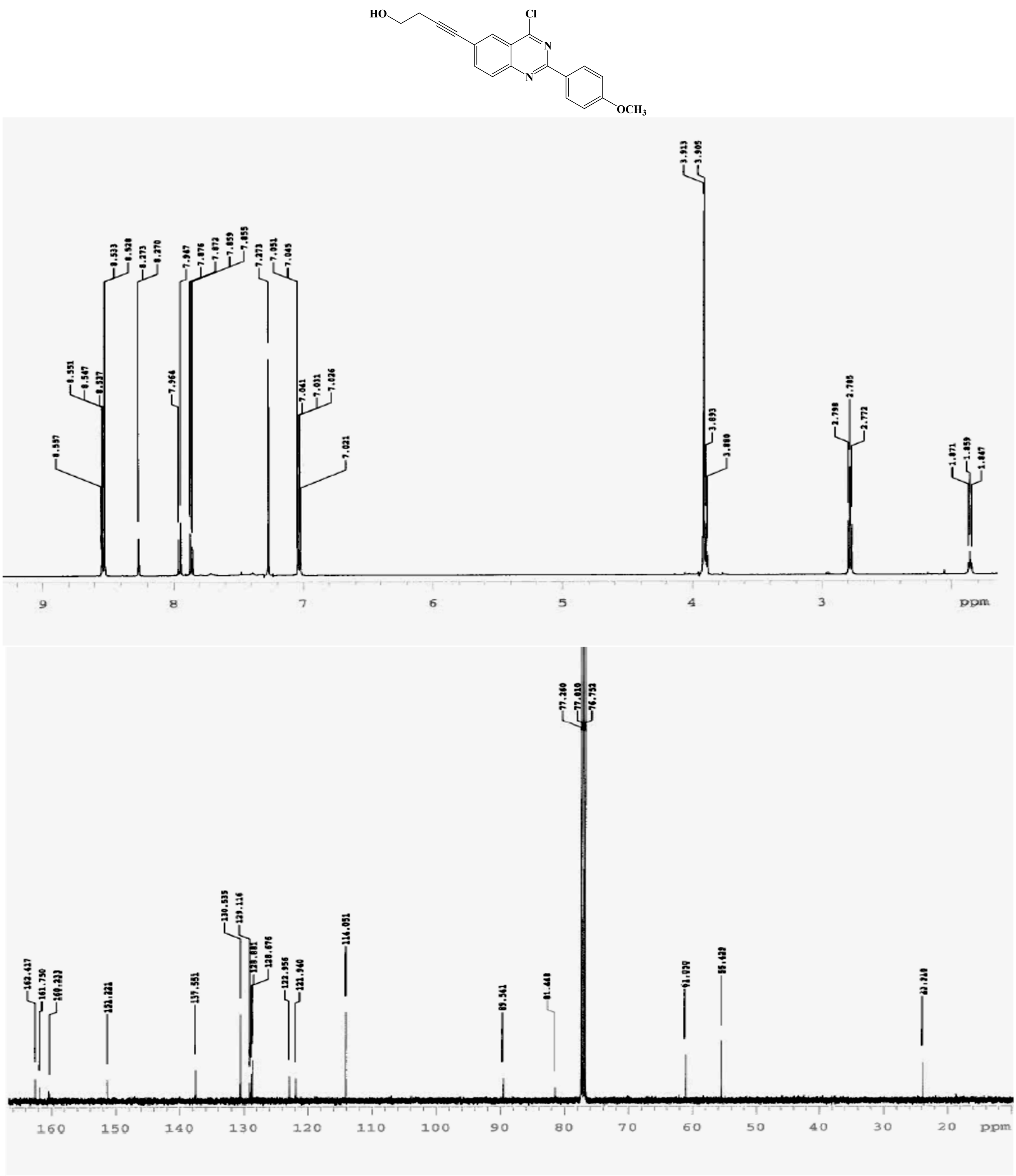

^{1}H-NMR and ^{13}C-NMR Spectra of **4h** ($CDCl_3$).

Figure S4. *Cont.*

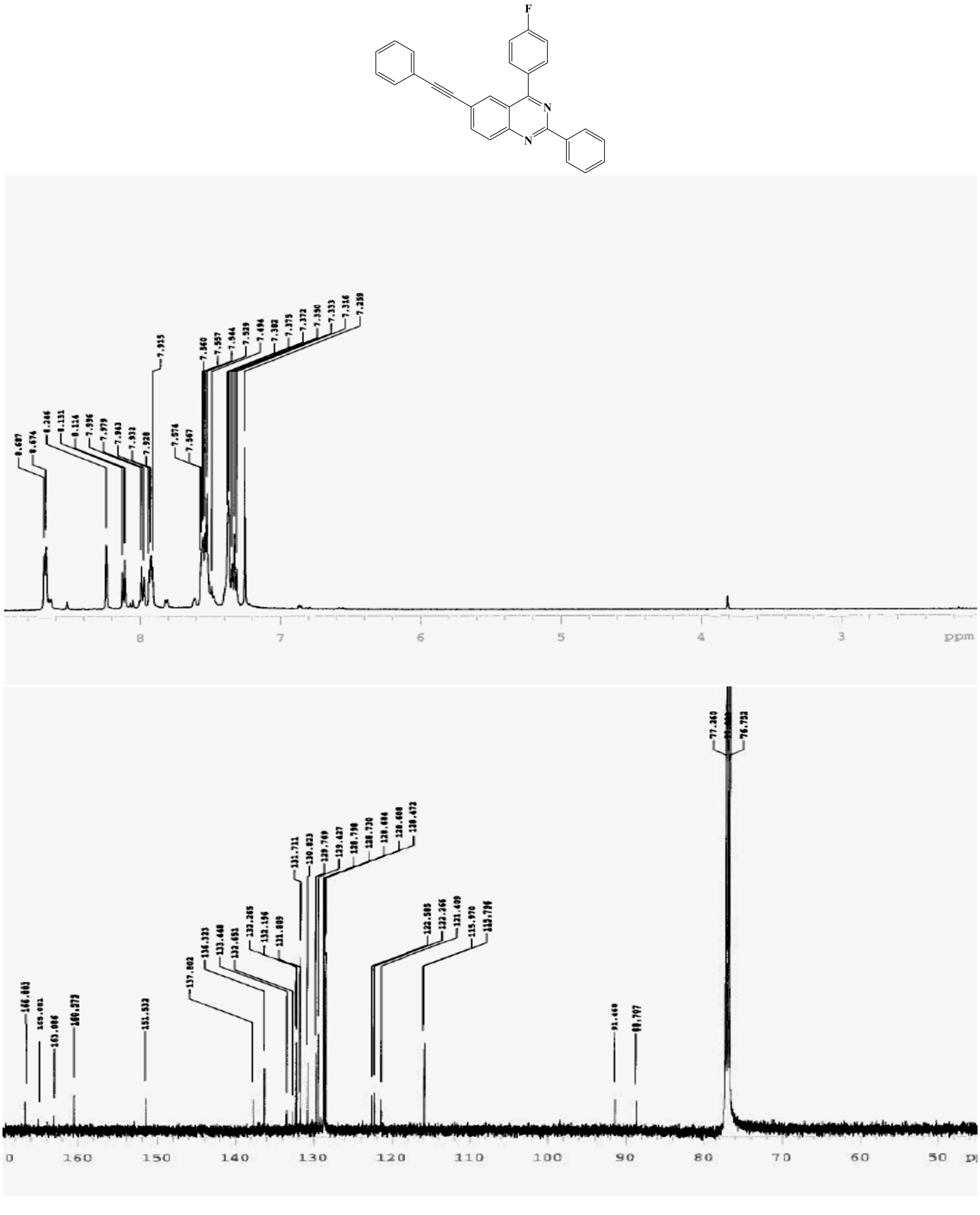

^{1}H-NMR and ^{13}C-NMR Spectra of **5a** ($CDCl_3$).

Figure S4. *Cont.*

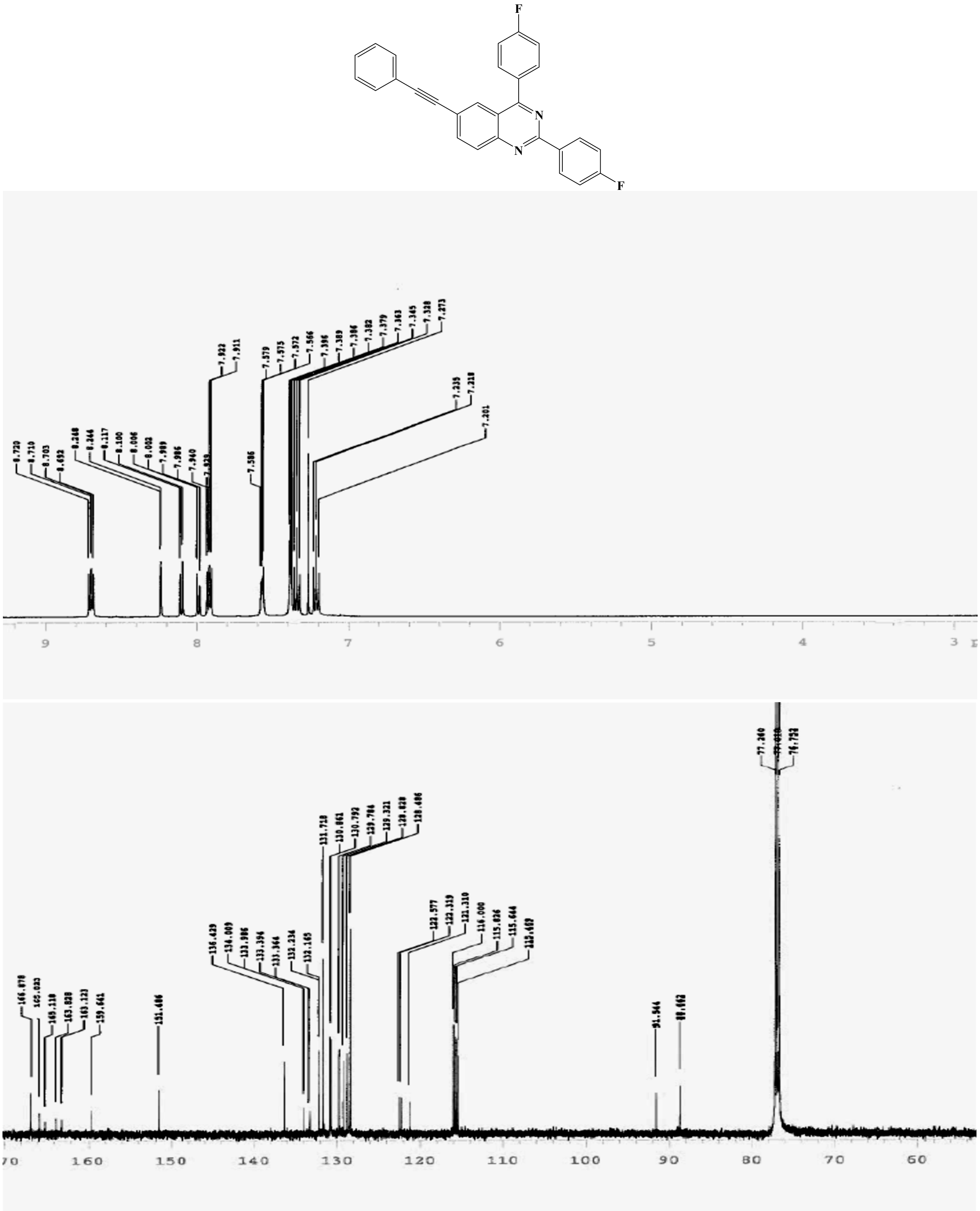

^{1}H-NMR and ^{13}C-NMR Spectra of **5b** ($CDCl_3$).

Figure S4. *Cont.*

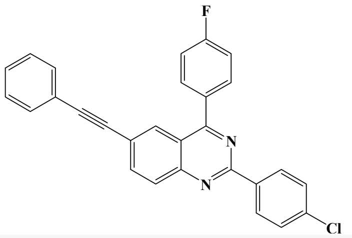

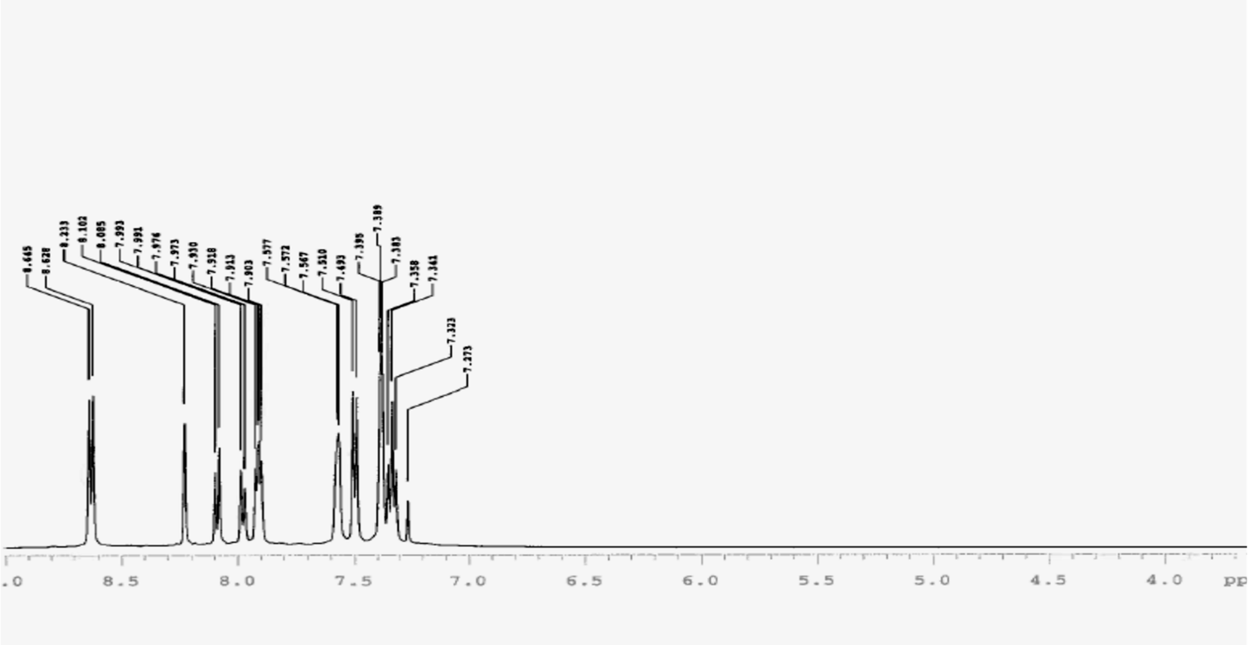

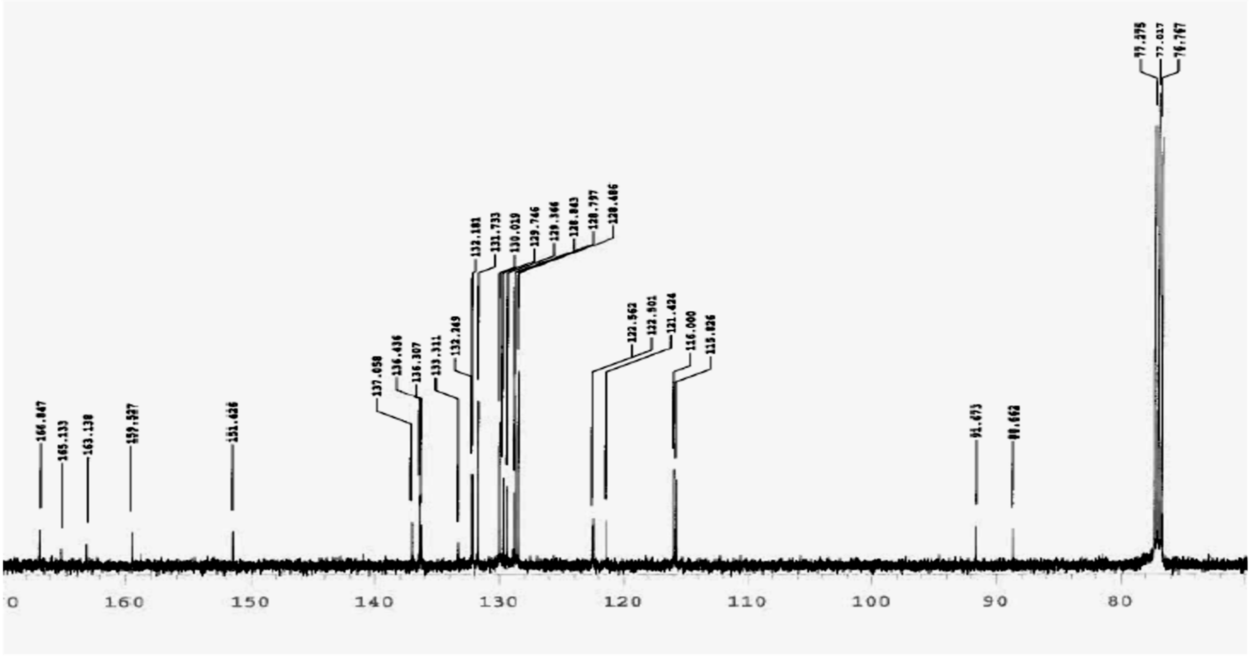

^{1}H-NMR and ^{13}C-NMR Spectra of **5c** ($CDCl_3$).

Figure S4. *Cont.*

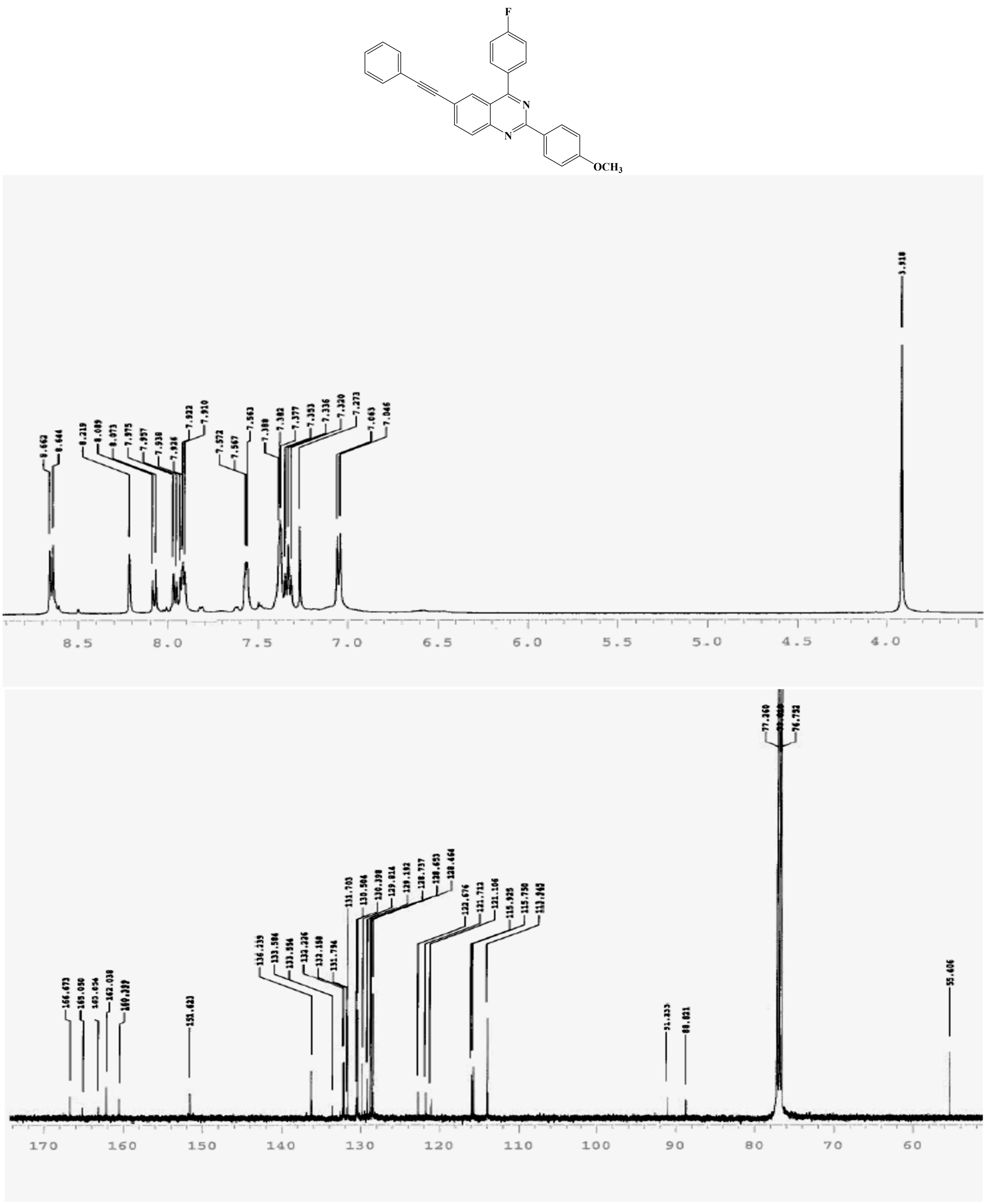

^{1}H-NMR and ^{13}C-NMR Spectra of **5d** ($CDCl_3$).

Figure S4. *Cont.*

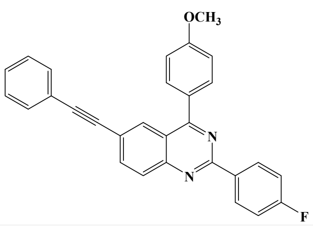

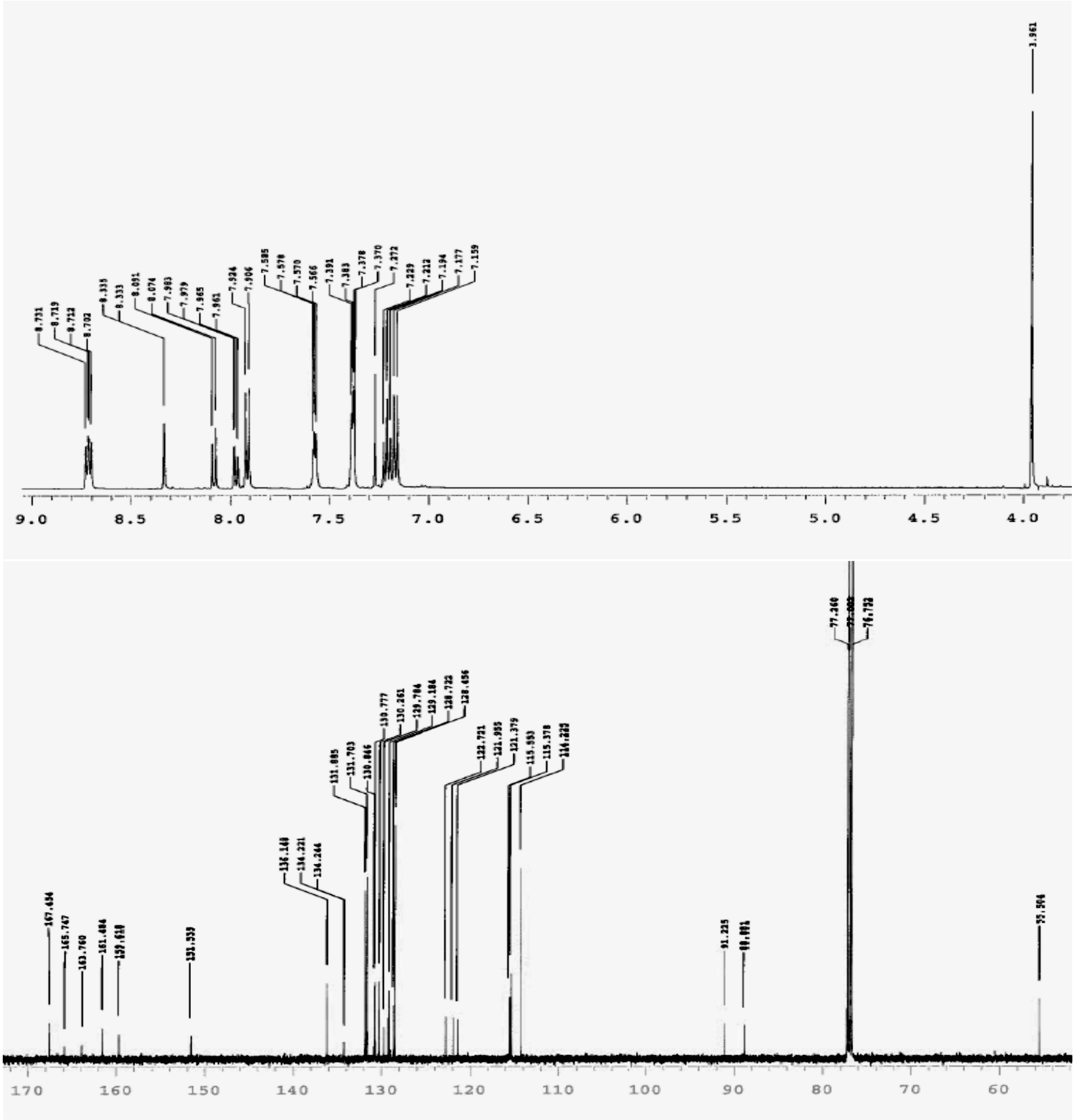

^{1}H-NMR and ^{13}C-NMR Spectra of **5e** ($CDCl_3$).

Figure S4. *Cont.*

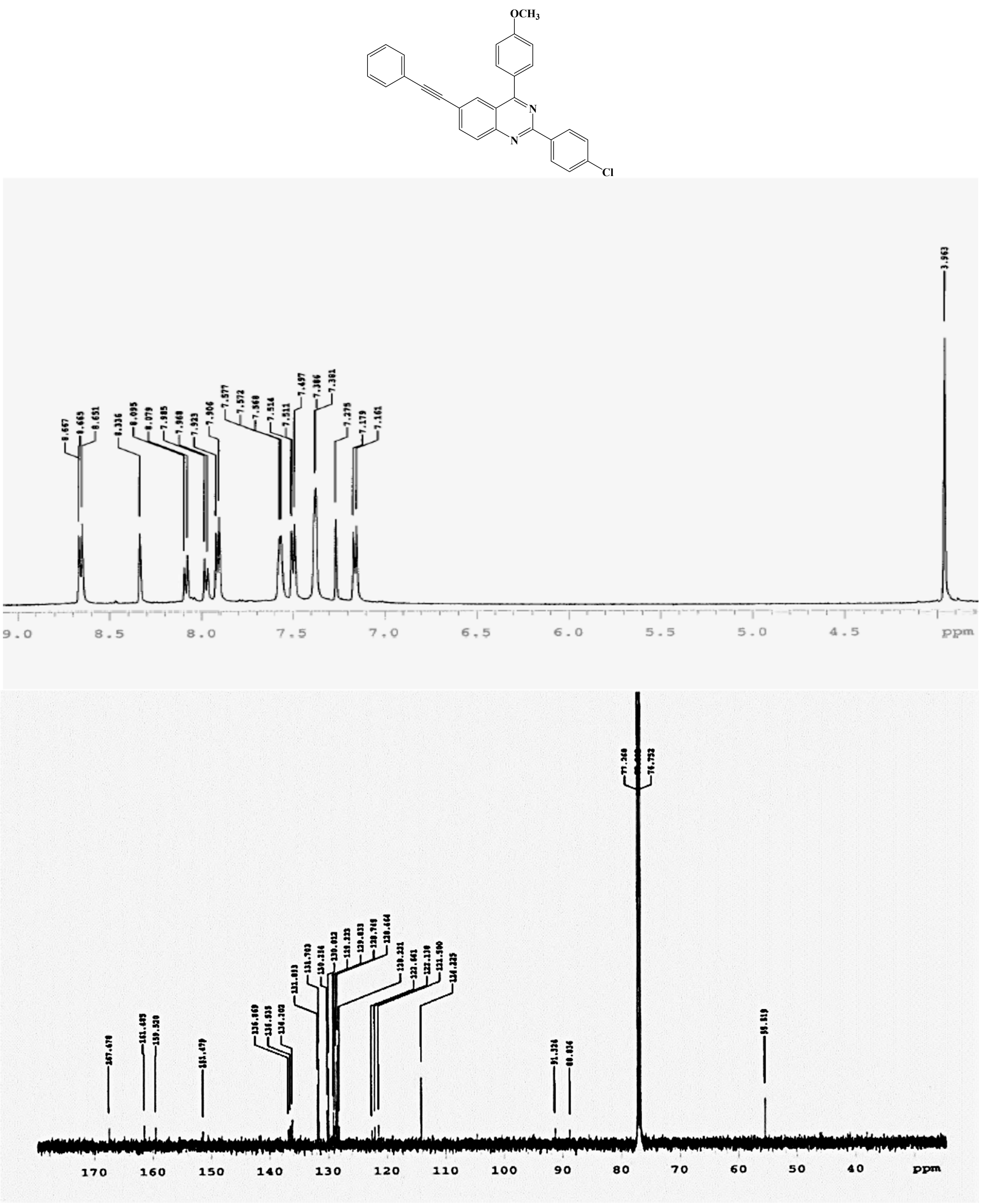

^{1}H-NMR and ^{13}C-NMR Spectra of **5f** ($CDCl_3$).

Figure S4. *Cont.*

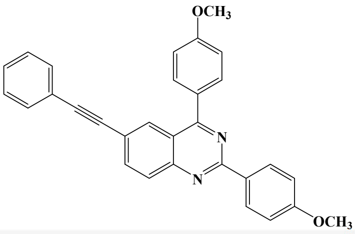

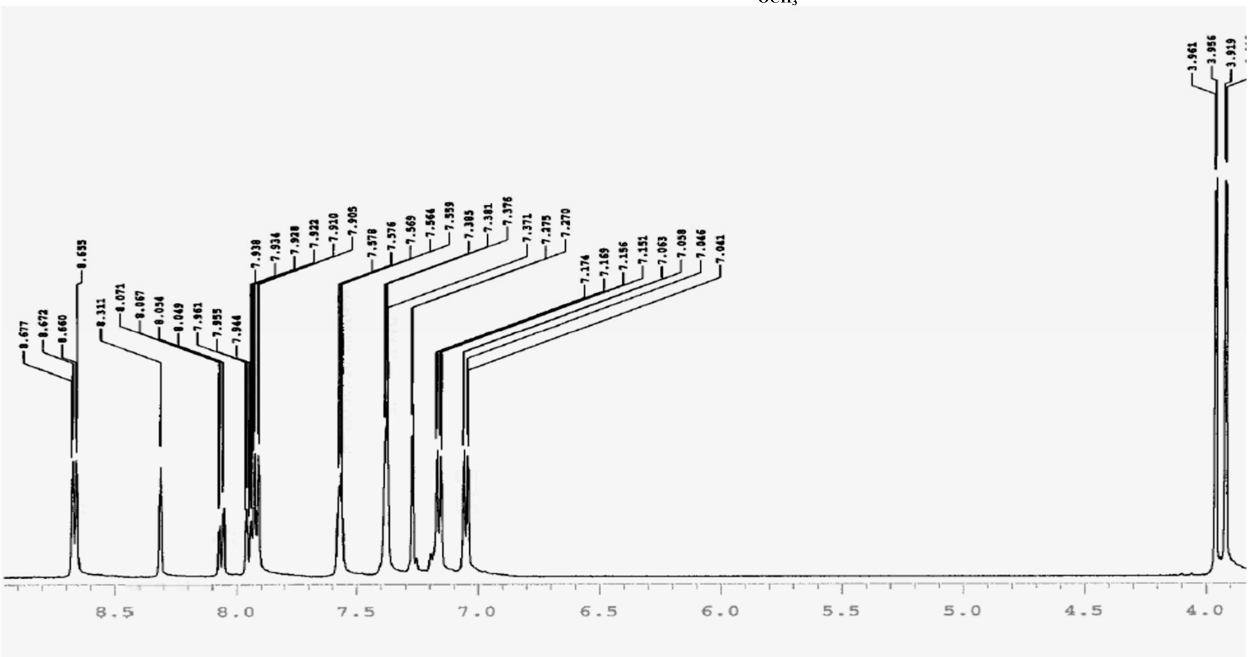

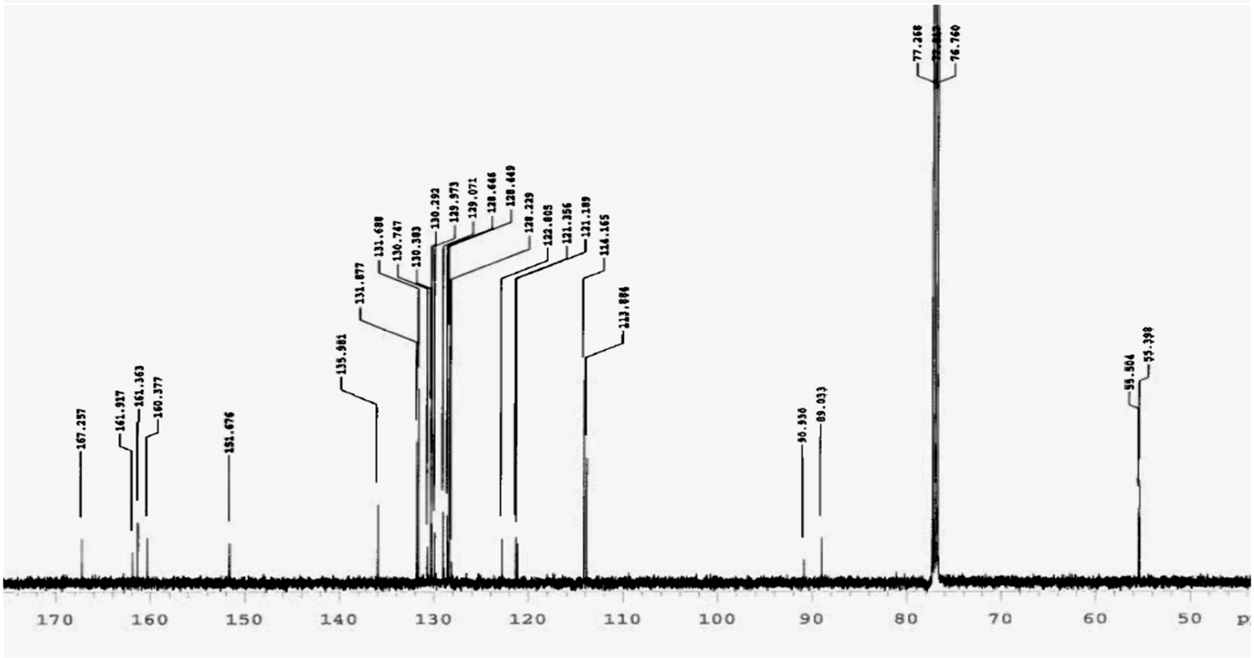

^{1}H-NMR and ^{13}C-NMR Spectra of **5g** ($CDCl_3$).

Figure S4. *Cont.*

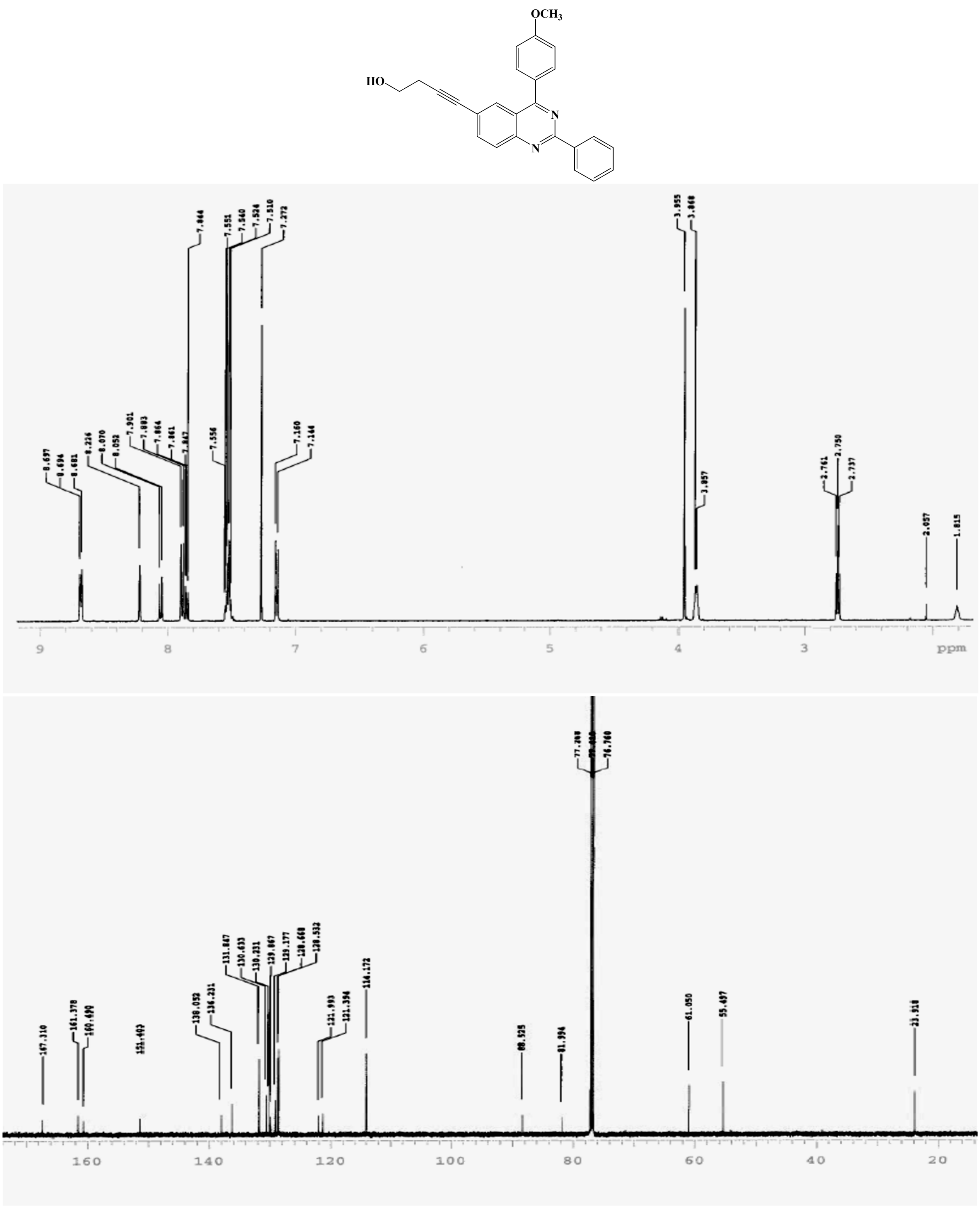

^{1}H-NMR and ^{13}C-NMR Spectra of **5h** ($CDCl_3$).

Figure S4. *Cont.*

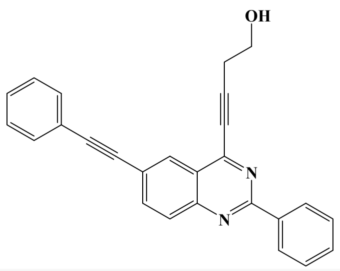

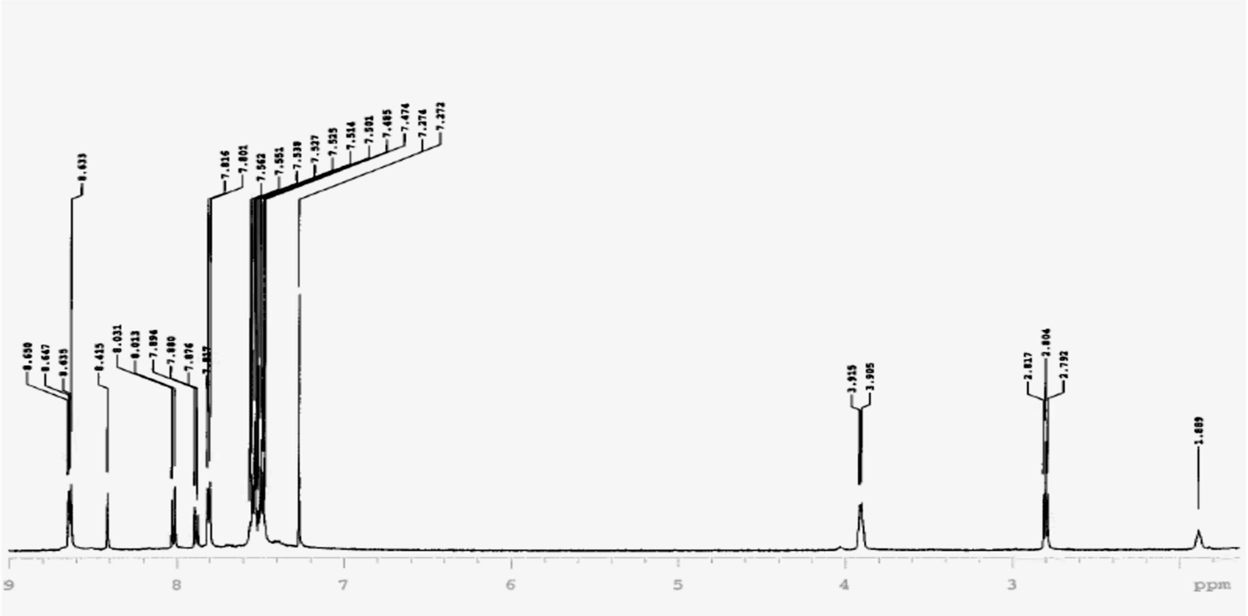

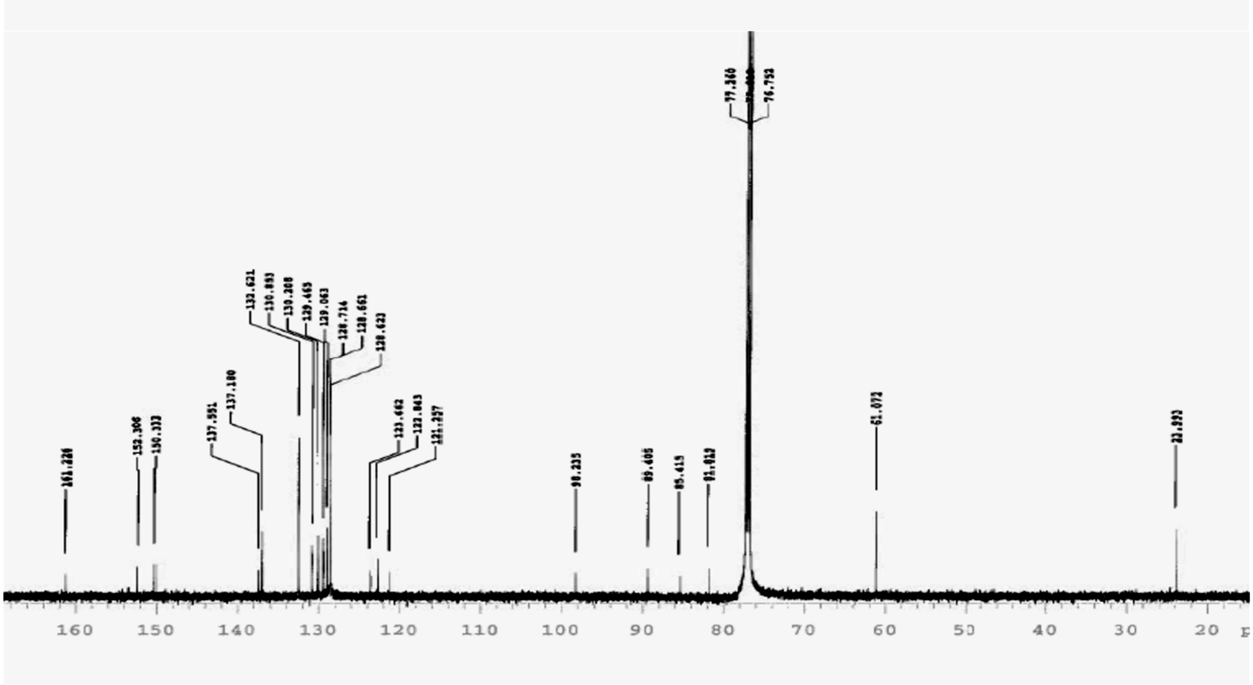

^{1}H-NMR and ^{13}C-NMR Spectra of **6a** ($CDCl_3$).

Figure S4. *Cont.*

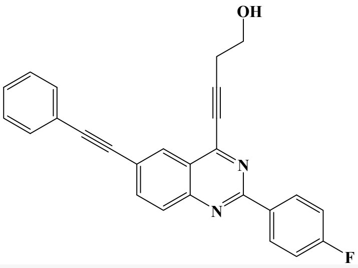

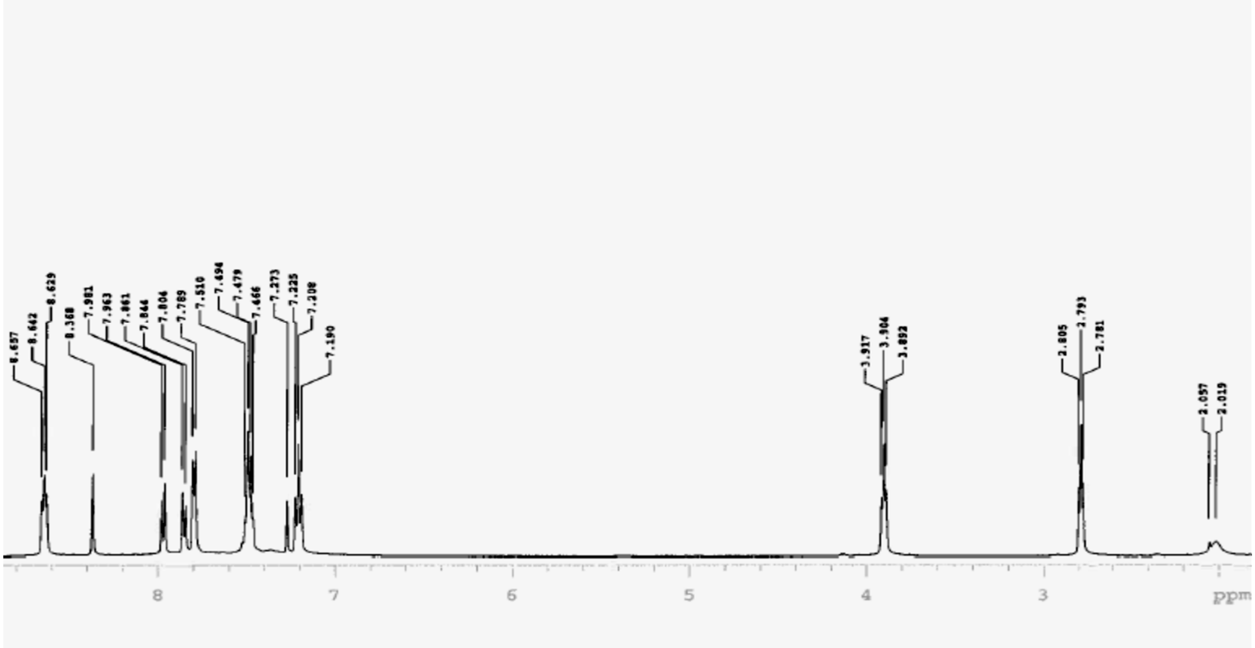

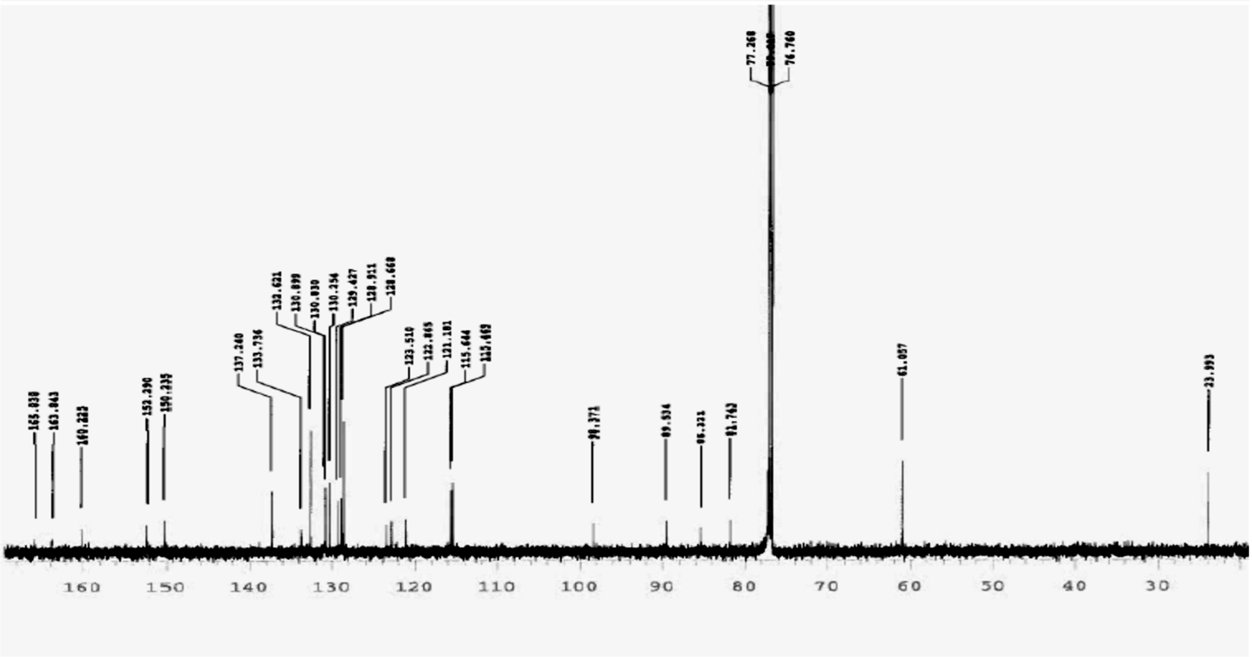

^{1}H-NMR and ^{13}C-NMR Spectra of **6b** ($CDCl_3$).

Figure S4. *Cont.*

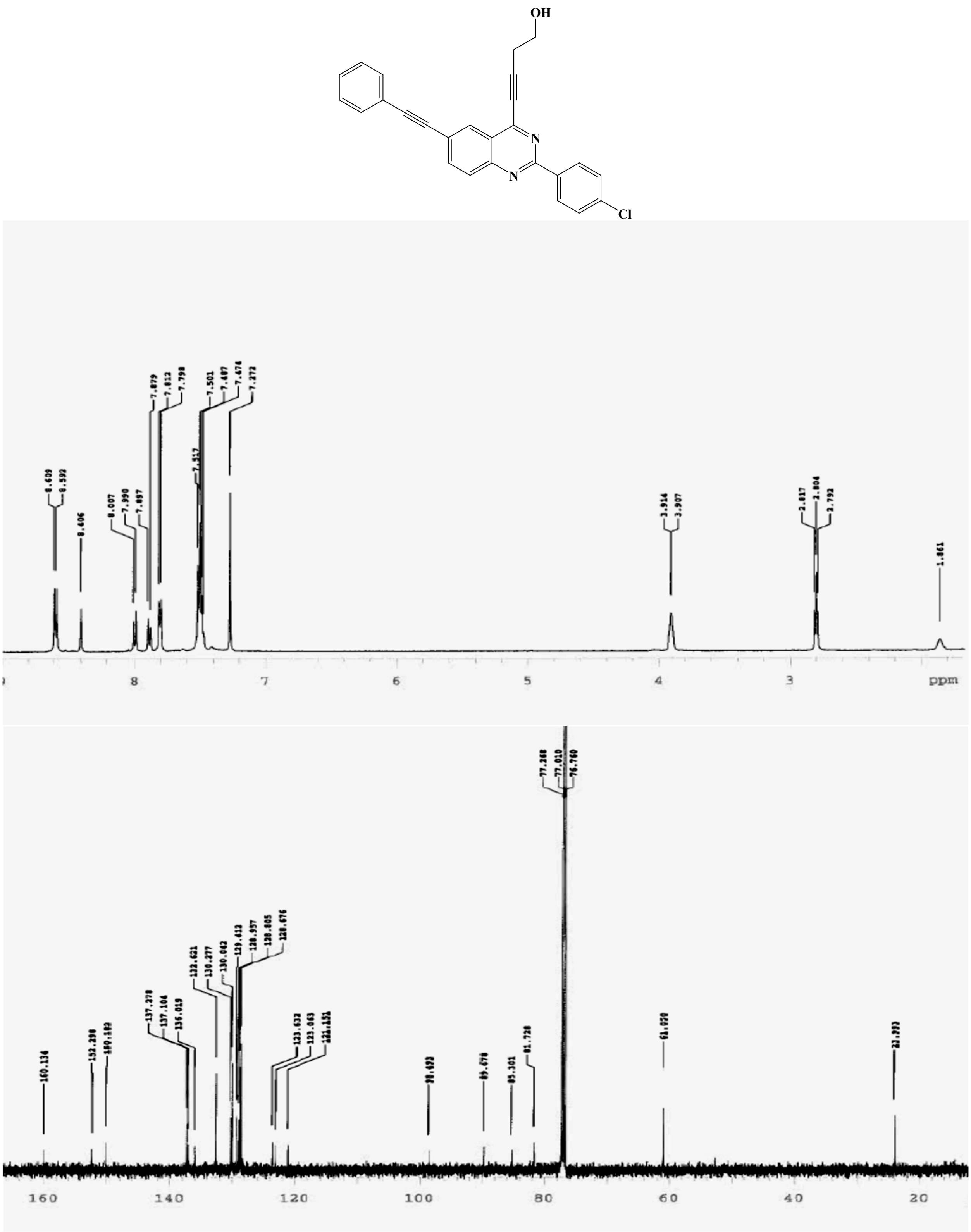

^{1}H-NMR and ^{13}C-NMR Spectra of **6c** ($CDCl_3$).

Figure S4. *Cont.*

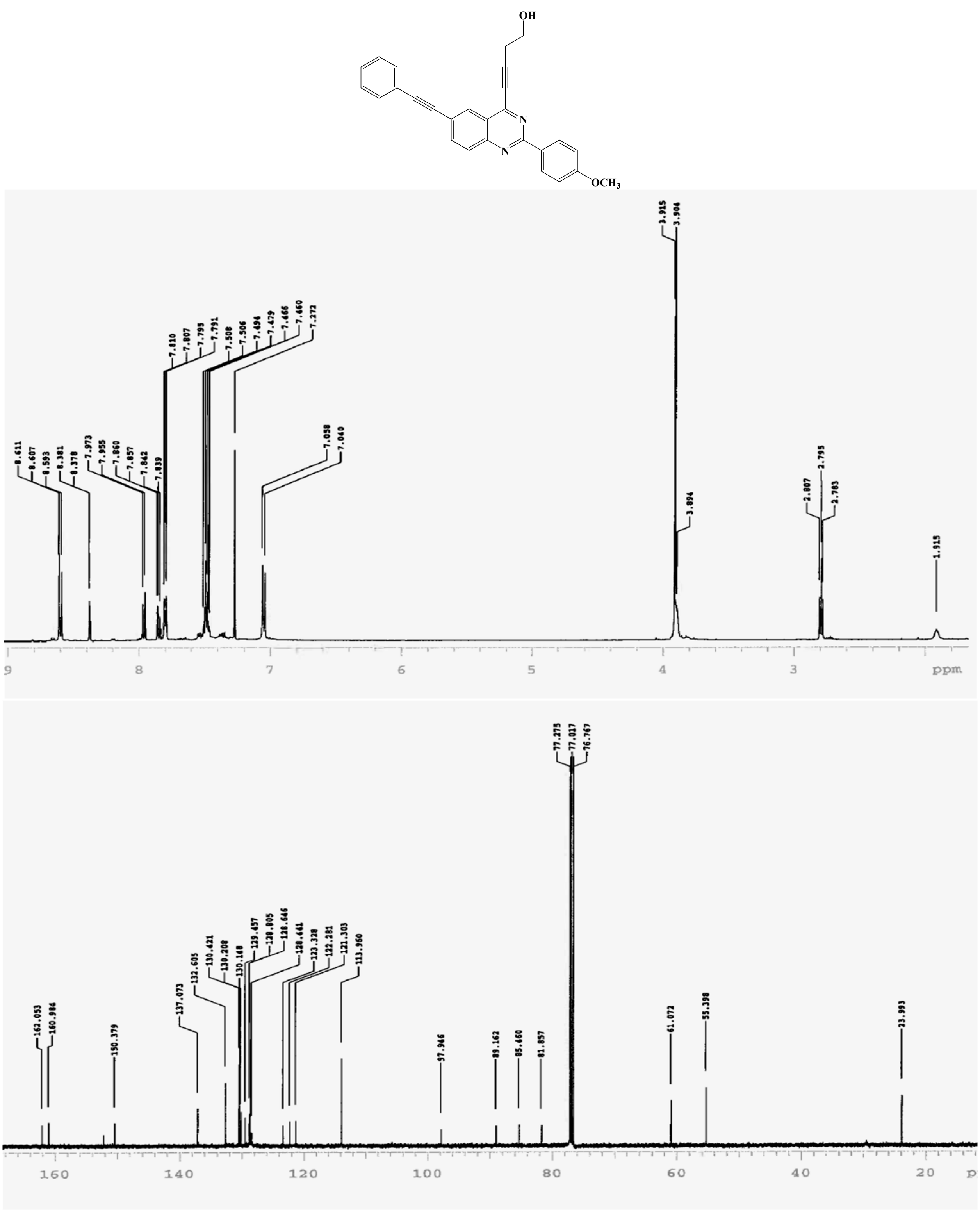

^{1}H-NMR and ^{13}C-NMR Spectra of **6d** ($CDCl_3$).

Figure S4. *Cont.*

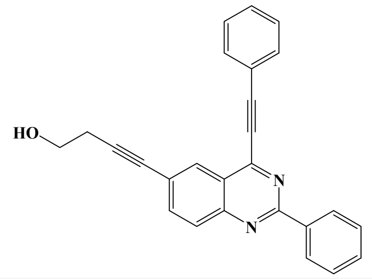

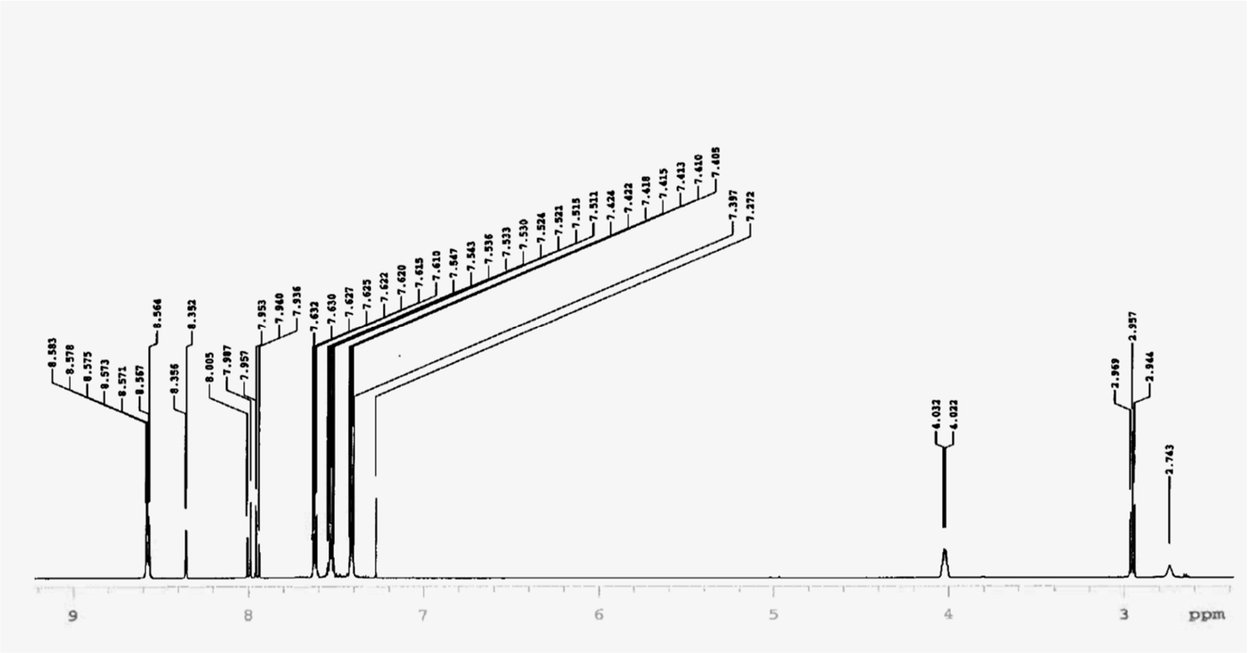

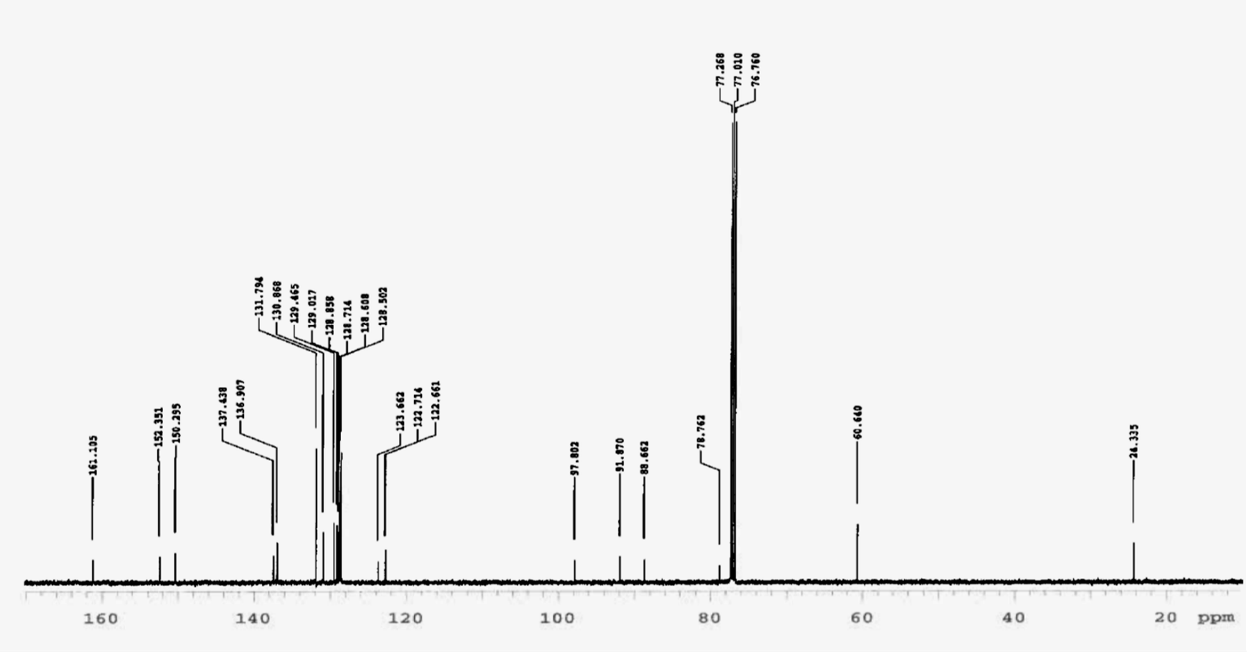

^{1}H-NMR and ^{13}C-NMR Spectra of **6e** ($CDCl_3$).

Figure S4. *Cont.*

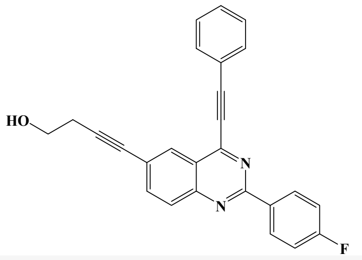

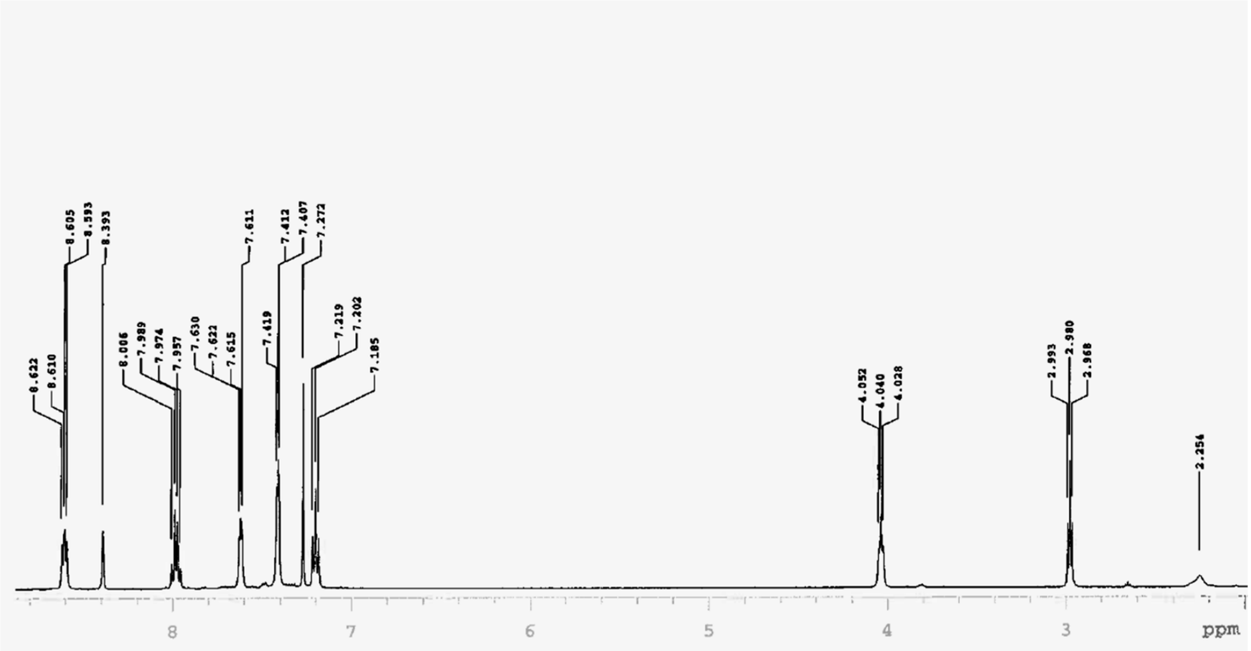

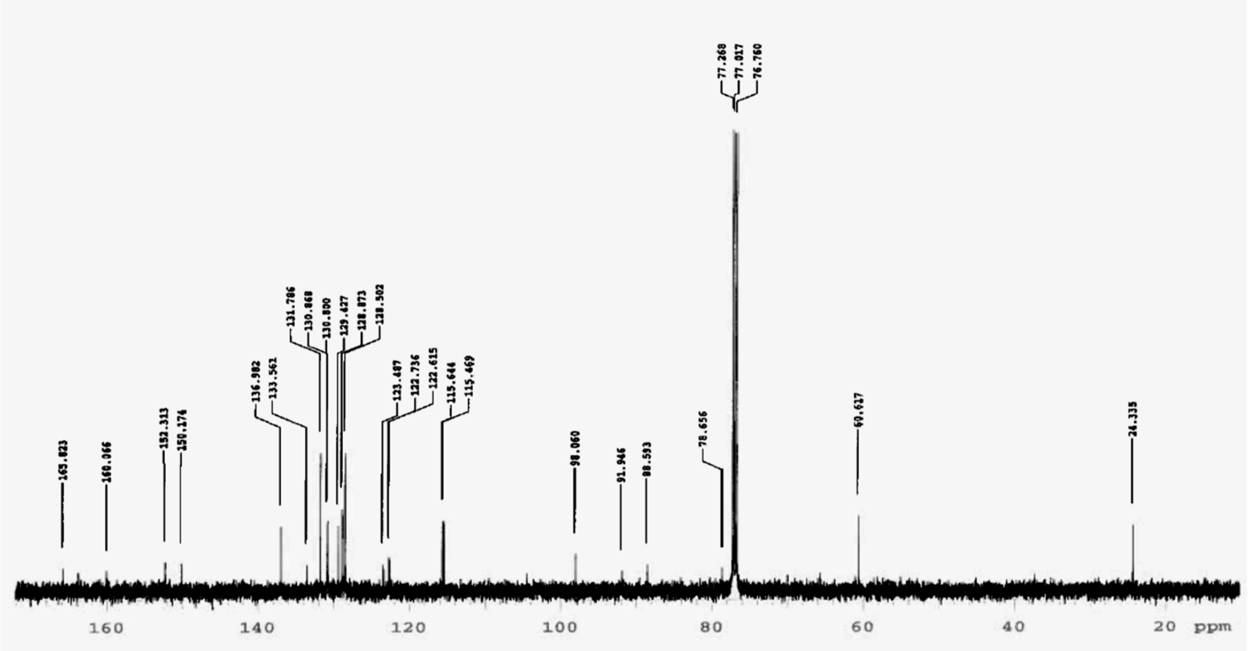

^{1}H-NMR and ^{13}C-NMR Spectra of **6f** ($CDCl_3$).

Figure S4. *Cont.*

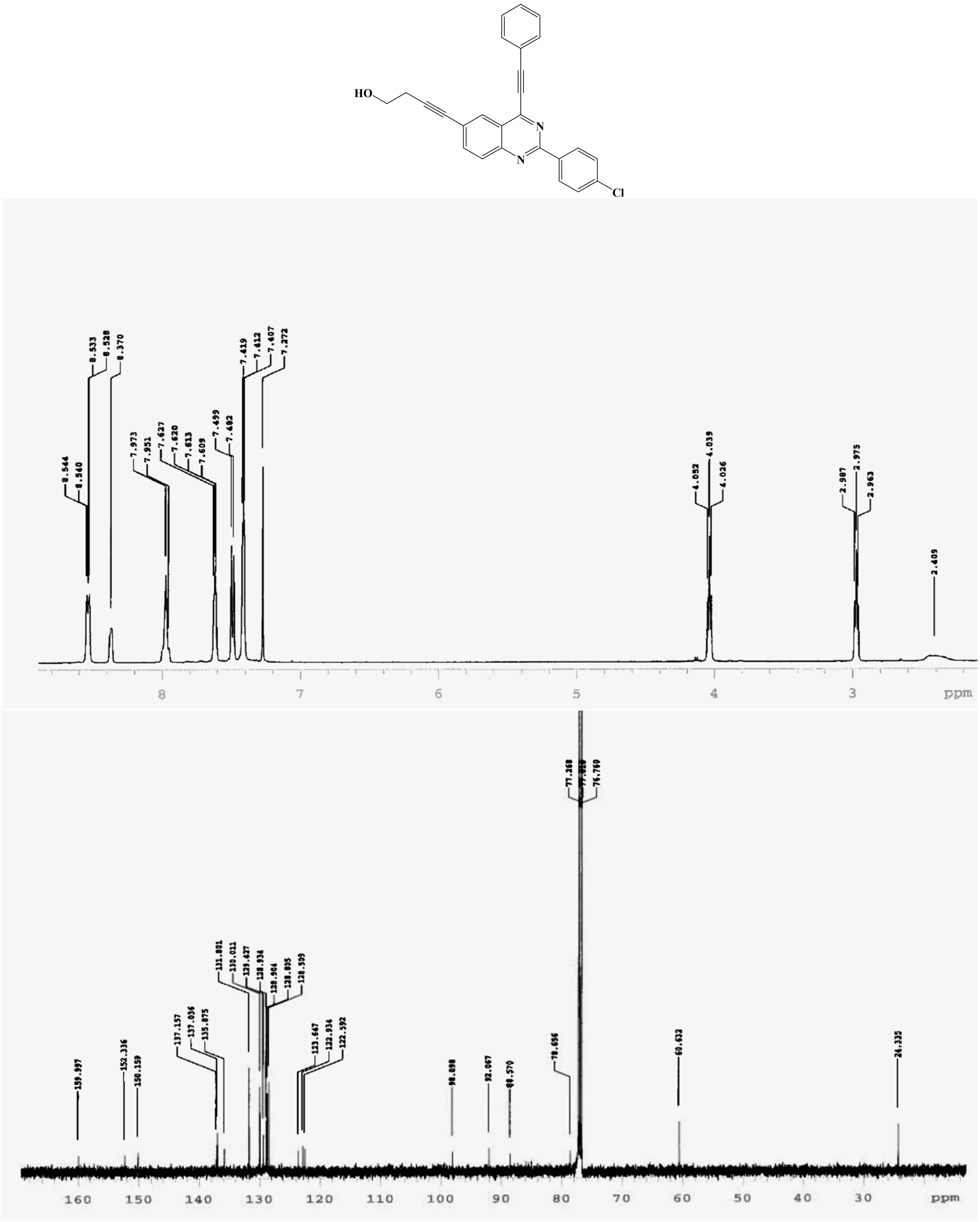

^{1}H-NMR and ^{13}C-NMR Spectra of **6g** ($CDCl_3$).

Figure S4. *Cont.*

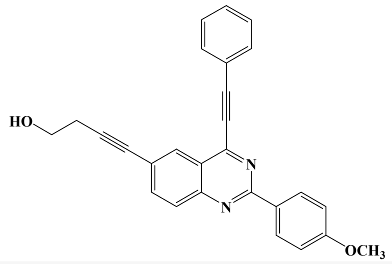

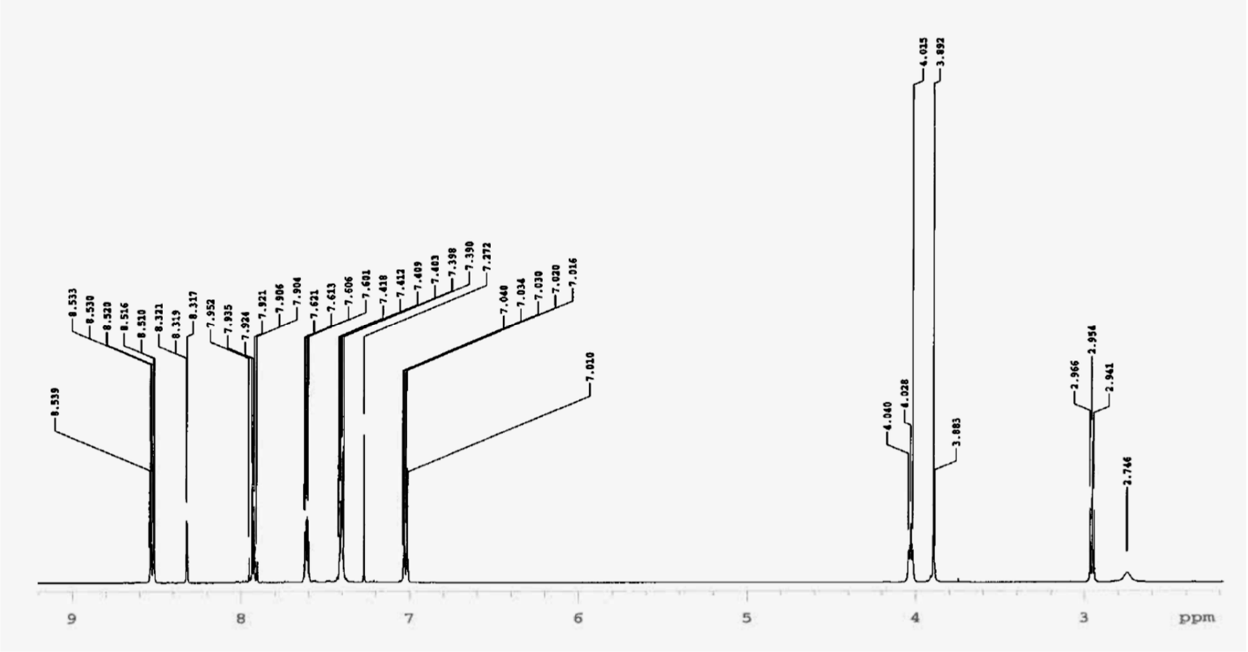

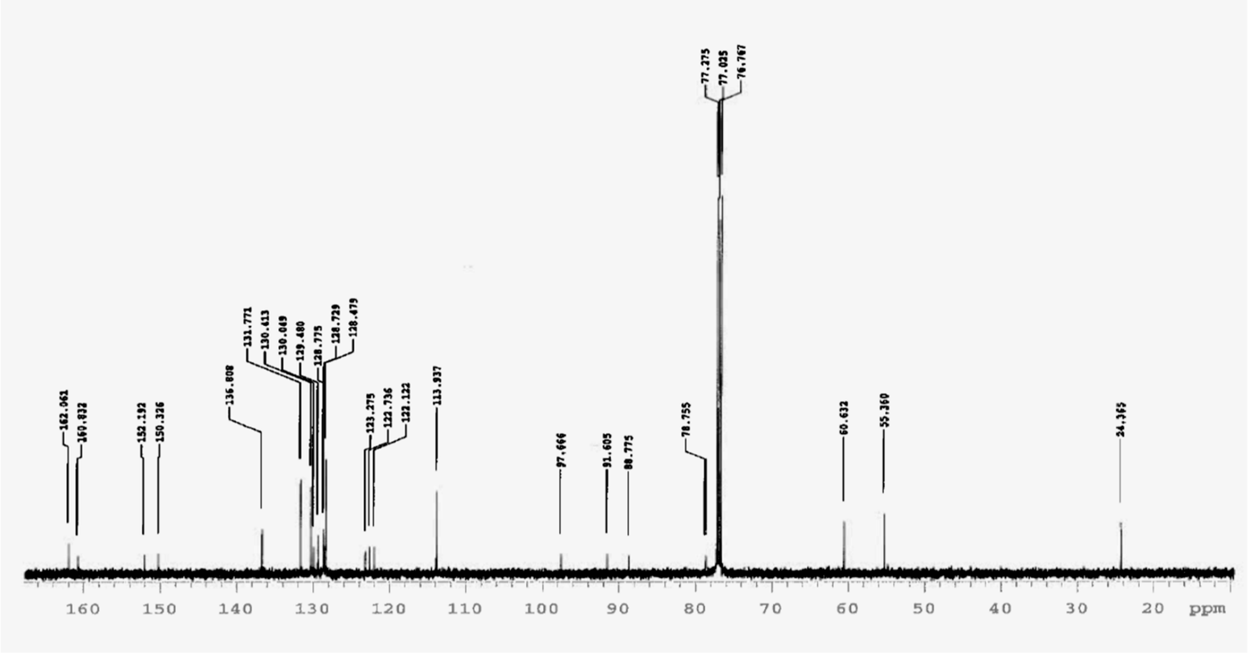

^{1}H-NMR and ^{13}C-NMR Spectra of **6h** ($CDCl_3$).

Figure S4. *Cont.*

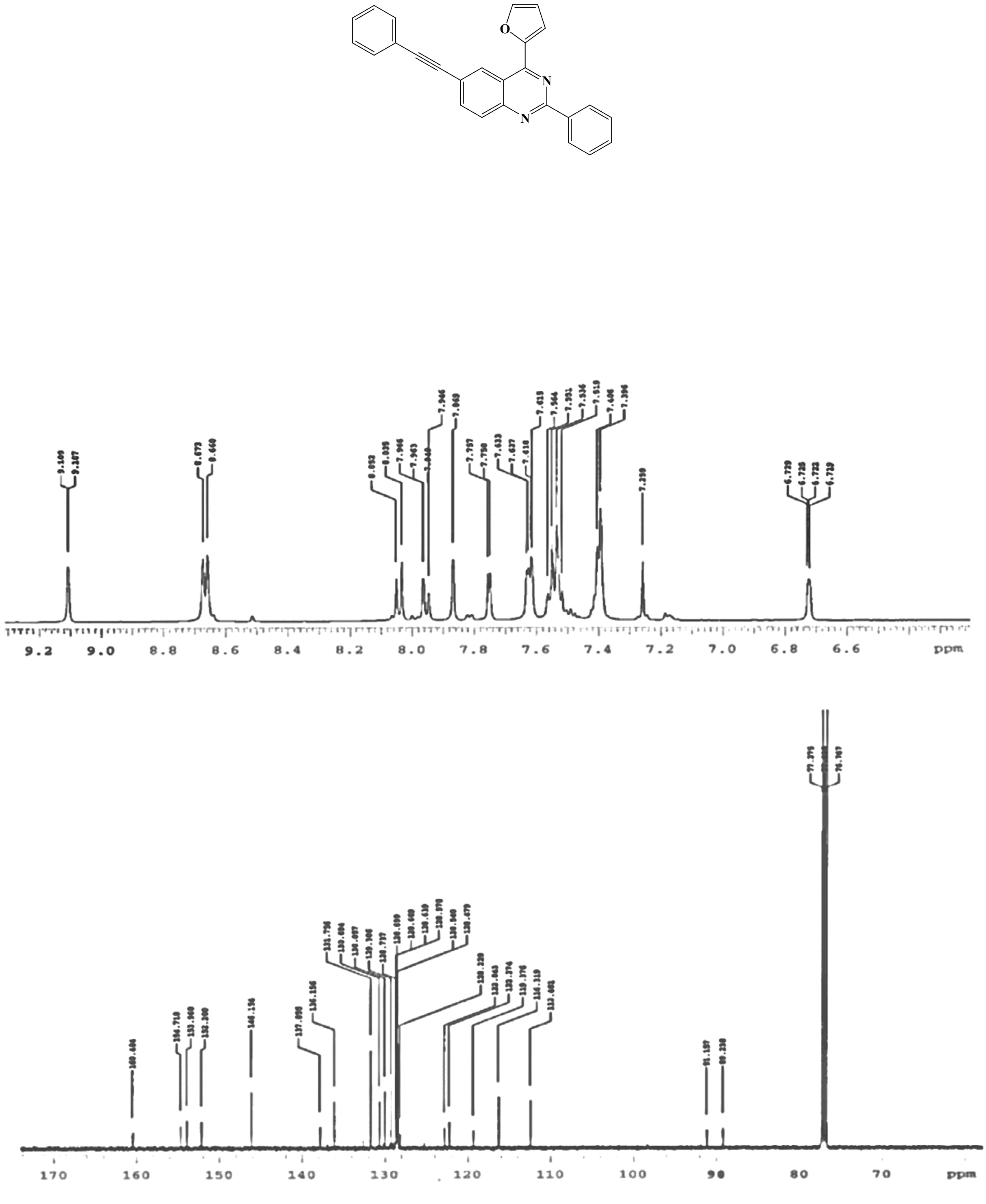

^{1}H-NMR and ^{13}C-NMR Spectra of **7a** ($CDCl_3$).

Figure S4. *Cont.*

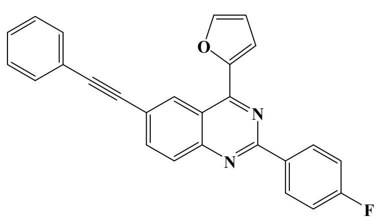

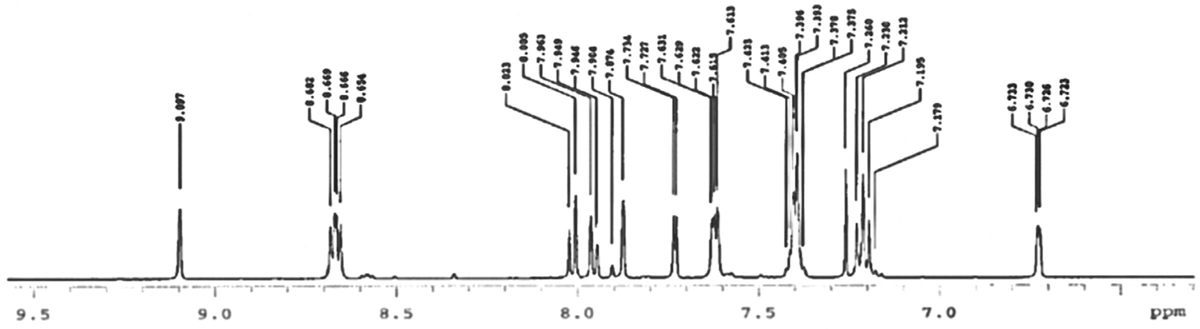

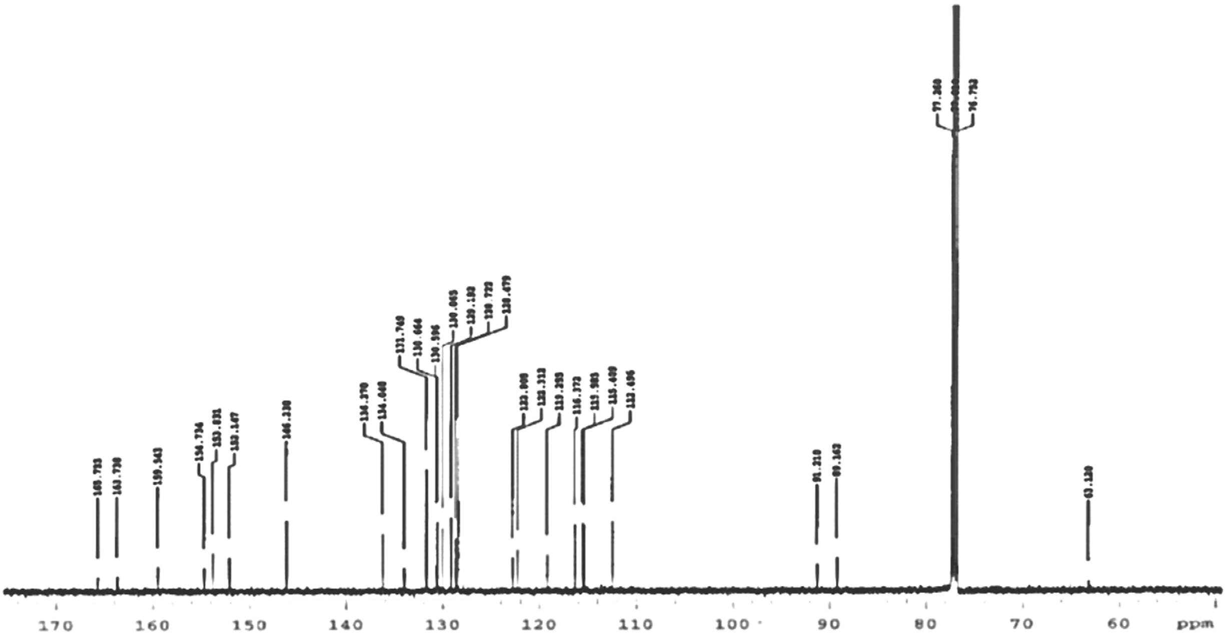

^{1}H-NMR and ^{13}C-NMR Spectra of **7b** ($CDCl_3$).

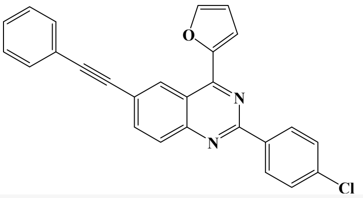

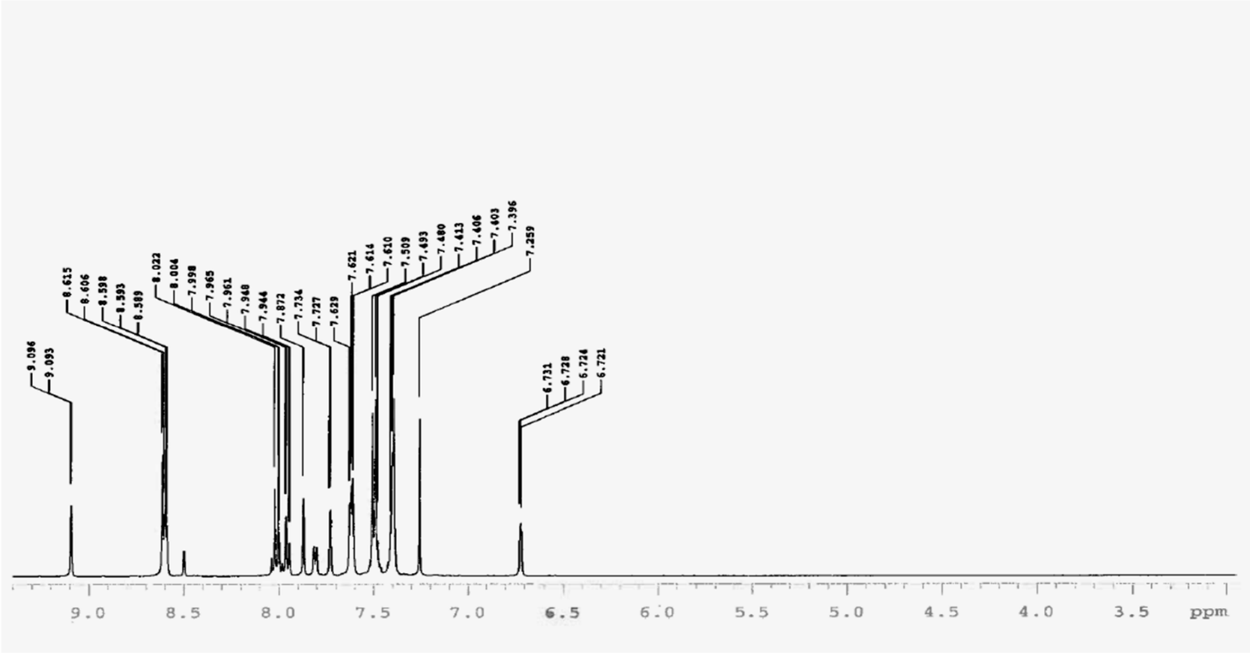

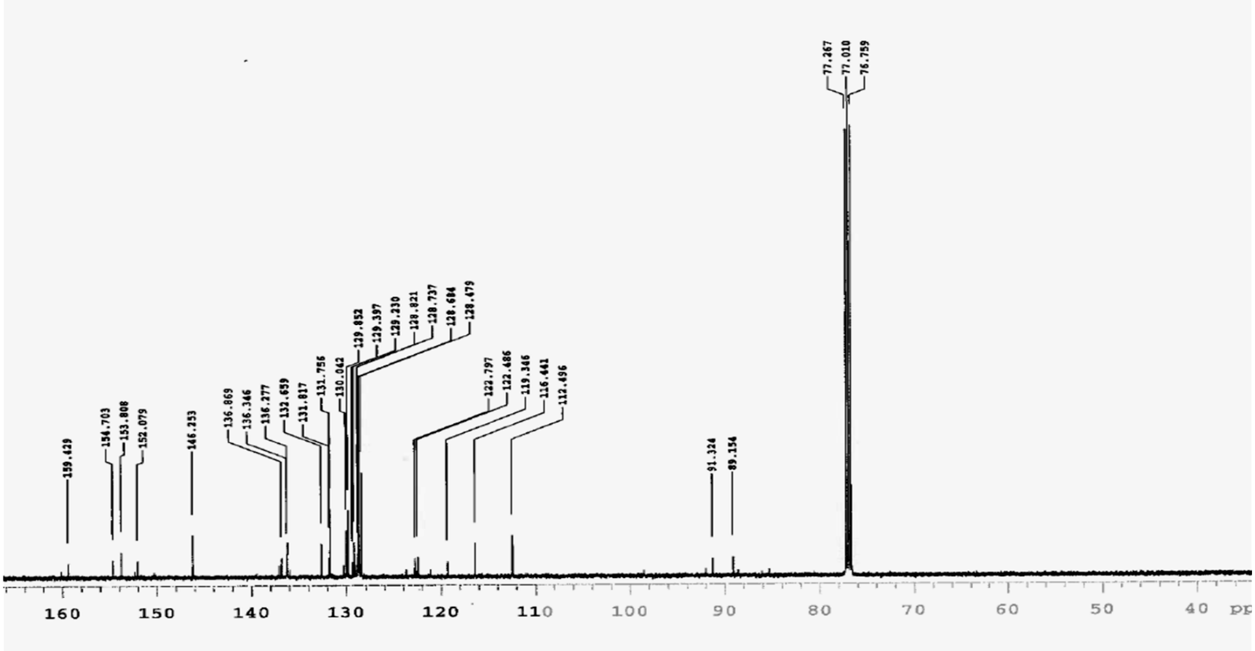

^{1}H-NMR and ^{13}C-NMR Spectra of **7c** ($CDCl_3$).

Figure S4. *Cont.*

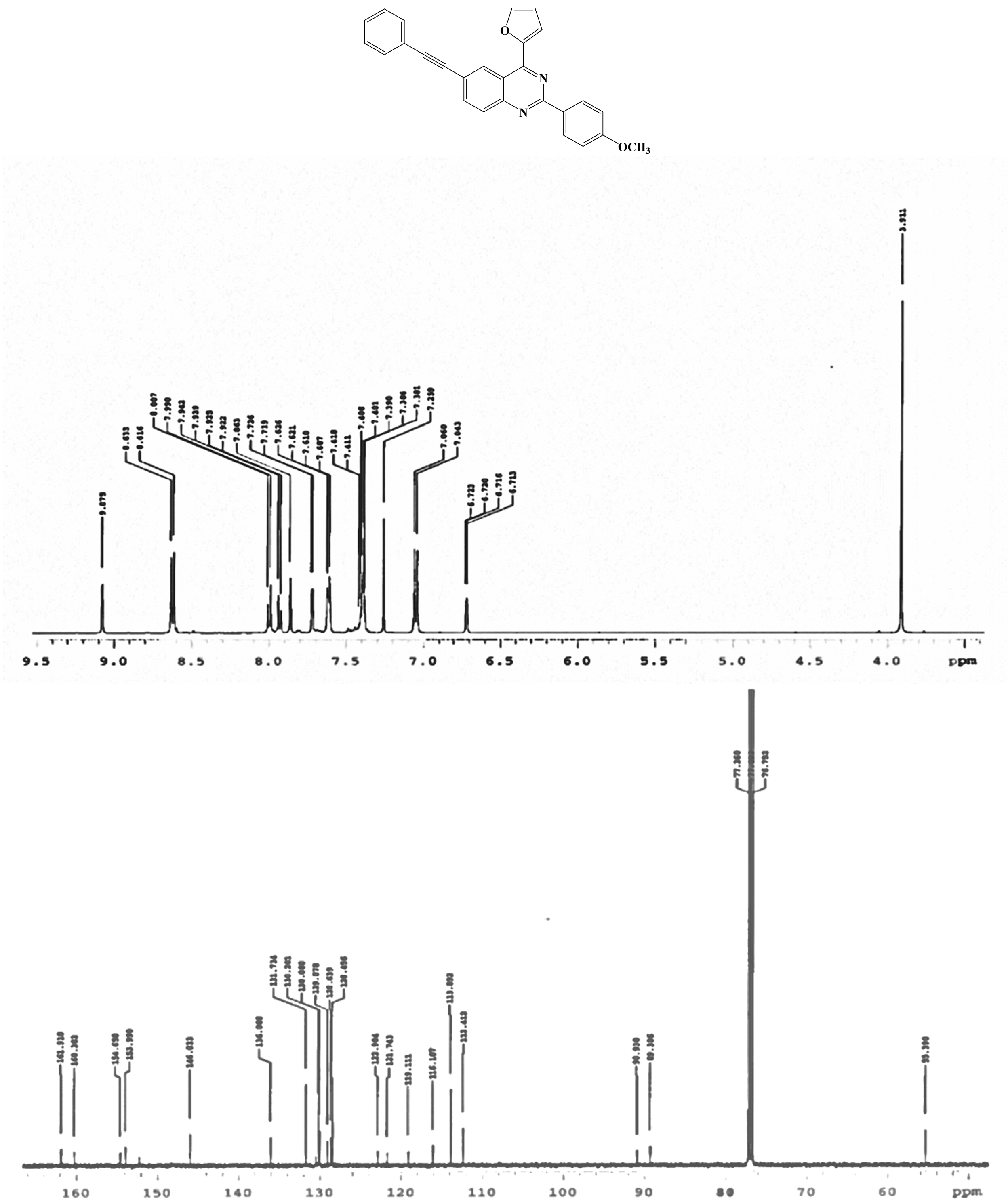

^{1}H-NMR and ^{13}C-NMR Spectra of **7d** ($CDCl_3$).

Figure S4. ^{1}H-NMR and ^{13}C-NMR Spectra of new compounds.

Table S1. Cartesian coordinates of optimized structures in the gas phase of compounds **5a**−**h**.

**5a**			
C	−1.7090130	−1.4105190	0.1102520
C	−0.8525360	−0.3299510	0.0440680
C	0.5461400	−0.5126530	0.1118310
C	1.0716860	−1.8264390	0.1940300
C	0.1789240	−2.9238820	0.2802880
C	−1.1698210	−2.7218260	0.2457900
H	−1.2638970	0.6626960	−0.0826520
C	1.5208000	0.5354230	0.0622110
H	0.6076620	−3.9161460	0.3579620
H	−1.8546160	−3.5604860	0.3037650
C	3.2037510	−1.0370400	0.0421320
N	2.8034270	0.2623160	−0.0019700
N	2.4036060	−2.0706180	0.1714870
C	1.1620860	1.9767110	0.0699070
C	1.8460580	2.8490450	−0.7814830
C	0.2110930	2.4993210	0.9500420
C	1.5679080	4.2065480	−0.7829050
H	2.6031730	2.4452600	−1.4433510
C	−0.0688760	3.8591870	0.9696360
H	−0.2964290	1.8448530	1.6496030
C	0.6103510	4.6883480	0.0947950
H	2.0802420	4.8942970	−1.4452410
H	−0.7938940	4.2821360	1.6547500
C	−3.1224800	−1.2292270	0.0350950
C	−4.3211570	−1.0871310	−0.0276240
C	−5.7375520	−0.9156570	−0.1036830
C	−6.5928960	−2.0184820	0.0164230
C	−6.2885260	0.3571740	−0.3000620
C	−7.9671920	−1.8481940	−0.0585970
H	−6.1673930	−3.0041640	0.1682910
C	−7.6638990	0.5192060	−0.3742500
H	−5.6272380	1.2113100	−0.3940170
C	−8.5065050	−0.5809610	−0.2538820
H	−8.6211000	−2.7088780	0.0356450
H	−8.0807170	1.5092400	−0.5270680
H	−9.5820930	−0.4509550	−0.3123360
F	0.3388580	6.0015640	0.1020220
C	4.6637120	−1.2836370	−0.0433970
C	5.1536490	−2.5918450	−0.0490600
C	5.5613800	−0.2171780	−0.1208500
C	6.5177470	−2.8274560	−0.1323500
H	4.4474150	−3.4109000	0.0127260
C	6.9262730	−0.4567540	−0.2040330
H	5.1728600	0.7936530	−0.1111190
C	7.4081420	−1.7608870	−0.2104850
H	6.8891830	−3.8471400	−0.1368650
H	7.6168450	0.3783930	−0.2624110
H	8.4755640	−1.9466040	−0.2755340

Table S1. *Cont.*

5b			
C	−1.9775040	−1.4181700	0.1172610
C	−1.1635150	−0.3051880	0.0516420
C	0.2409240	−0.4329290	0.1246210
C	0.8170850	−1.7250730	0.2120900
C	−0.0323780	−2.8562340	0.2977410
C	−1.3879180	−2.7070110	0.2578020
H	−1.6129610	0.6703170	−0.0788050
C	1.1732910	0.6529210	0.0751270
H	0.4341280	−3.8309660	0.3795950
H	−2.0395450	−3.5717120	0.3153820
C	2.9162220	−0.8519590	0.0666360
N	2.4661640	0.4306510	0.0166230
N	2.1578760	−1.9165490	0.1954740
C	0.7575040	2.0786280	0.0764800
C	1.4062400	2.9742930	−0.7781800
C	−0.2142420	2.5661360	0.9541960
C	1.0739410	4.3195730	−0.7850590
H	2.1787550	2.5989340	−1.4390090
C	−0.5483080	3.9137080	0.9684200
H	−0.6957120	1.8946110	1.6560120
C	0.0975660	4.7661070	0.0905460
H	1.5584770	5.0245180	−1.4502140
H	−1.2899950	4.3099900	1.6516160
C	−3.3965510	−1.2917810	0.0363490
C	−4.5994080	−1.1946120	−0.0316510
C	−6.0207590	−1.0749080	−0.1139070
C	−6.8358120	−2.2089550	−0.0052310
C	−6.6167220	0.1783250	−0.3049450
C	−8.2150400	−2.0883180	−0.0861270
H	−6.3754310	−3.1794840	0.1424530
C	−7.9966900	0.2906500	−0.3849880
H	−5.9865460	1.0565630	−0.3901200
C	−8.7991730	−0.8402760	−0.2759160
H	−8.8375990	−2.9728310	−0.0007630
H	−8.4486230	1.2658030	−0.5335780
H	−9.8784860	−0.7491190	−0.3389510
F	−0.2262890	6.0671720	0.0926690
C	4.3834640	−1.0400390	−0.0113110
C	4.9269490	−2.3277410	−0.0093560
C	5.2394610	0.0609870	−0.0890970
C	6.2973490	−2.5174470	−0.0851580
H	4.2545030	−3.1745180	0.0527220
C	6.6131120	−0.1148720	−0.1654960
H	4.8114020	1.0555610	−0.0853340
C	7.1182210	−1.4038410	−0.1625710
H	6.7384620	−3.5072700	−0.0859270
H	7.2943550	0.7257920	−0.2248360
F	8.4465870	−1.5812550	−0.2364960

Table S1. *Cont.*

5c			
C	−2.2691240	−1.4282850	0.1217220
C	−1.4888970	−0.2913540	0.0562870
C	−0.0815110	−0.3764790	0.1343750
C	0.5325690	−1.6507130	0.2269900
C	−0.2823510	−2.8068590	0.3120350
C	−1.6416530	−2.6985680	0.2670900
H	−1.9671630	0.6697570	−0.0782320
C	0.8172920	0.7372990	0.0853040
H	0.2130200	−3.7668980	0.3976780
H	−2.2671760	−3.5823250	0.3243810
C	2.6037890	−0.7143000	0.0873820
N	2.1167600	0.5537090	0.0320790
N	1.8788910	−1.8013580	0.2155950
C	0.3587420	2.1496030	0.0811180
C	0.9837490	3.0621200	−0.7734940
C	−0.6307660	2.6099220	0.9536870
C	0.6111050	4.3966880	−0.7854440
H	1.7697380	2.7086460	−1.4305020
C	−1.0051850	3.9468490	0.9628570
H	−1.0946020	1.9262770	1.6556740
C	−0.3817580	4.8159840	0.0851340
H	1.0767750	5.1141330	−1.4506960
H	−1.7610840	4.3224150	1.6421490
C	−3.6909410	−1.3443170	0.0357410
C	−4.8958260	−1.2816990	−0.0364530
C	−6.3196850	−1.2017810	−0.1235520
C	−6.9500430	0.0360450	−0.3042150
C	−7.1025650	−2.3595600	−0.0298680
C	−8.3323270	0.1101370	−0.3887110
H	−6.3446940	0.9326030	−0.3777920
C	−8.4843250	−2.2771070	−0.1152190
H	−6.6154680	−3.3181610	0.1097630
C	−9.1027910	−1.0441390	−0.2945300
H	−8.8111390	1.0735910	−0.5291150
H	−9.0819450	−3.1796720	−0.0415270
H	−10.1840000	−0.9829210	−0.3610360
F	−0.7446300	6.1065070	0.0822940
C	4.0776490	−0.8587500	0.0164110
C	4.6601150	−2.1278500	0.0252230
C	4.9002280	0.2658090	−0.0614220
C	6.0362330	−2.2752610	−0.0440310
H	4.0156590	−2.9961880	0.0872660
C	6.2789790	0.1305140	−0.1311740
H	4.4446710	1.2481400	−0.0630980
C	6.8355980	−1.1412960	−0.1223290
H	6.4907340	−3.2588150	−0.0382940
H	6.9200660	1.0019770	−0.1902440
Cl	8.5712310	−1.3196170	−0.2103650

Table S1. *Cont.*

5d			
C	−2.2417810	−1.4332100	0.1249080
C	−1.4854530	−0.2803500	0.0575670
C	−0.0767320	−0.3351150	0.1363240
C	0.5657670	−1.5951350	0.2312450
C	−0.2257780	−2.7680500	0.3185160
C	−1.5870080	−2.6892870	0.2728870
H	−1.9839600	0.6702070	−0.0790720
C	0.7992320	0.7967250	0.0853490
H	0.2900550	−3.7171030	0.4063010
H	−2.1933040	−3.5863300	0.3318580
C	2.6195530	−0.6162880	0.0891550
N	2.1013890	0.6418240	0.0324520
N	1.9138690	−1.7180060	0.2205940
C	0.3105260	2.1996310	0.0786290
C	0.9176370	3.1238240	−0.7762250
C	−0.6898380	2.6408490	0.9484750
C	0.5171390	4.4503740	−0.7912100
H	1.7128720	2.7854970	−1.4300610
C	−1.0925950	3.9697200	0.9545170
H	−1.1405200	1.9487450	1.6507430
C	−0.4860530	4.8502330	0.0765230
H	0.9692740	5.1763530	−1.4565890
H	−1.8576610	4.3303340	1.6316870
C	−3.6652090	−1.3793620	0.0383950
C	−4.8711940	−1.3422610	−0.0340350
C	−6.2963870	−1.2924890	−0.1214950
C	−6.9537760	−0.0675280	−0.2937740
C	−7.0545390	−2.4673380	−0.0365570
C	−8.3373360	−0.0227120	−0.3785560
H	−6.3679480	0.8424370	−0.3605990
C	−8.4377570	−2.4142390	−0.1221890
H	−6.5469020	−3.4161680	0.0965730
C	−9.0829470	−1.1938520	−0.2931040
H	−8.8366610	0.9312660	−0.5123670
H	−9.0156190	−3.3301540	−0.0552580
H	−10.1652160	−1.1555660	−0.3598290
F	−0.8762580	6.1333910	0.0706430
C	4.0910570	−0.7265510	0.0156300
C	4.7070480	−1.9765260	0.0276440
C	4.8963440	0.4159460	−0.0694370
C	6.0879110	−2.0996410	−0.0442930
H	4.0839500	−2.8603780	0.0945950
C	6.2704400	0.3072470	−0.1417570
H	4.4217050	1.3893780	−0.0740120
C	6.8767420	−0.9521140	−0.1301420
H	6.5352770	−3.0852630	−0.0335990
H	6.9049890	1.1838310	−0.2063240
O	8.2312210	−0.9556270	−0.2056090
C	8.8987060	−2.1994510	−0.1943220
H	9.9626950	−1.9737430	−0.2617610
H	8.7062950	−2.7524380	0.7322670
H	8.6081200	−2.8214330	−1.0488040

Table S1. *Cont.*

5e			
C	1.9982260	−1.6717670	0.1102090
C	1.1773060	−0.5624050	0.0689140
C	−0.2259440	−0.6989200	0.1445400
C	−0.7938610	−1.9960340	0.2051790
C	0.0622180	−3.1236320	0.2671640
C	1.4169760	−2.9662530	0.2274840
H	1.6202330	0.4180740	−0.0447220
C	−1.1636010	0.3853030	0.1209180
H	−0.3987780	−4.1024250	0.3299900
H	2.0735680	−3.8282790	0.2666350
C	−2.8964250	−1.1323750	0.0729360
N	−2.4557460	0.1532600	0.0541000
N	−2.1337040	−2.1960620	0.1826100
C	−0.7554080	1.8094400	0.1576580
C	−1.4227840	2.7302840	−0.6476590
C	0.2361790	2.2850380	1.0256590
C	−1.0986010	4.0801970	−0.6278620
H	−2.2101170	2.3745370	−1.3023810
C	0.5590860	3.6278560	1.0671420
H	0.7383270	1.6010600	1.7007840
C	−0.0992900	4.5344290	0.2327130
H	−1.6286010	4.7637710	−1.2785420
H	1.3146040	4.0058220	1.7461960
C	3.4163430	−1.5350170	0.0267870
C	4.6183470	−1.4288650	−0.0431060
C	6.0385740	−1.2980980	−0.1275940
C	6.6266350	−0.0358350	−0.2800130
C	6.8609770	−2.4300550	−0.0597740
C	8.0055770	0.0872550	−0.3625040
H	5.9909150	0.8409370	−0.3331310
C	8.2391070	−2.2986810	−0.1428530
H	6.4069130	−3.4076140	0.0581310
C	8.8152710	−1.0417210	−0.2942790
H	8.4510470	1.0695360	−0.4808230
H	8.8671170	−3.1818770	−0.0892400
H	9.8937560	−0.9421430	−0.3590500
C	−4.3627570	−1.3282010	−0.0158680
C	−4.8980120	−2.6188230	−0.0484470
C	−5.2255720	−0.2312570	−0.0701720
C	−6.2669180	−2.8154290	−0.1354160
H	−4.2199800	−3.4622760	−0.0041790
C	−6.5978290	−0.4138750	−0.1574040
H	−4.8030100	0.7652160	−0.0393900
C	−7.0946180	−1.7056030	−0.1890180
H	−6.7015920	−3.8077500	−0.1628180
H	−7.2843330	0.4236220	−0.1988610
F	−8.4219570	−1.8895850	−0.2737050
O	0.2963500	5.8263120	0.3370020
C	−0.3450820	6.7911660	−0.4712420
H	0.1191470	7.7465250	−0.2285770
H	−0.2027260	6.5824020	−1.5376750
H	−1.4182530	6.8475160	−0.2565310

Table S1. *Cont.*

5f			
C	2.2553140	−1.6940530	−0.1144020
C	1.4784810	−0.5533570	−0.0741280
C	0.0712770	−0.6343770	−0.1560010
C	−0.5463990	−1.9083210	−0.2220490
C	0.2646670	−3.0686320	−0.2825200
C	1.6243940	−2.9646060	−0.2367220
H	1.9591560	0.4086270	0.0436550
C	−0.8225490	0.4862170	−0.1335530
H	−0.2341250	−4.0284250	−0.3494080
H	2.2468340	−3.8516170	−0.2749000
C	−2.6128160	−0.9623480	−0.0974790
N	−2.1234110	0.3045740	−0.0730530
N	−1.8934380	−2.0553380	−0.2057930
C	−0.3590380	1.8931970	−0.1641070
C	−0.9955620	2.8370830	0.6396510
C	0.6556770	2.3323770	−1.0246510
C	−0.6195100	4.1734490	0.6256590
H	−1.8000050	2.5104780	1.2887340
C	1.0302520	3.6618180	−1.0603330
H	1.1352940	1.6314720	−1.6987920
C	0.4020170	4.5910370	−0.2274540
H	−1.1268820	4.8752820	1.2749200
H	1.8039310	4.0121200	−1.7337350
C	3.6772510	−1.6126720	−0.0250890
C	4.8821100	−1.5520840	0.0494730
C	6.3059150	−1.4742430	0.1389940
C	6.9406800	−0.2329260	0.2751780
C	7.0849440	−2.6374710	0.0919940
C	8.3229520	−0.1608930	0.3620060
H	6.3384760	0.6680010	0.3122080
C	8.4667210	−2.5570970	0.1794060
H	6.5946310	−3.5988230	−0.0132950
C	9.0894640	−1.3206490	0.3144250
H	8.8049170	0.8054760	0.4675340
H	9.0610160	−3.4641190	0.1419950
H	10.1706790	−1.2609820	0.3825510
C	−4.0874030	−1.1011110	−0.0166880
C	−4.6735490	−2.3681710	0.0096530
C	−4.9065680	0.0272740	0.0360530
C	−6.0498360	−2.5099150	0.0891980
H	−4.0314310	−3.2394090	−0.0332730
C	−6.2854320	−0.1021000	0.1157250
H	−4.4473200	1.0075620	0.0101700
C	−6.8455430	−1.3720510	0.1422360
H	−6.5070890	−3.4920000	0.1108400
H	−6.9239210	0.7724090	0.1552480
O	0.8475630	5.8667670	−0.3256030
C	0.2374190	6.8538770	0.4802900
H	0.7396560	7.7912310	0.2432880
H	−0.8311440	6.9514990	0.2576740
H	0.3637760	6.6374410	1.5471760
Cl	−8.5818240	−1.5429730	0.2432680

Table S1. *Cont.*

5g			
C	2.2187300	−1.7048410	0.1168700
C	1.4685600	−0.5464600	0.0750700
C	0.0600350	−0.5939120	0.1579180
C	−0.5889450	−1.8521270	0.2264190
C	0.1958890	−3.0310290	0.2886090
C	1.5576000	−2.9596670	0.2416840
H	1.9716760	0.4038160	−0.0447460
C	−0.8082210	0.5466180	0.1339670
H	−0.3255250	−3.9786170	0.3576610
H	2.1586710	−3.8613330	0.2811940
C	−2.6355080	−0.8589950	0.1004840
N	−2.1120640	0.3967760	0.0744430
N	−1.9379750	−1.9681600	0.2115950
C	−0.3113660	1.9431830	0.1618640
C	−0.9265530	2.9001070	−0.6428590
C	0.7139370	2.3605590	1.0203770
C	−0.5199030	4.2276700	−0.6315360
H	−1.7397370	2.5906460	−1.2894270
C	1.1194360	3.6812420	1.0531850
H	1.1781560	1.6497930	1.6949970
C	0.5118440	4.6230640	0.2195790
H	−1.0121460	4.9399070	−1.2811710
H	1.9019540	4.0146360	1.7250130
C	3.6422910	−1.6570300	0.0269180
C	4.8483070	−1.6251800	−0.0478430
C	6.2735140	−1.5813200	−0.1373330
C	6.9389080	−0.3549380	−0.2628570
C	7.0243440	−2.7633970	−0.1007760
C	8.3225640	−0.3159220	−0.3493740
H	6.3589440	0.5607630	−0.2917960
C	8.4076990	−2.7161050	−0.1878550
H	6.5107060	−3.7133860	−0.0037800
C	9.0607530	−1.4942680	−0.3121630
H	8.8278510	0.6393920	−0.4465140
H	8.9795230	−3.6378040	−0.1585460
H	10.1431190	−1.4604590	−0.3800180
C	−4.1080470	−0.9603020	0.0181780
C	−4.7304170	−2.2068260	−0.0017400
C	−4.9072340	0.1877520	−0.0437000
C	−6.1117330	−2.3212280	−0.0826490
H	−4.1117570	−3.0949670	0.0473770
C	−6.2817010	0.0878490	−0.1247470
H	−4.4268260	1.1581560	−0.0230250
C	−6.8943260	−1.1681600	−0.1452200
H	−6.5639910	−3.3046070	−0.0969910
H	−6.9116140	0.9689560	−0.1714270
O	0.9867840	5.8892650	0.3150460
C	0.3971020	6.8877830	−0.4912790
H	0.9204380	7.8144340	−0.2574800
H	0.5159150	6.6667050	−1.5581630
H	−0.6686720	7.0096970	−0.2668600
O	−8.2491270	−1.1630980	−0.2267890
C	−8.9219620	−2.4036380	−0.2446630
H	−8.7348240	−2.9778050	0.6700980
H	−9.9848570	−2.1720340	−0.3103450
H	−8.6315650	−3.0084310	−1.1115450

Table S1. *Cont.*

5h			
C	−2.5501110	−1.4254260	−0.2404200
C	−1.6101510	−0.4167120	−0.1949620
C	−0.2375550	−0.7130140	−0.0388180
C	0.1767970	−2.0670560	0.0143870
C	−0.8018750	−3.0912630	−0.0147050
C	−2.1254950	−2.7797990	−0.1320470
H	−1.9329050	0.6101950	−0.3031250
C	0.8150310	0.2567960	0.0309230
H	−0.4577390	−4.1173600	0.0429560
H	−2.8769640	−3.5608870	−0.1627090
C	2.3678710	−1.4455020	0.0445110
N	2.0748830	−0.1181170	0.0390170
N	1.4849160	−2.4172060	0.0698740
C	0.5688310	1.7178360	0.0868540
C	1.3904780	2.5744580	−0.6430900
C	−0.4183460	2.2841310	0.9035990
C	1.2231580	3.9522080	−0.5998630
H	2.1748580	2.1452880	−1.2560060
C	−0.5868760	3.6542130	0.9683100
H	−1.0407710	1.6464950	1.5214100
C	0.2270520	4.4982430	0.2092820
H	1.8711240	4.5852790	−1.1922300
H	−1.3384910	4.1007420	1.6090310
C	−3.9404320	−1.1302120	−0.4041190
C	−5.1171200	−0.9072360	−0.5435430
C	3.8077500	−1.8054880	0.0450800
C	4.1944360	−3.1472470	0.0084100
C	4.7886410	−0.8128950	0.0810240
C	5.5388130	−3.4881790	0.0064640
H	3.4240410	−3.9083690	−0.0179270
C	6.1335430	−1.1575690	0.0788060
H	4.4786160	0.2242300	0.1144720
C	6.5125720	−2.4946910	0.0412700
H	5.8296210	−4.5334060	−0.0227900
H	6.8890110	−0.3788880	0.1083950
O	−0.0225220	5.8253650	0.3316100
C	0.7830560	6.7278430	−0.3972280
H	0.4202370	7.7252660	−0.1506990
H	1.8380410	6.6476600	−0.1114410
H	0.6910770	6.5685330	−1.4777050
H	7.5643690	−2.7629860	0.0398400
C	−6.5416020	−0.6226890	−0.6988960
H	−6.6955580	0.4601770	−0.7647680
H	−6.9047900	−1.0545560	−1.6380250
C	−7.3966990	−1.1724290	0.4376830
H	−7.0237860	−0.7839080	1.3952580
H	−8.4275410	−0.8112350	0.3055490
O	−7.3325990	−2.5813580	0.3857060
H	−7.7706740	−2.9369940	1.1662800

Table S2. Cartesian coordinates of optimized structures in DCM of compounds **5a**−**h**.

**5a**			
C	−1.7053770	−1.4095080	−0.1097670
C	−0.8493610	−0.3279980	−0.0462040
C	0.5489710	−0.5127180	−0.1114370
C	1.0736750	−1.8266760	−0.1917300
C	0.1816560	−2.9244070	−0.2752550
C	−1.1676760	−2.7218270	−0.2409960
H	−1.2595090	0.6659580	0.0737790
C	1.5217420	0.5368240	−0.0632150
H	0.6048830	−3.9192600	−0.3509970
H	−1.8506860	−3.5619420	−0.2970630
C	3.2066880	−1.0340730	−0.0435760
N	2.8059480	0.2648980	−0.0025540
N	2.4077840	−2.0690460	−0.1692180
C	1.1588390	1.9766190	−0.0692890
C	1.8212360	2.8466810	0.8018370
C	0.2218280	2.4978260	−0.9658250
C	1.5372490	4.2035020	0.8041710
H	2.5628980	2.4461670	1.4829480
C	−0.0633190	3.8568000	−0.9848120
H	−0.2709950	1.8457700	−1.6778290
C	0.5959650	4.6828850	−0.0920330
H	2.0326000	4.8883300	1.4821960
H	−0.7778100	4.2775280	−1.6821410
C	−3.1195900	−1.2277130	−0.0358310
C	−4.3188480	−1.0862090	0.0266110
C	−5.7364960	−0.9176830	0.1022000
C	−6.5870430	−2.0278850	0.0116840
C	−6.2910110	0.3585920	0.2685100
C	−7.9623860	−1.8608460	0.0863880
H	−6.1595210	−3.0160950	−0.1168930
C	−7.6675180	0.5166610	0.3425430
H	−5.6343540	1.2186460	0.3390200
C	−8.5059950	−0.5904040	0.2518800
H	−8.6130170	−2.7260410	0.0153600
H	−8.0879820	1.5083570	0.4716860
H	−9.5818280	−0.4632560	0.3100940
F	0.3189490	5.9974980	−0.0989990
C	4.6686770	−1.2792100	0.0415650
C	5.1645750	−2.5859550	0.0317440
C	5.5647800	−0.2118240	0.1354720
C	6.5298970	−2.8191600	0.1135540
H	4.4650220	−3.4098390	−0.0401820
C	6.9310450	−0.4478910	0.2176850
H	5.1767080	0.7990970	0.1415680
C	7.4177120	−1.7508630	0.2068390
H	6.9037990	−3.8377950	0.1052580
H	7.6182190	0.3888530	0.2895000
H	8.4854210	−1.9341820	0.2708210

Table S2. *Cont.*

**5b**			
C	−1.9739920	−1.4181330	−0.1103250
C	−1.1605850	−0.3040620	−0.0492590
C	0.2435980	−0.4339240	−0.1192380
C	0.8189320	−1.7264300	−0.2024090
C	−0.0297220	−2.8581500	−0.2822360
C	−1.3858750	−2.7084210	−0.2430340
H	−1.6090110	0.6731110	0.0719060
C	1.1741240	0.6533600	−0.0734560
H	0.4317280	−3.8357020	−0.3598300
H	−2.0357100	−3.5746000	−0.2967320
C	2.9189440	−0.8497950	−0.0658220
N	2.4684970	0.4324550	−0.0201280
N	2.1617570	−1.9159940	−0.1871610
C	0.7541740	2.0775320	−0.0732800
C	1.3811990	2.9697290	0.8016060
C	−0.2032790	2.5644920	−0.9674820
C	1.0431350	4.3140870	0.8095690
H	2.1379430	2.5966240	1.4817010
C	−0.5424780	3.9110280	−0.9808630
H	−0.6700880	1.8961720	−1.6819550
C	0.0834060	4.7591960	−0.0847350
H	1.5106230	5.0153030	1.4906180
H	−1.2734100	4.3056890	−1.6763890
C	−3.3939360	−1.2908500	−0.0328920
C	−4.5974710	−1.1937880	0.0318900
C	−6.0201010	−1.0760930	0.1099450
C	−6.6193470	0.1804140	0.2717000
C	−6.8307680	−2.2162270	0.0260860
C	−8.0004590	0.2898000	0.3476540
H	−5.9935520	1.0635690	0.3371020
C	−8.2110640	−2.0978290	0.1027280
H	−6.3686580	−3.1891990	−0.0990040
C	−8.7992010	−0.8467730	0.2635080
H	−8.4556550	1.2665120	0.4732000
H	−8.8306780	−2.9858910	0.0368050
H	−9.8787720	−0.7576810	0.3232030
F	−0.2459400	6.0616020	−0.0863170
C	4.3883690	−1.0363560	0.0086690
C	4.9358050	−2.3229830	0.0043870
C	5.2435290	0.0659860	0.0865320
C	6.3072460	−2.5108400	0.0760780
H	4.2695080	−3.1745830	−0.0554850
C	6.6183820	−0.1064440	0.1590580
H	4.8177210	1.0613060	0.0878060
C	7.1245610	−1.3947570	0.1523960
H	6.7479580	−3.5008470	0.0745470
H	7.2957520	0.7373210	0.2187900
F	8.4559120	−1.5699300	0.2223270

Table S2. *Cont.*

**5c**			
C	−2.2654150	−1.4300690	−0.1161390
C	−1.4861030	−0.2918560	−0.0557500
C	−0.0789090	−0.3788570	−0.1306490
C	0.5346320	−1.6534250	−0.2182910
C	−0.2791600	−2.8103120	−0.2973330
C	−1.6391030	−2.7018070	−0.2532270
H	−1.9636550	0.6709160	0.0689650
C	0.8176210	0.7364760	−0.0853400
H	0.2116010	−3.7731560	−0.3784240
H	−2.2625920	−3.5871370	−0.3063240
C	2.6059960	−0.7127660	−0.0869630
N	2.1185790	0.5547770	−0.0367610
N	1.8828960	−1.8017300	−0.2076230
C	0.3545670	2.1469290	−0.0795190
C	0.9571820	3.0547860	0.7964190
C	−0.6201140	2.6074540	−0.9691520
C	0.5786290	4.3882440	0.8099160
H	1.7268160	2.7025730	1.4731790
C	−0.9997860	3.9431260	−0.9769810
H	−1.0686730	1.9277510	−1.6845740
C	−0.3969500	4.8069770	−0.0799730
H	1.0268110	5.1010520	1.4918960
H	−1.7444280	4.3178140	−1.6689870
C	−3.6881510	−1.3452140	−0.0333730
C	−4.8936450	−1.2821120	0.0363680
C	−6.3185830	−1.2025690	0.1200310
C	−7.0987030	−2.3646140	0.0484390
C	−6.9505160	0.0387170	0.2748470
C	−8.4813440	−2.2825180	0.1302970
H	−6.6113030	−3.3258510	−0.0712720
C	−8.3337130	0.1117520	0.3559940
H	−6.3483290	0.9387670	0.3307750
C	−9.1020530	−1.0464030	0.2840180
H	−9.0772730	−3.1872790	0.0739100
H	−8.8143470	1.0768940	0.4760640
H	−10.1833640	−0.9857360	0.3477250
F	−0.7655610	6.0986930	−0.0762800
C	4.0819890	−0.8553880	−0.0180020
C	4.6682050	−2.1232510	−0.0294200
C	4.9031930	0.2706150	0.0617980
C	6.0452370	−2.2689880	0.0372750
H	4.0300480	−2.9960500	−0.0906960
C	6.2830050	0.1390910	0.1293730
H	4.4497330	1.2536420	0.0688300
C	6.8401550	−1.1322250	0.1163570
H	6.4989540	−3.2528430	0.0289480
H	6.9195960	1.0136550	0.1903870
Cl	8.5800670	−1.3073720	0.2012170

Table S2. *Cont.*

5d			
C	−2.2380430	−1.4350860	−0.1218500
C	−1.4826950	−0.2808790	−0.0592010
C	−0.0741250	−0.3373300	−0.1350460
C	0.5681900	−1.5973690	−0.2258200
C	−0.2220330	−2.7710420	−0.3077890
C	−1.5839840	−2.6923420	−0.2625670
H	−1.9805270	0.6713060	0.0681850
C	0.7992760	0.7962870	−0.0874870
H	0.2891230	−3.7230310	−0.3918140
H	−2.1879210	−3.5910950	−0.3177370
C	2.6224780	−0.6138970	−0.0913760
N	2.1029720	0.6437630	−0.0396600
N	1.9184170	−1.7175510	−0.2158640
C	0.3051760	2.1968680	−0.0781280
C	0.8872520	3.1153100	0.8005620
C	−0.6788920	2.6388540	−0.9666900
C	0.4798440	4.4403040	0.8177020
H	1.6645290	2.7778540	1.4761190
C	−1.0878400	3.9660180	−0.9708510
H	−1.1124430	1.9514310	−1.6840090
C	−0.5043390	4.8402830	−0.0712610
H	0.9123290	5.1607810	1.5017990
H	−1.8403520	4.3262700	−1.6620460
C	−3.6623450	−1.3808720	−0.0376460
C	−4.8688900	−1.3437080	0.0337860
C	−6.2951360	−1.2944990	0.1192460
C	−6.9536220	−0.0665920	0.2704180
C	−7.0505870	−2.4730830	0.0530450
C	−8.3379830	−0.0228330	0.3532130
H	−6.3708400	0.8464040	0.3221930
C	−8.4345940	−2.4203070	0.1365650
H	−6.5430050	−3.4241820	−0.0638470
C	−9.0816100	−1.1973090	0.2865790
H	−8.8388950	0.9323220	0.4703890
H	−9.0110010	−3.3379000	0.0843350
H	−10.1638910	−1.1595820	0.3515570
F	−0.9011980	6.1239380	−0.0640080
C	4.0957240	−0.7215180	−0.0189920
C	4.7175790	−1.9698770	−0.0288610
C	4.8998400	0.4224920	0.0648920
C	6.0988040	−2.0906740	0.0421260
H	4.1015350	−2.8588090	−0.0924800
C	6.2749580	0.3173060	0.1363490
H	4.4270720	1.3967650	0.0714870
C	6.8860670	−0.9408880	0.1256940
H	6.5480160	−3.0752410	0.0329530
H	6.9037960	1.1983390	0.2004550
O	8.2387550	−0.9422730	0.2000890
C	8.9116220	−2.1904380	0.1931200
H	9.9742850	−1.9614240	0.2606650
H	8.6195030	−2.8067980	1.0495900
H	8.7191480	−2.7432150	−0.7321590

Table S2. *Cont.*

5e			
C	1.9948780	−1.6712720	0.1039230
C	1.1743470	−0.5610280	0.0667680
C	−0.2286580	−0.6990730	0.1409540
C	−0.7957500	−1.9964700	0.1981920
C	0.0596270	−3.1244800	0.2550650
C	1.4150020	−2.9668580	0.2152910
H	1.6165240	0.4205760	−0.0394190
C	−1.1647090	0.3866750	0.1215080
H	−0.3962640	−4.1059520	0.3145620
H	2.0696780	−3.8303650	0.2509310
C	−2.8995310	−1.1300920	0.0733510
N	−2.4584580	0.1550340	0.0592760
N	−2.1375620	−2.1949980	0.1768570
C	−0.7538070	1.8092290	0.1583340
C	−1.4076750	2.7306540	−0.6592080
C	0.2296930	2.2829800	1.0371780
C	−1.0798120	4.0796740	−0.6392000
H	−2.1846270	2.3789950	−1.3285750
C	0.5554460	3.6254390	1.0791840
H	0.7233220	1.6005170	1.7199620
C	−0.0898360	4.5331280	0.2339910
H	−1.5979870	4.7627160	−1.2995330
H	1.3051540	3.9988670	1.7673880
C	3.4137850	−1.5337960	0.0227120
C	4.6164670	−1.4280440	−0.0449160
C	6.0380960	−1.3003990	−0.1259690
C	6.6299440	−0.0369850	−0.2587280
C	6.8554740	−2.4376600	−0.0739590
C	8.0101240	0.0819210	−0.3376280
H	5.9990860	0.8440640	−0.2992970
C	8.2347780	−2.3097700	−0.1533410
H	6.3992320	−3.4160170	0.0287120
C	8.8154870	−1.0518820	−0.2852450
H	8.4593990	1.0640260	−0.4404950
H	8.8594450	−3.1958020	−0.1121650
H	9.8943000	−0.9553230	−0.3470990
C	−4.3678150	−1.3248580	−0.0137520
C	−4.9066010	−2.6147120	−0.0445380
C	−5.2301860	−0.2268880	−0.0695040
C	−6.2763550	−2.8100460	−0.1292610
H	−4.2346470	−3.4629050	−0.0016550
C	−6.6034580	−0.4066300	−0.1547030
H	−4.8106890	0.7707820	−0.0434250
C	−7.1008690	−1.6980210	−0.1830580
H	−6.7102420	−3.8027580	−0.1549100
H	−7.2862630	0.4338020	−0.1976050
F	−8.4307830	−1.8803500	−0.2655290
O	0.3069140	5.8229840	0.3392560
C	−0.3210470	6.7904200	−0.4866800
H	0.1437530	7.7441740	−0.2410890
H	−0.1618710	6.5731510	−1.5478290
H	−1.3956660	6.8495430	−0.2862280

Table S2. *Cont.*

5f			
C	2.2508630	−1.6963270	0.1098240
C	1.4751450	−0.5543090	0.0728510
C	0.0681330	−0.6360030	0.1533460
C	−0.5494660	−1.9099020	0.2165670
C	0.2601310	−3.0709980	0.2730180
C	1.6205570	−2.9675860	0.2272770
H	1.9557130	0.4084370	−0.0385710
C	−0.8232340	0.4865790	0.1347200
H	−0.2344360	−4.0332120	0.3371830
H	2.2404990	−3.8563290	0.2626910
C	−2.6160210	−0.9597980	0.0978660
N	−2.1257660	0.3063460	0.0781230
N	−1.8984580	−2.0544770	0.2010040
C	−0.3558020	1.8914230	0.1658780
C	−0.9774780	2.8361120	−0.6503410
C	0.6508420	2.3278490	1.0378920
C	−0.5967180	4.1711440	−0.6354340
H	−1.7712570	2.5138860	−1.3147420
C	1.0293730	3.6564920	1.0747480
H	1.1211620	1.6280190	1.7195480
C	0.4153270	4.5871440	0.2311700
H	−1.0913950	4.8725830	−1.2945140
H	1.7970920	4.0015920	1.7578490
C	3.6736290	−1.6147100	0.0221240
C	4.8790750	−1.5541770	−0.0512140
C	6.3039800	−1.4775210	−0.1385710
C	7.0805430	−2.6434130	−0.0968440
C	6.9397480	−0.2351510	−0.2669420
C	8.4631650	−2.5640940	−0.1819400
H	6.5903400	−3.6055580	0.0024450
C	8.3229050	−0.1648750	−0.3515240
H	6.3404500	0.6679750	−0.2996030
C	9.0876310	−1.3268770	−0.3092760
H	9.0561530	−3.4719490	−0.1487140
H	8.8063410	0.8012340	−0.4509040
H	10.1689160	−1.2683660	−0.3755280
C	−4.0925600	−1.0967450	0.0175290
C	−4.6825060	−2.3627210	−0.0027520
C	−4.9103340	0.0329320	−0.0421860
C	−6.0595520	−2.5030900	−0.0812030
H	−4.0469100	−3.2383280	0.0432200
C	−6.2901140	−0.0929740	−0.1212750
H	−4.4536220	1.0143130	−0.0242810
C	−6.8508280	−1.3626220	−0.1400470
H	−6.5160000	−3.4856050	−0.0975250
H	−6.9239420	0.7845710	−0.1666830
O	0.8629230	5.8604970	0.3312660
C	0.2664670	6.8508390	−0.4910080
H	0.7699690	7.7860120	−0.2503630
H	0.4085730	6.6264170	−1.5530470
H	−0.8033730	6.9517230	−0.2819780
Cl	−8.5909110	−1.5308350	−0.2397660

Table S2. *Cont.*

5g			
C	2.2147030	−1.7070300	0.1166490
C	1.4656180	−0.5474020	0.0765150
C	0.0572420	−0.5953940	0.1581340
C	−0.5918790	−1.8533020	0.2256370
C	0.1914810	−3.0328350	0.2862260
C	1.5539380	−2.9622450	0.2391410
H	1.9686730	0.4034610	−0.0386180
C	−0.8083890	0.5471760	0.1365700
H	−0.3255810	−3.9829480	0.3544280
H	2.1523230	−3.8656190	0.2775390
C	−2.6389710	−0.8560700	0.1028170
N	−2.1139080	0.3989160	0.0806470
N	−1.9429670	−1.9667890	0.2109300
C	−0.3073260	1.9412930	0.1644280
C	−0.9066270	2.8985070	−0.6536890
C	0.7091320	2.3561700	1.0353500
C	−0.4951640	4.2245870	−0.6413820
H	−1.7080710	2.5931440	−1.3168360
C	1.1185470	3.6758860	1.0696140
H	1.1634260	1.6465670	1.7177970
C	0.5260390	4.6188170	0.2244820
H	−0.9735850	4.9360300	−1.3017730
H	1.8941810	4.0043860	1.7520000
C	3.6390120	−1.6593980	0.0270340
C	4.8455060	−1.6279470	−0.0485260
C	6.2717140	−1.5853670	−0.1383920
C	6.9372220	−0.3583730	−0.2644360
C	7.0204260	−2.7695270	−0.1014510
C	8.3215300	−0.3210270	−0.3513660
H	6.3597860	0.5590260	−0.2934100
C	8.4044400	−2.7231780	−0.1889130
H	6.5075690	−3.7199920	−0.0039640
C	9.0583810	−1.5010570	−0.3138600
H	8.8277320	0.6335640	−0.4488730
H	8.9754180	−3.6451840	−0.1593730
H	10.1406410	−1.4683310	−0.3819450
C	−4.1129410	−0.9548920	0.0194780
C	−4.7412970	−2.1998160	0.0035980
C	−4.9108520	0.1943400	−0.0494210
C	−6.1227620	−2.3123240	−0.0781720
H	−4.1299420	−3.0927240	0.0558330
C	−6.2861030	0.0975730	−0.1316770
H	−4.4327300	1.1659290	−0.0354850
C	−6.9036650	−1.1573370	−0.1468150
H	−6.5769460	−3.2946180	−0.0887360
H	−6.9100430	0.9828760	−0.1842860
O	1.0027160	5.8822710	0.3223930
C	0.4272450	6.8843140	−0.5003220
H	0.9508560	7.8087720	−0.2610390
H	0.5636050	6.6560100	−1.5623200
H	−0.6401090	7.0084140	−0.2908560
O	−8.2561660	−1.1504330	−0.2297340
C	−8.9351050	−2.3950820	−0.2465430
H	−8.7508900	−2.9633780	0.6710110
H	−9.9962510	−2.1598590	−0.3168490
H	−8.6408900	−2.9994860	−1.1108140

Table S2. *Cont.*

**5h**			
C	−2.3889690	−1.7570480	−0.4424550
C	−1.5641530	−0.6579150	−0.3141280
C	−0.1595510	−0.8063220	−0.3002830
C	0.4009160	−2.1058550	−0.3656200
C	−0.4571470	−3.2226470	−0.5171640
C	−1.8113970	−3.0527310	−0.5598300
H	−2.0048430	0.3238500	−0.2030510
C	0.7811040	0.2683040	−0.1808480
H	−0.0057390	−4.2060280	−0.5802680
H	−2.4688420	−3.9082780	−0.6665640
C	2.4991900	−1.2648280	−0.0724570
N	2.0664220	0.0231870	−0.0422830
N	1.7373870	−2.3175010	−0.2631190
C	0.3829240	1.6954490	−0.1933390
C	0.9829090	2.5803710	0.7018980
C	−0.5335700	2.2095390	−1.1204160
C	0.6656550	3.9322100	0.7091020
H	1.7085210	2.1973690	1.4105430
C	−0.8471450	3.5555830	−1.1354510
H	−0.9832750	1.5559750	−1.8595750
C	−0.2571660	4.4259990	−0.2141490
H	1.1402160	4.5860150	1.4291560
H	−1.5444590	3.9605760	−1.8599750
C	−3.8129600	−1.6095300	−0.4483380
C	−5.0147280	−1.5003600	−0.4560270
C	3.9588760	−1.4776340	0.1047610
C	4.4840590	−2.7727630	0.1218530
C	4.8229570	−0.3913370	0.2601910
C	5.8462320	−2.9759930	0.2912330
H	3.8089100	−3.6113300	0.0016280
C	6.1861230	−0.5971350	0.4297670
H	4.4122030	0.6104680	0.2447940
C	6.7019490	−1.8887990	0.4458600
H	6.2427320	−3.9860500	0.3034480
H	6.8481310	0.2544430	0.5487850
O	−0.6374700	5.7226480	−0.2993330
C	−0.0617900	6.6538680	0.6025870
H	−0.5014230	7.6202790	0.3600290
H	−0.2960700	6.4007880	1.6416870
H	1.0246250	6.7098010	0.4797090
H	7.7671540	−2.0486370	0.5783360
C	−6.4693020	−1.3502240	−0.4597330
H	−6.7447530	−0.4581680	−1.0325080
H	−6.9251120	−2.2070380	−0.9672950
C	−7.0712640	−1.2378570	0.9368770
H	−6.6192900	−0.3883460	1.4650810
H	−8.1467880	−1.0381880	0.8365280
O	−6.8336040	−2.4553270	1.6170570
H	−7.1524900	−2.3599560	2.5221840
